# Bioprinting revolution: Innovative design of 3D bioactive scaffolds for living organs and transdermal tissues

**DOI:** 10.1002/btm2.70080

**Published:** 2025-09-28

**Authors:** Seydanur Yücer, Begüm Sarac, Fatih Ciftci

**Affiliations:** ^1^ Faculty of Engineering, Department of Biomedical Engineering Fatih Sultan Mehmet Vakıf University Istanbul Turkey; ^2^ Biomedical Electronic Design Application and Research Center (BETAM) Fatih Sultan Mehmet Vakıf University Istanbul Turkey; ^3^ BioriginAI Research Group, Department of Biomedical Engineering Fatih Sultan Mehmet Vakıf University Istanbul Turkey

**Keywords:** 3D bioactive scaffolds, bioprinting, innovative design, living organs, transdermal tissues

## Abstract

The bioprinting revolution has opened new frontiers in the design and fabrication of three‐dimensional bioactive scaffolds for living organs and transdermal tissues. This transformative technology enables the precise assembly of biomaterials and living cells, creating structures that mimic the complexity of natural tissues. Advances in bioprinting have facilitated the development of personalized scaffolds for tissue regeneration, wound healing, and transdermal drug delivery systems. This abstract explores cutting‐edge innovations in bioactive scaffold design, highlighting their potential to replicate functional organ structures and support transdermal therapeutic applications. Current challenges, such as achieving vascularization and mechanical integrity, are discussed alongside emerging solutions and future directions for clinical translation. Bioprinting stands poised to redefine the landscape of regenerative medicine and transdermal therapeutics.

**Translational Impact Statement**

This study presents advanced three‐dimensional bioprinting strategies for engineering bioactive scaffolds with high biomimicry, addressing critical limitations in tissue regeneration and transdermal drug delivery. By integrating next‐generation bioinks and design innovations, the proposed approaches enhance vascularization, mechanical stability, and functional integration, offering clinically relevant solutions for personalized regenerative therapies and organ‐level reconstruction.

## 1 INTRODUCTION

Bioprinting is a transformative additive manufacturing (AM) method that enables the construction of structured objects using biomaterials, either with or without cells. The origins of this technology date back to 1988, when cells were initially placed on surfaces through a standard inkjet printer. Over the years, bioprinting has made significant strides, progressing through numerous technological advancements.^1^ Bioprinting reached a significant milestone in 1999 with the introduction of laser‐assisted technology, enabling the creation of complex anatomical structures. This was followed by the development of extrusion‐based bioprinting in 2002 and the advent of electrohydrodynamic spraying in 2006. These advancements paved the way for breakthroughs in regenerative medicine, presenting exciting opportunities for the future of healthcare.^2^ The increasing global need for organ transplants has led the healthcare industry to investigate the creation of artificial organs and tissues. In this regard, three‐dimensional (3D) bioprinting has become a crucial technology, offering the potential to provide tailored treatment solutions.^3^ Unlike traditional methods, bioprinting does not require pre‐optimized biomaterials. This technique enables the swift creation of cost‐effective and efficient solutions through the use of advanced imaging and AM technologies. By allowing for the development of personalized treatment options for specific diseases, applications, and individual needs, bioprinting has the potential to revolutionize essential areas of healthcare, such as organ transplantation and drug development.^4^ These technological advancements have not only revolutionized life‐saving applications like organ transplantation but have also made a substantial impact on disease modeling and research. For example, the creation of 3D vascularized tumor models (organ‐on‐a‐chip) has advanced the study of cancer metastasis in multiple organs and the signaling mechanisms involved in breast cancer. These models provide deeper insights into complex diseases such as cancer, paving the way for personalized treatment strategies. Bioprinting's ability to accelerate both research and treatment development demonstrates its significant role in advancing healthcare innovations.^5^ In addition, bioprinting technology plays a crucial role in the creation of specialized tissues, such as transdermal (subcutaneous) tissues. The potential of 3D bioprinting spans a wide range of applications, including drug delivery, cosmetic treatments, and skin regeneration.^6^, ^7^ By layering skin cells and biological structures, bioprinters can provide personalized treatment solutions and improve the accuracy of skin therapies.

Over the last two decades, significant progress has been made in the field, driven by innovations in smart bioinks, advanced printing methods, and biomaterials. These developments have enabled the production of custom tissue‐engineered constructs and complex organs in just 1 day. This rapid advancement has also spurred commercial growth, with many bioprinting startups emerging since 2014. In 2016, the market value was estimated at approximately 680 million USD, reflecting the growing significance of bioprinting in both the medical and economic sectors. These advancements demonstrate that bioprinting has the potential to revolutionize healthcare and redefine the future of personalized treatments and organ transplantation.^8^

## 2 BUILDING LIVING TISSUES WITH 3D BIOPRINTING: FUNDAMENTALS AND FUTURE TECHNOLOGIES

### 2.1 The basics of 3D bioprinting: printers and bioinks

3D bioprinting involves the precise arrangement of biological materials, living cells, and biochemicals to form 3D structures. This technique is typically carried out using three primary approaches: biomimicry, autonomous self‐assembly, and mini tissue building blocks. Biomimicry focuses on studying natural systems to create solutions for human‐related challenges. The integration of biomimetic elements into bioprinted structures can significantly impact various aspects of cell behavior, such as attachment, migration, proliferation, and adhesion. The choice of materials used in bioprinting plays a critical role in influencing cell activities like proliferation and differentiation, affecting factors such as cell adhesion, shape, and size. Additionally, nanoscale features have been shown to influence cell attachment and the organization of the cytoskeleton. Autonomous self‐assembly mimics the organ development process seen in embryos, facilitating tissue growth under laboratory conditions. This method leverages cellular components to organize tissue by producing extracellular matrix (ECM) components and signaling molecules. In tissue engineering (TE), 3D porous scaffolds are essential for supporting cell attachment, proliferation, and the production of ECM. Bioprinting is commonly used to create these scaffolds. The functionality of bioprinting technologies is supported by a range of printing techniques, such as droplet‐based bioprinting, extrusion‐based bioprinting, and laser‐assisted bioprinting (LAB), all of which are widely used in the field.

1. Inkjet bioprinting operates based on the same concept as conventional inkjet printers, where bioink is sprayed onto a hydrogel (HG) surface or culture in a non‐contact process. This method is enabled by the use of thermal or piezoelectric actuators, which control the precise dispensing of bioink.
2. In extrusion‐based bioprinting, biomaterials are forced through a microscale nozzle or microneedle (MN), forming a continuous filament. This is achieved through pneumatic or mechanical pressure from a plunger or screw. The bioink is deposited layer by layer, creating complex 3D patterns and structures.
3. LAB uses a laser to deposit biomaterials onto a substrate. The system involves three main components: (i) a pulsed laser source, (ii) a ribbon coated with liquid biological materials, which is deposited onto a metal film, and (iii) a receiving substrate containing biopolymers or a cell culture medium to support cell adhesion and growth. When the laser interacts with the ribbon, it causes the evaporation of the liquid materials, transferring them onto the substrate.^9^

The success of bioprinting techniques heavily relies on the careful selection of bioinks. Bioinks are specialized materials that enable the printing of living cells and biomolecules, ensuring their proper transport and organization during the 3D printing process. A critical aspect of bioink development is creating an environment that promotes cell adhesion, proliferation, and optimal functionality. Choosing the right materials is crucial, with both natural and synthetic polymers being widely used. The effectiveness of organ and TE depends on the biocompatibility, biological interactions, and compatibility of the materials with the printing process. These materials must be non‐toxic, bioactive, and capable of being processed at low temperatures. Common examples of bioinks include natural and synthetic polymers, such as gelatin, hyaluronic acid (HA), collagen, poly(ε‐caprolactone) (PCL), polyethylene glycol (PEG), and Pluronics. Additionally, elastomers, ceramics, and HGs play vital roles in 3D printing applications. The success of tissue and organ engineering depends on the versatility and biocompatibility of these materials. Polymeric materials are particularly favored as bioinks due to their compatibility with biological systems, degradability, and cost‐efficiency. Their adaptability, especially in powder form for techniques like laser bioprinting or fused deposition modeling (FDM), offers significant benefits. HGs and water‐soluble polymers are also commonly used due to their ability to support cells and maintain high water retention properties. Advancements in bioink development are essential for fostering innovation in 3D bioprinting technologies. A variety of biopolymers, including collagen, gelatin, alginate, HA, and chitosan, are being explored for their potential in these applications. Among these, collagen stands out as one of the most commonly used bioinks. Collagen, a natural protein in the ECM of various tissues, plays a critical role in musculoskeletal tissue structure. Collagen‐based scaffolds are preferred due to their ability to minimize immune responses while supporting cell growth, adhesion, and attachment. However, despite its benefits, type I collagen, a frequently used material in 3D bioprinting, has certain drawbacks. For example, it remains in a liquid state at lower temperatures and takes up to 30 min to fully gel at 37°C, which can hinder the printing process. HA, another key bioink in 3D bioprinting, is a naturally occurring component of the ECM. Typically sourced from bovine tissue, HA is appreciated for its biodegradability, biocompatibility, and non‐immunogenic, non‐thrombogenic, and non‐adhesive properties. Its ability to form flexible HGs makes HA a promising material for 3D bioprinting, particularly in applications such as cartilage TE for repair and regeneration. Recent research has highlighted the potential of using HA as a bioink for creating structures that support tissue regeneration, positioning it as a valuable area of study in the field of bioprinting.^10^ This study thoroughly evaluated the printability, biocompatibility, and biodegradability of HA. The findings showed that integrating HA into a polylactic acid (PLA) bioink improved the mechanical properties of the printed cartilage structures. Additionally, the inclusion of HA in the bioink composition was found to enhance cellular activity, promoting the expression of cartilage‐related genes and boosting ECM production. These results emphasize the significant potential of HA‐based bioinks in advancing 3D bioprinting for cartilage TE.^11^ Gelatin is a key biomaterial widely utilized in TE and regenerative medicine, alongside HA. This natural protein exhibits amphoteric behavior due to the presence of both acidic and basic amino acid groups. It is obtained by hydrolyzing collagen, with mammalian‐derived gelatin commonly employed in regenerative medicine. Gelatin is highly prized for its biocompatibility, water solubility, non‐toxicity, and its capacity to support cell adhesion. Its biodegradable nature and minimal immunogenic response make it especially suitable for biomedical applications. A prominent use of gelatin HGs is in Direct Ink Writing (DIW) 3D printing, particularly when modified into gelatin methacryloyl (GelMA).^12^ This study led to the development of a bio‐binder, combining silk fibroin and gelatin, which eliminates the need for chemical binders. Tailored for 3D bioprinting, this bio‐binder shows potential for producing cartilage tissue substitutes. Additionally, the developed bioink boasts improved rheological properties, which enhance printing efficiency and present promising characteristics for advancing TE applications.^13^ Chitosan, a naturally derived polymer, is valued for its biocompatibility and antibacterial properties, making it useful in TE. However, its application in hard TE is limited due to its inherent mechanical shortcomings.^11^ To overcome this challenge, a chitosan‐based bioink for 3D printing was developed. The chitosan was modified using ethylenediaminetetraacetic acid (EDTA), with cross‐linking achieved through the addition of calcium. Mechanical testing showed an improvement in the viscoelastic properties of the modified chitosan. Both modified and unmodified chitosans were then combined to create the bioink, which was used to fabricate scaffolds. In vitro experiments demonstrated that the bioink supported cell attachment, exhibited favorable mechanical properties, and promoted the expression of collagen type 2, a key gene for cartilage formation. These results suggest that this bioink holds significant potential for cartilage TE. While natural polymers like chitosan offer promising environments for cell adhesion, proliferation, and differentiation, their mechanical stability and degradation rates can present challenges.^14^ In addition to the advantages provided by natural polymers, synthetic polymers play an essential role in bioprinting processes. These polymers are often chosen for their stability and the ability to precisely control their properties in bioink formulations. Some widely used synthetic polymers in bioprinting include PLA, PEG, PCL, and polyvinyl alcohol (PVA). These materials offer vital characteristics such as biocompatibility, biodegradability, flexibility, and the necessary mechanical strength to support cell growth structures. PLA is particularly popular, especially in FDM, and is commonly used to create bioabsorbable materials. However, it is important to note that PLA degrades over time, releasing acidic by‐products that could affect its long‐term compatibility. To mitigate this, PLA can be combined with low‐cost ceramic materials to improve scaffold strength and minimize the formation of acidic by‐products. PEG, another synthetic polymer, is hydrophilic and produced via radical polymerization. Its linear or branched structure, along with asymmetric or dissymmetric hydroxyl groups, enhances its suitability and flexibility in various bioprinting applications. PEG is well‐regarded for its excellent biocompatibility and is widely used in applications such as drug delivery systems, TE scaffolds, and surface modifications. PEG is particularly known for its resistance to protein adsorption and cell adhesion, and it commonly forms HGs, making it highly suitable for various biomedical applications. On the other hand, PCL is a more affordable polymer that offers significant benefits in bioink formulations, including mechanical stiffness, biocompatibility, and biodegradability. PCL is non‐toxic and retains its stability for extended periods, typically up to 3 years in biological environments. Research has shown that PCL scaffolds produced using selective laser sintering (SLS) are effective in supporting bone regeneration and cell growth. However, PCL's prolonged biological half‐life can limit its suitability for certain uses. Additionally, its high hydrophobicity can decrease bioactivity and slow down cell proliferation. PVA, a water‐soluble polymer, stands out due to its ability to form complex structures with excellent adhesive properties and create an ideal matrix for bone cell growth. PVA's hydrophilic characteristics, chemical stability, and semi‐crystalline structure enhance its capacity for efficient oxygen and nutrient transfer to cells. Its water solubility also makes it a promising material for load‐bearing therapies, such as craniofacial defect repair and bone TE, thanks to its ability to control swelling and resist harsh conditions.^12^

In summary, 3D bioprinting is a technique that involves the meticulous arrangement of biological materials, cells, and biochemicals to create 3D structures. Key approaches in this field include biomimicry, autonomous self‐assembly, and the use of miniaturized tissue building blocks. The success of this technology depends on the careful selection of bioinks, which can be sourced from natural polymers such as collagen, HA, and gelatin, or synthetic polymers like PLA, PEG, PCL, and PVA. While natural polymers promote cell adhesion and growth, they often lack the mechanical strength required for some applications. In contrast, synthetic polymers offer greater durability and enable more precise control over their physical properties. Recent innovations in bioink formulations, such as cross‐linker‐free blends of silk and gelatin, demonstrate significant potential, particularly in cartilage TE. These advancements significantly enhance the potential of 3D bioprinting in advancing medical applications and TE.

### 2.2 Material selection and next‐generation bioinks

**FIGURE 1:**
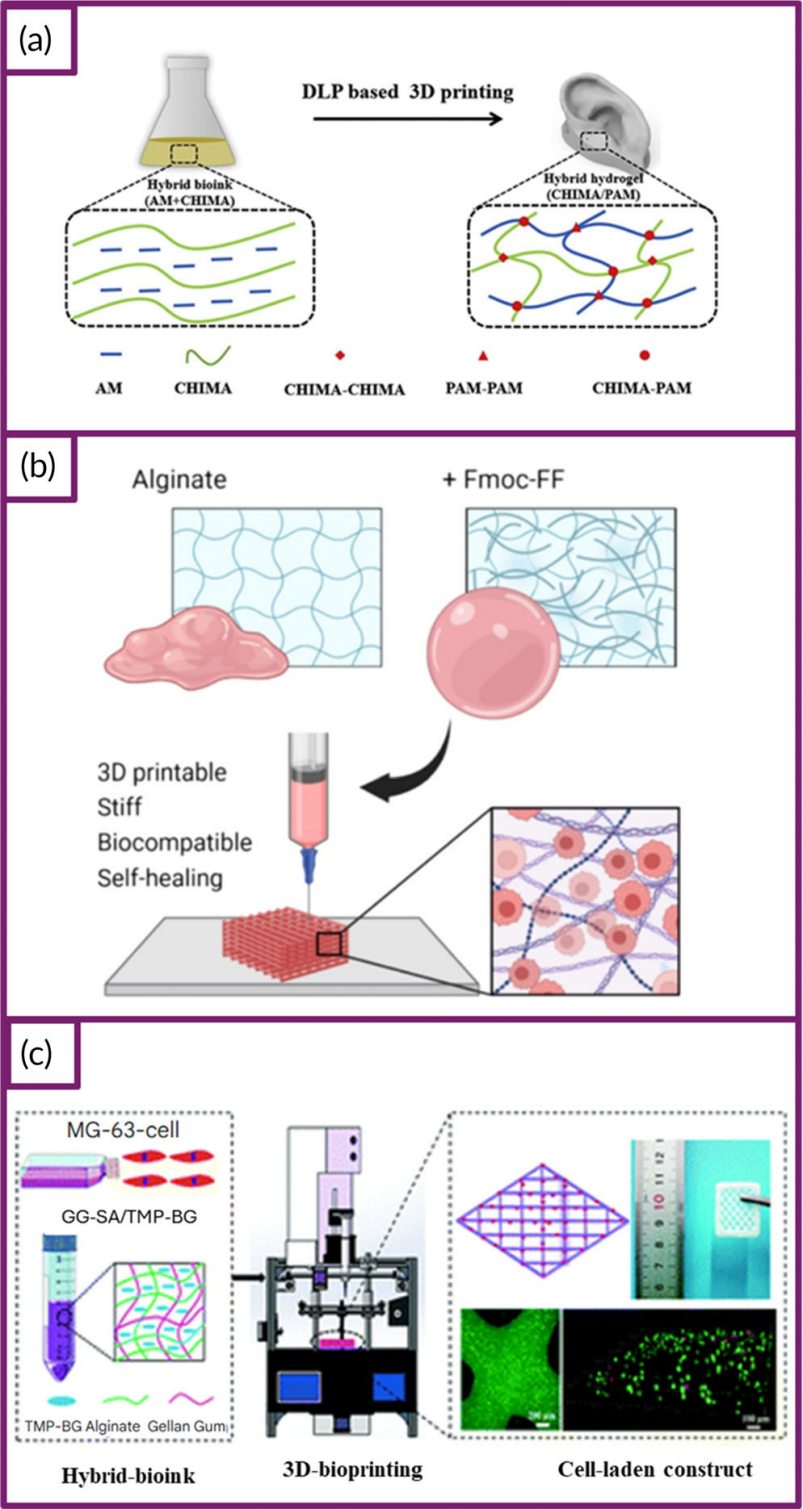
Schematic summaries of different hybrid bioink formulations for improved biocompatibility, mechanical strength, and printability in 3D bioprinting. (a) Schematic summary demonstrates that the light‐curable hybrid bioink derived from chitosan derivative (CHIMA) and acrylamide offers a promising approach to fabricate biocompatible and mechanically strong hydrogel structures in digital light processing (DLP)‐based three‐dimensional (3D) bioprinting.^19^ (b) Schematic summary demonstrates the development of biocompatible, tough, printable, and self‐healing bioinks by hybridizing self‐assembling peptides containing Fmoc‐diphenylalanine (Fmoc‐FF) with sodium alginate.^20^ (c) Schematic summary demonstrates that the hybrid bioink developed from the combination of gellan gum, sodium alginate, and magnesium phosphate‐based gel can be used for regenerative medicine applications in 3D bioprinting by offering high printability, biocompatibility, mechanical durability, and bioactivity.^21^ AM, acrylamide; GG–SA, gellan gum–sodium alginate; TMP‐BG, thixotropic magnesium phosphate‐based gel.

The composition of bioinks can vary depending on the choice of biomaterials, which directly affects their suitability for specific applications. In some cases, cell aggregates themselves may be used as bioinks. An ideal bioink should possess a variety of essential properties, including mechanical, rheological, chemical, and biological characteristics, to effectively support the fabrication of functional tissues and organs. These properties include the ability to maintain tissue‐specific mechanical traits, provide sufficient strength and stiffness, ensure controlled gelation, and retain stability for accurate shape fidelity during printing. Additionally, the bioink should be biocompatible, biodegradable, and capable of chemical modifications. It should also be amenable to large‐scale production. Natural bioinks are often preferred for their ability to closely mimic the properties and biological functions of native tissues, along with their cytocompatibility.^15^ In recent years, AM in the biomedical field has garnered significant interest due to its potential to provide customized solutions. Various bioinks have been developed to create specialized formulations that cater to the needs of applications such as TE and drug delivery. However, combining printability, cytocompatibility, and biofunctionality in a single bioink remains a significant challenge. To overcome this, researchers have investigated synthetic approaches and the functionalization of nanoparticles with different capabilities. These nanoparticles are integrated into polymeric systems, resulting in the development of multifunctional bioinks.^16^ For example, this study focused on the development and characterization of smart bioinks composed of sodium alginate, poly(*N*‐isopropylacrylamide) (PNIPAm), and ZnSO_4_. Key properties, including the lower critical solution temperature (LCST), viscosity, and thermal stability, were assessed using UV–Visible spectroscopy, rheological analysis, and thermogravimetric analysis (TGA), respectively. Additionally, an injectability test was performed to evaluate how the extrusion process affected the morphology of the bioinks and the resulting scaffolds after lyophilization. Scanning electron microscopy (SEM) was employed to examine the scaffold structures closely. The results indicated that the scaffolds exhibited an interconnected pore network, influenced by the Zn^2+^ ions and the extrusion process. To assess biocompatibility, a hemolysis assay using erythrocytes was conducted, showing no signs of toxicity. These findings highlight the potential of these bioinks for use in bioprinting and TE applications.^17^ In a similar study, a bioink composed of HA and dopamine was developed for 3D bioprinting of corneal equivalents, utilizing hydrazone cross‐linking chemistry. The bioink was initially optimized to improve its shear‐thinning behavior, viscosity, and mechanical stability to ensure reliable shape fidelity and self‐healing properties during extrusion‐based 3D printing. Human adipose stem cells (hASCs) and hASC‐derived corneal stromal keratocytes were incorporated to fabricate corneal stroma constructs. The viability, proliferation, microstructure, and expression of important proteins, including lumican, vimentin, connexin 43, and α‐smooth muscle actin (α‐SMA), were evaluated to assess cellular function. Additionally, the bioprinted stromal constructs were implanted into ex vivo porcine corneas to evaluate tissue integration. Human pluripotent stem cell‐derived neurons (hPSC‐neurons) were also bioprinted around the corneal constructs to investigate innervation. The bioink exhibited excellent shear‐thinning properties, viscosity, printability, shape fidelity, and self‐healing capabilities, as well as high cytocompatibility. The printed constructs showed effective tissue formation and successfully integrated with the host tissue in ex vivo models, with in vitro innervation observed. These results highlight the promising potential of this bioink and the 3D‐printed corneal stromal equivalents for applications in corneal TE.^18^ In this other study, a hybrid bioink consisting of photocurable chitosan and acrylamide (AM) was created for digital light processing (DLP)‐based 3D bioprinting in TE. The bioink was formulated by combining AM with CHIMA, a chitosan derivative modified with methacryloyl groups. The gelation behavior of the pre‐HG bioink was mainly determined by the type and concentration of photoinitiators used. By adjusting the AM content, the mechanical properties and cytocompatibility of the resulting HG could be tailored. The synergy between the natural CHIMA and synthetic AM endowed the hybrid HGs with both biological activity and mechanical strength. DLP‐based 3D printing enabled the production of intricate 3D HG structures that exhibit high strength and excellent biocompatibility. Therefore, the photocurable hybrid bioink, composed of CHIMA and AM in carefully balanced proportions, holds significant potential for advancing tissue and organ engineering applications (Figure 1a).^19^

In this similar study, a hybrid material was developed by embedding self‐assembled Fmoc‐diphenylalanine (Fmoc‐FF) assemblies within a sodium alginate matrix. The impact of different organic solvents used to dissolve the peptides was examined. The results demonstrated that bioinks prepared with peptides dissolved in 1,1,1,3,3,3‐hexafluoro‐2‐propanol (HFIP) showed better biocompatibility compared to those made using dimethyl sulfoxide (DMSO). This improvement is likely due to the higher volatility and lower surface tension of HFIP, which promotes more efficient solvent removal. By optimizing both the assembly and solvent conditions, hybrid bioinks were produced with stiffness up to eight times greater than pure sodium alginate, while still maintaining excellent printability, even with high cell concentrations. Additionally, the shear‐thinning properties of the self‐assembled peptide structures contributed self‐healing capabilities to the bioinks. These improvements make the hybrid bioinks ideal for bioprinting applications, particularly in TE and biosensors, where extrusion challenges and shear‐induced toxicity have previously been concerns (Figure 1b).^20^ This study highlights how integrating self‐assembling peptides with alginate can significantly enhance both mechanical and biological performance, pointing toward a broader trend of hybrid bioink development to overcome extrusion‐related limitations.

In a similar study, a novel hybrid bioink was created by combining gellan gum (GG), sodium alginate, and a thixotropic magnesium phosphate‐based gel (TMP‐BG). The proportions of these components were optimized to achieve desirable gelation, mechanical, rheological, and printability characteristics. The resulting GG–SA/TMP‐BG bioink exhibited excellent printability due to its shear‐thinning behavior and the cross‐linking potential of Mg^2+^ and Ca^2+^ ions. The bioink's mechanical properties were adjustable, allowing it to replicate the ECM of various tissues and maintain the integrity of 3D‐printed structures. Additionally, when immersed in simulated body fluids, the hybrid bioink facilitated apatite formation and supported cell growth in vitro. MG‐63 osteosarcoma cells were incorporated into the bioink and 3D‐printed into structures, where they demonstrated good cell viability, owing to the bioink's shear‐thinning properties and ion‐induced cross‐linking. The cells also exhibited a significantly higher proliferation rate compared to unprinted controls. Confocal microscopy revealed the uniform distribution of cells within the printed structures, with survival observed for more than 7 days. In vivo studies showed that the hybrid bioink, when used without cells, could support osteochondral repair. The bioink's superior printability, biocompatibility, mechanical properties, and bioactivity highlight its potential for use in 3D bioprinting applications (Figure 1c).^21^ When compared with peptide–alginate and GelMA/SFMA (Silk Fibroin Methacryloyl) systems, this study illustrates a complementary approach leveraging inorganic ions for cross‐linking to address both printability and bioactivity, underscoring the diversity of strategies pursued in hybrid bioink research.

Collectively, these studies highlight a unifying trend in bioink design: integrating natural and synthetic components to achieve an optimal balance between printability, biocompatibility, and mechanical integrity. Rather than viewing these bioinks in isolation, a broader synthesis suggests that rheological tuning, cross‐linking versatility, and bioactivity enhancement are converging as central strategies to overcome the traditional limitations of extrusion‐based bioprinting. This convergence indicates a shift from trial‐and‐error development toward a framework where solvent choice, cross‐linking chemistry, and bioactive incorporation are systematically engineered to address key challenges such as shape fidelity, cell viability, and long‐term stability.

### 2.3 Bioprinter design and innovative technologies

3D bioprinting technology holds significant promise for advancing medical applications. By utilizing bioinks, these printers can construct human tissues and organs through a layered approach, offering great potential in fields such as medical treatments, wound healing, and organ transplantation. The development of bioprinters is a multifaceted process that involves imaging and modeling, computer‐aided design (CAD), bioink preparation, overcoming challenges in the printing process, creating multi‐material and multicellular constructs, and incorporating techniques like Laser Guided Direct Writing (LGDW). The combination of these cutting‐edge technologies allows bioprinters to produce tissue and organ structures that are not only efficient but also biocompatible.

#### 2.3.1 Imaging and modeling

The bioprinting process begins with the biomodelling of the intended tissue or organ, starting with the processing of data obtained from several medical imaging techniques. These methods may include x‐ray computed tomography (CT), magnetic resonance imaging, ultrasound, single photon gamma ray imaging, and bioluminescence imaging. The next step involves using segmentation software to create a 3D model, and the surface geometry of this model is then converted into a Standard Tessellation Language (STL) file format. This file is then prepared for processing in CAD software.

#### 2.3.2 CAD operations and slicing

In CAD software, the STL file representing the target tissue or organ is sliced into individual layers. After slicing, a path planning process is performed for each layer, often using techniques like zigzag or contour‐offset. This step is crucial for defining the trajectory of the bioink dispenser during the 3D printing process.

#### 2.3.3 Bioink preparation

Bioink preparation is a critical phase before initiating the bioprinting process, especially in the context of patient‐specific tissue or organ creation. It is common to use primary cells from the patient, with stem cells, particularly mesenchymal stem cells (MSCs), often preferred for bioink formulation. Bioink deposition is typically achieved using droplet‐based spraying techniques, with two main methods: droplet‐on‐demand (DOD) and continuous spraying. DOD systems employ thermal, acoustic, or piezoelectric actuators to produce small droplets (ranging from 5 to 50 μm), where the material is rapidly heated to form vaporized bubbles that expand and release the droplets. Brief heating pulses (about 2 ms) are crucial for maintaining cell viability and preserving the material's properties. In continuous spraying, the material is dispensed continuously, and the spraying rate is controlled to achieve resolutions generally between 20 and 100 μm. Ink viscosity plays a vital role in print quality, as it can vary widely (1–100 mPa s). Ultrasonic nebulization is commonly used for low‐viscosity inks because it produces more uniform droplets, though it is not suitable for high‐viscosity inks. Pneumatic nebulization can handle a broader range of viscosities, but may result in polydisperse flows, which can lead to clogging of the nozzle.

#### 2.3.4 Extrusion‐based technologies

Extrusion‐based bioprinting encounters various challenges, including nozzle clogging and resolution limitations. In these techniques, material viscosity and processing parameters such as flow rate and deposition rate are crucial factors. Optimizing the rheological properties of bioinks, especially their shear‐thinning behavior, is key to improving print quality. Additionally, it is important to consider the negative effects of high pressure and mechanical stresses during printing, which can reduce cell viability. Recent advancements have addressed these challenges, such as the use of coagulation baths that allow for immediate gelation of materials during printing. Another notable development is the twin‐screw extruder system introduced by which helps reduce cell exposure to shear stress while improving bioink consistency through adjustable screw spacing.

#### 2.3.5 Multi‐material and multicellular structures

Photopolymerization has proven to be a highly effective method for bioprinting multi‐material and multicellular structures. One significant advancement is Bartolo's multi‐material stereolithography (SLA) system, which enables the selection of various materials from different cuvettes and their integration into a single cohesive structure. This approach has facilitated the creation of spatially organized multicellular structures through manual layering of polyethylene glycol diacrylate (PEGDA)‐based bioinks, providing fine control over the bioprinting process. These techniques have been crucial in advancing the creation of complex microenvironments, enhancing the overall precision and accuracy of bioprinting. Additionally, the method has been successfully used to fabricate intricate biomimetic constructs by strategically positioning various cell types within a single structure.

#### 2.3.6 Laser guided direct writing

LGDW is a technique that uses radiation pressure to precisely deposit bioink droplets onto a surface. This process relies on pressure waves generated by light, enabling the accurate placement of cells with micrometer‐level precision. LGDW has shown effectiveness in positioning sensitive cell types, such as embryonic chick spinal cord cells. However, issues like low cell yield and limited reproducibility of the process remain. To overcome these challenges, the use of hollow optical fibers has been proposed as a method to improve the transport distance of cells during the deposition process.^22^

Although 3D bioprinting technologies such as inkjet, extrusion, and laser‐assisted printing offer significant opportunities for TE, each method has inherent limitations that hinder its clinical translation. Inkjet bioprinting, while cost‐effective and suitable for high‐throughput printing, often struggles with nozzle clogging and limited viscosity ranges, reducing its applicability for more complex tissue constructs. Extrusion‐based printing, despite being the most widely adopted technique, frequently compromises cell viability due to high shear stress and limited resolution compared to laser‐assisted approaches. Conversely, LAB provides superior precision but remains expensive, technically complex, and less scalable. Similarly, bioinks derived from natural polymers promote biocompatibility and cell adhesion but lack sufficient mechanical strength, while synthetic polymers offer stability at the cost of reduced bioactivity. A major unresolved challenge lies in achieving the optimal balance between printability, biological functionality, and mechanical durability. Furthermore, the reproducibility of results across different bioprinting platforms remains inconsistent, posing barriers to regulatory approval and clinical standardization. These challenges highlight the urgent need for comparative studies, hybrid bioink formulations, and standardized protocols to bridge the gap between laboratory success and real‐world biomedical applications.

## 3 BIOACTIVE SCAFFOLDS: VITAL STRUCTURES FOR LIVING ORGANS

### 3.1 The role and importance of scaffolds

TE is a multidisciplinary field that focuses on repairing damaged tissues and developing biological replacements. A key focus within this field is the design of scaffolds that function effectively. In bone TE, scaffolds serve two main purposes: providing mechanical support and promoting vascularization. An ideal bone scaffold is characterized by high porosity, which is essential for supporting bone formation. The introduction of bioprinting technology has created new opportunities for producing customized scaffolds tailored to meet the specific needs of bone repair.^23^ While significant progress has been made in bone TE, organ engineering is also benefiting from advancements in bioprinting technology. 3D bioprinting, also known as organ printing, enables the creation of organs using microtissue spheroids. However, challenges remain, particularly in accurately placing and culturing multiple cell types, which is a key obstacle in organ printing. Despite these challenges, continued advancements in this field hold the potential to revolutionize both bone repair and organ engineering.^24^

These technologies offer a promising solution for bone repair, particularly in aging populations. As individuals age, the process of bone healing becomes more complex, making traditional scaffolds less effective. This highlights the critical need for smart biomaterials in bone repair. These biomaterials are biologically active, biosensitive, and autonomous, enabling them to directly facilitate bone healing. Such advancements hold great potential for improving bone regeneration, especially in the face of age‐related biological decline and conditions. However, further research is needed to evaluate the effectiveness of these technologies in older adults. Smart scaffolds and bioprinting technologies show significant potential for advancing bone repair in the elderly.^25^

In conclusion, scaffolds are essential for successful healing in TE. The combination of bioprinting technologies with smart scaffolds offers significant potential to transform bone TE and organ repair. Well‐designed scaffolds that ensure biocompatibility and mechanical stability have proven to support cell growth and tissue development. Additionally, these scaffolds contribute to advancements in TE and organ repair. As research progresses, the development of more advanced, functional, and biocompatible scaffolds will represent a significant step forward in TE applications.

### 3.2 Designing bioactive scaffolds for living tissues

Scaffold structures and their mechanical properties are crucial factors influencing cellular behavior; however, achieving full cellular differentiation requires the integration of bioactive components. To meet this need, bioactive scaffolds made from natural materials, such as ECM components, have been developed. These scaffolds are designed to release bioactive substances in a controlled manner, either through gradual degradation or by using vesicles and nanoparticles. For example, the inclusion of chitosan nanoparticles in electrodeposited scaffolds has been shown to enhance chondrogenic differentiation, while nanosilicates incorporated in copolymers promote osteogenic differentiation in human mesenchymal stem cells (hMSCs). Techniques like electrospinning, which generate ECM‐like nanofibers (NFs), have been proven to improve the scaffolds' bioactivity and support bone regeneration by adding nanopores. However, controlling the release of bioactive molecules remains complex due to the intricate regulation of growth factors. Supramolecular materials based on ureido‐pyrimidinone present a promising solution, as they can be functionalized with bioactive peptides to enhance cell adhesion. Moreover, self‐healing HGs, which mimic ECM dynamics, have shown great potential in supporting tissue remodeling while maintaining structural integrity. This highlights the significance of bioactive materials in improving both the mechanical and structural properties of scaffolds.^26^

The mechanical strength of scaffolds must be carefully optimized to withstand forces such as impact and compression at the implantation site. Ideally, the scaffold's mechanical properties should closely match those of natural bone to avoid complications like stress shielding from excessive force or structural failures and cracks from insufficient strength. Effective load transfer between the scaffold and regenerating tissue is essential for promoting optimal healing. Additionally, the incorporation of controlled porosity and well‐designed infill patterns within the scaffold structure has been shown to improve its stiffness and overall mechanical strength. AM technologies provide the opportunity to design customized scaffolds with precise architectures that replicate the natural porosity and connectivity of bone, allowing for solutions tailored to the needs of individual patients. However, the effectiveness of these advanced scaffolds in bone repair, especially for elderly patients, underscores the importance of integrating smart biomaterials to improve healing outcomes.^27^

Repairing significant bone defects in elderly individuals is a challenging task due to age‐related decline, chronic diseases, and infections. Existing synthetic scaffolds have limitations in facilitating effective bone regeneration. While smart multifunctional biomaterials have shown potential in supporting bone repair by interacting with cells and targeting specific sites, the exact mechanisms of action remain unclear. This study examines emerging smart technologies that may enhance bone repair in older adults, focusing on scaffolds with bioactive and bioreactive properties that may offer autonomous functions. Although these technologies show promising potential, further research is required, as many have not been evaluated in preclinical aging models. Therefore, the use of multifunctional smart scaffolds presents a promising strategy for improving bone repair in elderly patients, with the possibility of future applications offering significant benefits. Multifunctional scaffolds for bone repair following age‐related biological decline: Promising prospects for smart biomaterial‐driven technologies.^28^

In conclusion, the development of bioactive scaffolds is essential in TE, as they support cellular growth and tissue regeneration. By integrating bioactive components, these scaffolds replicate the natural ECM, allowing for the controlled release of growth factors. Advancements in technologies such as electrospinning and 3D printing are improving scaffold functionality by enabling the creation of tissue‐specific structures. As research in this area advances, bioactive scaffolds hold significant potential to enhance patient outcomes by aiding in the repair and regeneration of damaged tissues.

### 3.3 Innovative scaffold materials and applications

Innovative scaffold materials are essential components in TE. These include HG‐based bioactive scaffolds, electrospun fiber scaffolds, 3D printed scaffolds, metallic scaffolds with bioactive coatings, and natural polymer‐based scaffolds. Their use has grown significantly in various biomedical fields to facilitate the healing and regeneration of tissues such as bone, cartilage, and nerve cells. HG‐based bioactive scaffolds, TE aimed at replacing damaged or defective organs, is a complex process that seeks to restore biological functions and regenerate tissues. This is accomplished by utilizing safe scaffolds along with necessary bioactive substances and live cells. The success of this process depends on three critical factors: (i) the physicochemical properties of the scaffold, (ii) the integrity of the cells incorporated into the scaffold, and (iii) the biological environment into which the scaffold‐cell structure is implanted. In the fields of bone and cartilage TE, advanced biomimetic HGs present a promising solution, offering a 3D matrix with properties that support the embedded cells. These bioactive HGs are favored for their biocompatibility, high water absorption, mechanical strength, ability to house cells, incorporation of growth factors for tissue regeneration, and flexibility in production, all of which make them ideal for promoting tissue regeneration.^29^

**FIGURE 2:**
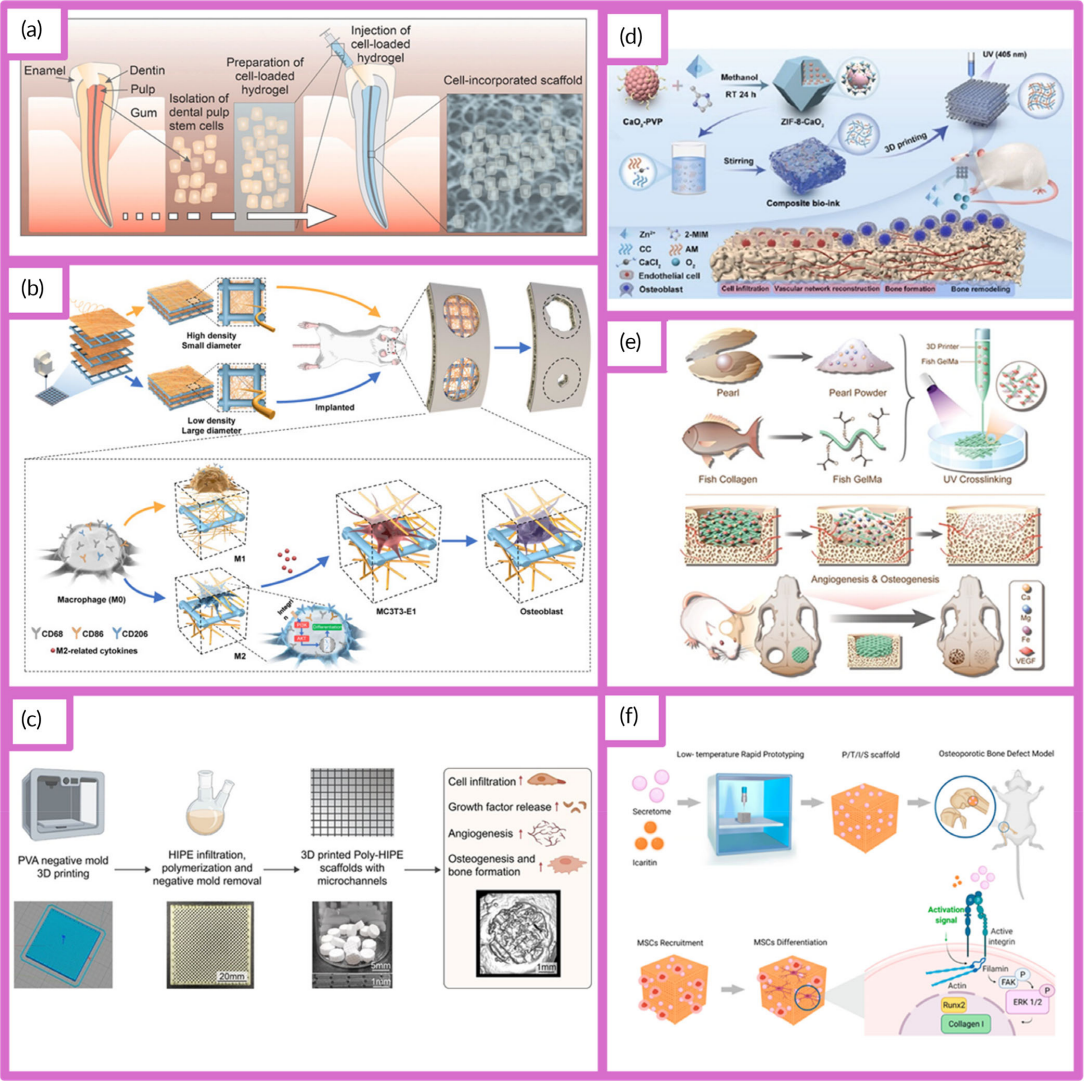
Schematic summaries of various advanced scaffold designs and fabrication strategies for tissue engineering and bone regeneration. (a) Schematic summary: the use of hydrogel scaffolds combined with stem cells as biocompatible carriers in dentin/pulp tissue engineering is demonstrated.^30^ (b) Schematic summary shows that three‐dimensional (3D)‐printed and electrospun microfibrous scaffolds modulate the immune response, increase angiogenesis, and accelerate bone regeneration by directing macrophages to the M2 phenotype.^31^ (c) Schematic outline demonstrates that polymerized High Internal Phase Emulsion (polyHIPE) scaffolds with microchannels and multiscale porosity can be fabricated using 3D‐printed polyvinyl alcohol (PVA) sacrificial molds. These scaffolds are shown to enhance vascularization and bone formation in vitro and in vivo by enabling the controlled release of BMP‐2, while enhancing cell infiltration, proliferation, and osteogenic differentiation.^32^ (d) Schematic summary demonstrates a 3D‐printed hybrid double‐network hydrogel scaffold with oxygen‐generating nanoparticles that provides sustained O_2_ release, customizable structure, and strong mechanics to promote vascularized bone regeneration without cells or growth factors.^33^ (e) Schematic summary shows the composition of pearl powder (PP) hybrid bioactive scaffolds fabricated by microfluidic 3D printing and their use in bone regeneration.^34^ (f) Schematic summary illustrates the preparation of P/T/I/S bioactive porous scaffolds, their use in healing osteoporotic bone defects, and the proposed mechanism of action.^35^ AM, additive manufacturing.

For example, HGs have been used as delivery systems for stem cells, such as human dental pulp stem cells (hDPSCs), to promote the regeneration of dentin and pulp tissue (Figure 2a).^30^

Electrospun fibrous scaffolds are produced using a technique that is ideal for creating cellular scaffolds, as it effectively replicates the structure of the ECM. These scaffolds offer multiple benefits, including a high surface area‐to‐volume ratio, excellent porosity, uniformity, flexibility in structure, and the ability to incorporate bioactive molecules for controlled release. By customizing these scaffolds with various materials, it is possible to create NF structures that closely resemble the ECM, facilitating nutrient and oxygen delivery to cells and allowing the incorporation of growth factors to support cell growth. These advancements make electrospun scaffolds highly valuable in regenerative medicine applications (Figure 2b).^36^ Although the electrospinning technique is highly effective in creating highly processable, ECM‐like structures, the biological interactions of scaffolds are complex and multidimensional. Understanding these interactions requires a focus on the capacity of scaffolds to modulate the immune response, as in the next study.

In this study, 3D‐printed PCL scaffolds were created using PLLA electrospun microfibrous (3D‐M‐EF) and nanofibrous (3D‐N‐EF) composites, with their physical structure carefully controlled via layer‐by‐layer deposition and electrospinning technologies. The study examined the immunomodulatory and osteogenic effects of these scaffolds. When comparing the 3D‐M‐EF and 3D‐N‐EF scaffolds, the 3D‐M‐EF scaffolds were found to have a stronger effect on polarizing RAW264.7 cells toward the M_2_ macrophage phenotype. This was evidenced by increased expression of M_2_ markers and decreased levels of M1 macrophage markers. The 3D‐M‐EF scaffolds facilitated M_2_ polarization through the PI_3_K/AKT (Phosphoinositide 3‐Kinase/Protein Kinase B) signaling pathway, as well as enhancing the expression of vascular endothelial growth factor (VEGF) and BMP‐2. Additionally, conditioned medium from RAW264.7 cells seeded on 3D‐M‐EF scaffolds promoted osteogenesis in MC_3_T_3_‐E_1_ cells. In vivo studies involving rat calvarial defect repair demonstrated that the 3D‐M‐EF scaffolds enhanced M_2_ macrophage polarization, supported angiogenesis, and accelerated bone formation. These results suggest that carefully designed 3D‐M‐EF scaffolds can regulate osteogenesis by modulating M_2_ macrophage polarization, highlighting the potential of 3D‐printed scaffolds to influence the osteoimmune environment and foster bone regeneration.^31^ This study is critical in that it highlights that the success of a scaffold is not only in harboring cells but can also actively promote healing by regulating the phenotype of immune cells (M2 polarization). This “osteoimmune” approach creates a new paradigm for bone regeneration. Similarly, the immunomodulatory capacity of scaffolds for other tissue types should not be underestimated.

In these other studies, aims to present in vitro tissue models for intestinal, skin, and pulmonary applications using electrospun membranes made from PCL combined with cellulose acetate (CA), cellulose acetate phthalate (CAP), ethylcellulose (EC), or methylcellulose (MC). The scaffolds were evaluated through various physicochemical characterizations and biocompatibility tests, using colorectal adenocarcinoma cells (for the intestine), keratinocytes and fibroblasts (for the skin), and bronchial and alveolar epithelial cells (for the lung). The scaffold compositions included NFs for PCL, PCL:CA, and PCL:EC, and a mix of micro‐ and NFs for PCL:CAP and PCL:MC. Water contact angle tests revealed that PCL, PCL:CA, PCL:CAP, and PCL:EC scaffolds were hydrophobic (with contact angles above 90°), while PCL:MC scaffolds exhibited hydrophilic characteristics. In the intestinal models, cells were able to adhere and proliferate on all scaffold types. In the skin models, PCL:CA and PCL:CAP combinations outperformed the other materials. For the lung models, 16HBE cells adhered and proliferated on PCL, PCL:CA, PCL:EC, and PCL:MC scaffolds, while A549 cells showed a comparable biological response on PCL, PCL:CA, and PCL:MC. The findings suggest that all the fibrous scaffolds were biocompatible with most of the tested cell types, indicating that PCL‐cellulose derivative mixtures hold promise as suitable materials for in vitro epithelial tissue modeling and toxicity screening.^37^ Electrospinning with different polymer blends shows that physicochemical properties such as hydrophobicity/hydrophilicity can be fine‐tuned. This tunability is critical for mimicking a specific epithelial tissue type (gut, skin, and lung) and offers valuable insight into how material selection in scaffold design can drive cellular responses. This fundamental principle applies not only to in vitro models but also to in vivo implants.

The creation of 3D‐printed bioactive scaffolds for bone defect repair is essential, as these scaffolds should facilitate vascularization and stimulate bone formation, making them highly suitable for biomedical applications. This study introduces a novel approach for fabricating polymerized High Internal Phase Emulsion (polyHIPE) scaffolds with microchannels and multiscale porosity using 3D‐printed, water‐soluble PVA sacrificial molds. Two types of sacrificial molds (250 and 500 μm) were created through FDM, filled with HIPE, and dissolved to form polyHIPE scaffolds with microchannels. In vitro experiments showed significant improvements in cell infiltration, proliferation, and osteogenic differentiation, highlighting the beneficial effects of the microchannels on cell behavior. Furthermore, the scaffolds demonstrated efficient loading and controlled release of the osteogenic factor BMP‐2, facilitated by microchannels. In vivo testing in a mouse model of critical‐size calvarial defects revealed improved vascularization and enhanced bone formation in polyHIPE scaffolds containing BMP‐2. This study presents an innovative method for creating scaffolds with multiscale porosity, highlighting their ability to enhance cell infiltration, control growth factor release, and improve overall in vivo performance. The findings suggest that these scaffolds hold great promise for applications in bone TE and regenerative medicine, with potential for clinical use (Figure 2c).^32^ This underscores a broader trend in scaffold research: the integration of structural innovations (e.g., multiscale porosity) with biochemical cues (e.g., growth factor delivery) to achieve synergistic effects in tissue regeneration.

This study introduces a self‐oxygenating 3D‐printed bioactive HG scaffold, which incorporates oxygen‐generating nanoparticles within a hybrid double‐network HG structure. This scaffold is designed to emulate the ECM and features a hybrid structure made of polyacrylamide and CaCl_2_‐cross‐linked sodium carboxymethylcellulose, providing both adequate compressive strength and 3D printability. The CaO_2_ nanoparticles, encapsulated within ZIF‐8, steadily release oxygen due to the well‐structured microporosity of ZIF‐8. This controlled oxygen release significantly enhances cell viability, proliferation, angiogenesis, and osteogenic differentiation, further supported by Zn^2+^ ions. The combination of oxygen release and the 3D‐printed pore structure helps prevent necrosis in the defect center, supporting cell infiltration by supplying essential nutrients and creating space. This promotes the growth of vascular networks and accelerates bone regeneration across the entire defect area in vivo. In conclusion, this study presents a novel approach for developing cell/factor‐free bone‐TE scaffolds, showing great promise for tissue regeneration and clinical applications (Figure 2d).^33^

In this study, a novel hybrid scaffold for bone regeneration is presented, combining pearl powder (PP) with the addition of VEGF to support tissue healing. The scaffold's composition and structure were precisely controlled using microfluidic‐assisted 3D printing technology, ensuring it met clinical needs. The incorporation of fish skin‐derived PP provided the scaffold with exceptional biocompatibility, enhanced cell adhesion, and promoted osteogenic differentiation. Additionally, the scaffold enabled the controlled release of VEGF, stimulating angiogenesis. The combined effects of osteogenesis and angiogenesis were shown to accelerate bone regeneration in a rat skull defect model, highlighting the potential of this PP hybrid scaffold for clinical bone regeneration applications (Figure 2e).^34^ The use of components of natural origin (PP) is an effective strategy to enhance bioactivity and osteoinduction. This approach combines the mechanical advantages of synthetic polymers with the superior biological recognition of natural materials. Similarly, the strategic addition of growth factors (VEGF) emphasizes an integrated approach, targeting the synergy between osteogenesis and angiogenesis, essential for effective bone regeneration.

In this study, a novel approach is introduced for fabricating 3D‐printed poly(glycerol sebacate) (PGS)‐chondroitin sulfate/Gel scaffolds, utilizing a technique based on super‐swelling absorption and cross‐linked network locking. The method employs U.S. Food and Drug Administration (FDA)‐approved materials, including PGS, gelatin (Gel), and chondroitin sulfate. The resulting scaffolds exhibit desirable properties, such as well‐structured hierarchical designs, excellent elasticity, enhanced hydrophilicity, and bioactivity that promote cartilage regeneration. These characteristics support the adhesion, proliferation, and migration of chondrocytes. A significant advantage of this approach is the scaffold's degradation rate, which aligns with the regeneration of cartilage, ensuring complete resorption as mature tissue forms. In vivo testing in a rabbit trochlear groove defect model demonstrates the scaffold's effective cartilage repair capabilities, highlighting its potential for clinical applications in cartilage regeneration.^38^ Cartilage regeneration presents a different set of material requirements than bone; flexibility, bioactivity, and a compatible degradation rate are critical. This study presents a hybrid system of FDA‐approved materials specifically designed to meet these needs. This strongly supports the principle that scaffold design should be target tissue specific.

In another study, a poly(lactic‐co‐glycolic acid)/β‐calcium phosphate (PLGA/TCP)‐based scaffold was developed using a 3D printing technique, incorporating icaritin (ICT) and the secretome, a bioactive phytomolecule derived from human fetal mesenchymal stem cells (HFS). This composite scaffold (CS) provides both mechanical stability and biological cues to promote bone defect healing. The sustained release of ICT and HFS from the scaffold enhances the migration of MSCs and supports bone regeneration at femoral defect sites in an ovariectomy (OVX)‐induced osteoporotic rat model. Mechanistic investigations indicate that the combined action of ICT and HFS activates the integrin–FAK (focal adhesion kinase)–extracellular signal‐regulated kinase 1/2 (ERK1/2)–Runt‐related transcription factor 2 (Runx2) signaling pathway, which likely aids in MSC recruitment to the scaffold and stimulates osteogenesis. In conclusion, the PLGA/TCP/ICT/HFS (P/T/I/S) bioactive scaffold shows strong potential for repairing osteoporotic bone defects and holds promising clinical applications (Figure 2f).^35^ The controlled release of pharmacoactive molecules (icaritin) and the cell secretome pharmacologically directs the regenerative process, turning scaffolds into a localized and continuous therapy platform. This study demonstrates that scaffolds can not only provide mechanical support but also actively direct cell behavior and tissue formation by activating complex molecular signaling pathways (integrin–FAK‐ERK).

Metallic scaffolds with bioactive coating, to fix the bone in orthopedics, it is almost always necessary to use implants. Metals are commonly used in these applications due to their robust mechanical properties and ability to bear loads. However, despite their widespread use in replacing hard tissue, metallic implants face several issues, particularly concerning their biocompatibility. These issues can include excessive wear, poor corrosion resistance, increased risk of infections, and stress shielding. To address these problems, various coating techniques have been developed to improve the performance of metallic implants both in vitro and in vivo. By combining metals with bioceramic or polymer coatings, it is possible to create implants with bioactive, osteogenic, antibacterial, or biodegradable properties, providing multifaceted solutions to these challenges.^39^

Recent progress in materials science has led to the creation of novel natural polymer‐based scaffolds that replicate the natural characteristics of bone, aiding in the repair of bone defects. TE, a multidisciplinary field that merges life sciences with engineering, aims to develop biological substitutes that can restore or replace tissue function, or even whole organs. Despite the potential of bone grafting, several challenges persist, such as complications at the donor site, risks of immune rejection or infection (in the case of allogeneic grafts), and the limited availability of donor bone to meet the increasing demand. The development of synthetic bone implant materials is critical for bone tissue regeneration, as these materials provide essential structural support without causing damage to surrounding biological tissue. Biodegradable matrices are particularly important, as they function as temporary scaffolds that facilitate the growth and regeneration of bone tissue. Typically, thermoplastic aliphatic polyesters are used in the fabrication of these scaffolds, promoting cell growth in a 3D structure that can be implanted into damaged bone or tissue areas. The incorporation of natural sources in the development of these materials plays a significant role in advancing TE, either directly or indirectly.^40^ This provides an overview of how synthetic and natural polymers can be combined to create biologically mimicked, biodegradable scaffolds to overcome the limitations of autologous grafts and enable personalized therapies. The main goal is to provide a 3D microenvironment where cells can attach, grow, and form functional new tissue.

This study introduces a microfluidic method utilizing on‐chip hydrodynamic flow focusing for the synthesis of alginate nanogels encapsulating transforming growth factor beta 3 (TGF‐β3) through an ionic gelation technique. The goal was to achieve precise release profiles of these bioactive agents during the chondrogenic differentiation of MSCs. By carefully modulating the flow rate ratio (FRR) in a microfluidic device with cross‐junction microchannels, alginate nanogels of various sizes were successfully produced. The results highlight the potential of this approach as an effective method for creating bioactive‐loaded polymeric nanogels, with significant applications in drug delivery and TE.^41^

Despite significant advancements in the design and functionality of bioactive scaffolds, several critical challenges remain unresolved. The integration of bioactive cues into scaffolds often faces difficulties in achieving precise, sustained, and reproducible release profiles of growth factors, which are crucial for orchestrating complex regenerative processes. Many studies demonstrate success in small animal models, yet translation into large‐animal and clinical settings has been inconsistent, reflecting a gap between experimental conditions and physiological complexity in humans. Furthermore, techniques such as electrospinning and AM enable precise structural control; scalability and cost‐effectiveness for clinical‐grade scaffold production remain major barriers. Another unresolved issue lies in balancing mechanical stability with biodegradability: scaffolds that degrade too quickly risk structural failure, while those that persist too long may impede tissue remodeling or trigger chronic inflammation. Similarly, multifunctional “smart” scaffolds incorporating nanoparticles, bioactive peptides, or oxygen‐releasing systems show promise, but concerns about long‐term biosafety, immune compatibility, and regulatory approval remain largely unaddressed. Metallic scaffolds, although mechanically robust, still struggle with issues such as corrosion, stress shielding, and integration with soft tissue interfaces. Moreover, while natural polymers improve biocompatibility, their mechanical weakness and batch‐to‐batch variability limit reproducibility, whereas synthetic polymers offer greater control at the expense of reduced bioactivity. These trade‐offs highlight the urgent need for hybrid scaffold systems that combine the strengths of different material classes. Finally, comparative studies between scaffold types remain scarce, making it difficult to establish standardized criteria for clinical application. Without harmonized testing protocols, reproducibility across laboratories remains limited, slowing regulatory acceptance and translation to real‐world therapeutic use.

## 4 BIOPRINTING IN SKIN AND TRANSDERMAL SYSTEMS: INNOVATIVE APPROACHES AND APPLICATIONS

### 4.1 Skin production: innovative solutions with bioprinting

Bioprinting has emerged as a groundbreaking technology with transformative potential across several areas, such as the creation of full‐thickness skin (FTSs), the development of bioinks, wound and burn management, MN system applications, and drug delivery. This technology enables the development of advanced skin production techniques, making treatment options more effective and tailored to individual needs. Full thickness, FTSs consists of the complete epidermis and dermis.^42^ In this study, a novel bioink was developed, combining gelatin, sodium alginate, and fibrinogen. By optimizing the bioink components, 3D model design, and printing parameters, a dermal layer with embedded fibroblasts was successfully fabricated. Laminin and keratinocytes were then added to the epidermal layer. FTSs tissue was created using an air–liquid interface (ALI) culture, supported by sterile wire mesh. Histological analysis and immunofluorescence staining revealed that the bioprinted skin closely mimicked human skin, showing markers of epidermal differentiation and stratum corneum formation. This study offers an effective and efficient method for fabricating FTSs constructs, providing valuable contributions to both the academic study and practical development of artificial skin.^43^

In this study, another report describes the development of a 3D bioprinted human cell‐based FTSs model that closely mimics the structural, mechanical, and biochemical characteristics of human skin. The epidermis–dermis junction's unique undulated architecture was successfully recreated in the 3D bioprinted skin equivalent. The migration of keratinocytes within the construct, combined with differentiation events, resulted in the re‐epithelialization of the tissue. Cell–cell interactions and diffusible factors stimulated the expression of differentiation and cornification markers in a region‐specific manner. The bioink's architectural properties and the silk‐based microenvironment promoted the deposition of basement membrane proteins at the interface. Notably, comprehensive transcriptomics and proteomics analyses highlighted significant similarities between the 3D bioprinted FTSs model and native human skin, revealing the involvement of various pathways related to skin development and physiology. These include processes such as skin development, ECM organization, keratinization, cornification, and collagen fibril organization. The potential of such bioprinted in vitro skin models for drug and cosmetic product testing, as well as for advancing our understanding of human skin's complex physiological processes, is substantial. This approach could help bridge the gap between traditional monolayer cultures, 3D models, and animal testing.^44^ This study reveals how realistically bioprinting can mimic the complex, multilayered structure and physiology of human skin, a revolutionary step toward creating in vitro models of the “living organs” highlighted in the title.

This study presents the development of a full‐thickness biomimetic skin equivalent created using extrusion‐based bioprinting. The 3D bioprinted skin model features a cellular collagen dermal layer supported by an acellular PCL/collagen scaffold, with bioprinted keratinocytes sequentially layered on top. The construct undergoes airlifting to promote stratification and differentiation. The performance of the bioprinted skin constructs is compared to that of full‐thickness human skin models created by manually seeding cells, evaluating factors such as cell proliferation, viability, histology, immunostaining, and barrier function to assess both qualitative and quantitative differences. The findings highlight the potential for refining specific 3D bioprinting techniques to produce full‐thickness reconstructed human skin in a reproducible, consistent, and potentially scalable manner.^45^ Taken together, these two studies show that one of the major advantages of bioprinting over standard, hand‐generated models is scalability and reproducibility. This increases the potential for bioprinted skin models to become a reliable and widespread platform for drug testing.

Recent advancements in the field of wound care have led to the development of innovative functional wound dressings, particularly those made from HGs or enhanced with growth factors, stem cells, antibiotics, and other therapeutic agents to aid and accelerate the healing process. These breakthroughs, powered by cutting‐edge technologies, have enabled the creation of highly customized dressings designed to meet the unique needs of individual wounds, optimizing the healing environment. These advanced dressings incorporate a variety of biological elements, such as antibacterial agents, pH regulators, moisture, oxygen, nutrients, and cellular components like calcium, hydrogen peroxide, copper, and iron. Some dressings are also equipped with exudate drainage regulators, allowing them to address wounds of varying severity. The impact of these innovative dressings on wound healing includes enhanced closure rates, re‐epithelialization, and angiogenesis, while demonstrating no signs of cellular toxicity. An essential aspect of this progress is the selection of production techniques, which play a crucial role in achieving precision and speed in the creation of personalized HGs, particularly when working with complex wound shapes. Traditional methods, such as molding and casting, often fail to produce the high‐resolution, intricate designs required for certain wounds. In contrast, 3D bioprinting has revolutionized wound care by allowing for the fabrication of anatomically accurate, patient‐specific dressings that incorporate not only antibacterial agents but also bioactive compounds. 3D bioprinting, as an AM process, leverages various printing techniques, including SLA, DIW, and DLP, in combination with CAD to rapidly create highly detailed and customized structures. This process allows for the precise creation of models, using 3D CAD software or cross‐sectional images obtained from CT or magnetic resonance imaging (MRI) scans. The primary advantage of 3D bioprinting lies in its ability to fabricate materials that closely mimic the specific characteristics of the wound site, integrating living cells, therapeutic agents, and HGs with remarkable accuracy and resolution.^46^ The potential of bioprinting is not limited to in vitro models; it extends to the production of personalized, bioactive wound dressings that can be applied directly to patient wounds. The revolution here is that, unlike traditional casting methods, CAD‐based 3D printing enables the design of scaffolds that can contain drugs or cells that precisely conform to complex wound geometries.

MN system, MNs are tiny, micron‐scale devices used for the transdermal delivery of pharmaceutical agents, offering a minimally invasive and painless alternative to traditional methods. Over the past decade, various AM technologies have been explored to fabricate these MNs. However, these technologies face challenges, including material compatibility, bioavailability concerns, and the high costs and lengthy production times associated with the process. AM, commonly known as 3D printing, is a groundbreaking technology that allows for the creation of 3D solid objects by building them layer by layer.^47^ Another critical transdermal application of bioprinting technology is MN systems. The challenges of their traditional manufacturing, the need for personalized dosing, and rapid prototyping make them an ideal candidate for AM.

With the emergence of 3D bioprinting technology, drug delivery to the skin has enabled the creation of customized drug delivery systems, thereby improving treatment effectiveness by allowing the direct application of active pharmaceutical compounds to the skin. This approach allows for more precise and efficient targeting of specific areas, promoting faster therapeutic outcomes. The incorporation of 3D printing with nanomaterials introduces a variety of adaptable properties for pharmaceutical use, though few studies have explored its application in skin delivery systems.

In this study, the hydrophobic drug clobetasol propionate (CP) was encapsulated in mesoporous silica nanoparticles (MSN) at a 3:1 (w/w) ratio to produce novel bioadhesive, hydrophilic skin delivery films. These films were fabricated using a 3D‐printable ink composed of pectin (5% w/v) and carboxymethylcellulose (5% w/v). Encapsulating CP in MSN led to a decrease in its crystallinity and an increase in its dissolution efficiency after 72 h (65.70 ± 6.52%), compared to CP in its dispersed form (40.79 ± 4.75%). This improvement is attributed to the partial conversion of CP into an amorphous state. The CP‐loaded MSN was incorporated into the 3D‐printable ink, which exhibited high tensile strength (3.613 ± 0.38 N), consistent drug dosage (0.48 ± 0.032 mg/g per film), and full drug release within 10 h. Additionally, the inclusion of pectin in the ink improved the skin adhesion properties of the films, resulting in an adhesion work of 782 ± 105 mN mm. Thus, the combination of MSN and the innovative printable ink, composed of carboxymethylcellulose and pectin, presents a new platform for producing 3D‐printed bioadhesive films, marking a significant step forward in the development of advanced skin delivery systems.^48^

### 4.2 Transdermal systems: technologies beyond the skin

Transdermal drug delivery systems (TDDS) offer a promising solution for the non‐invasive and controlled release of pharmaceuticals through the skin. However, the skin's natural barrier remains a significant challenge to the efficient absorption of drugs.^49^ To address this issue, emerging technologies such as MN systems, nanotechnology, and 3D bioprinting are showing great potential in enhancing transdermal systems. Bioinks, particularly those made from HGs and polymers, are being utilized to improve both skin engineering and drug delivery. In addition, natural materials and biomimetic approaches are being explored to replicate the skin's structure to optimize drug release and therapeutic efficacy. These innovations are set to improve the efficiency of transdermal delivery systems and pave the way for personalized treatment solutions. One of the most significant advancements in TDDS is the development of MN technology. These MNs, designed for the delivery of various pharmaceutical agents, overcome the limitations of traditional transdermal and oral drug delivery methods. MNs are increasingly popular among patients due to their ease of self‐administration, minimal invasiveness, and lack of pain compared to conventional injection methods. Over time, various improvements have been made to MNs, making them more cost‐effective, precise, and versatile for diverse applications. A key innovation in this field is the adoption of 3D printing technology for MN fabrication. Advancements in 3D printing have led to improved fabrication precision, higher resolution, and the use of low‐cost raw materials. This review will examine the different types of MNs, fabrication techniques, materials involved in their construction, and the latest applications of 3D‐printed MNs.^50^

In this study, a design for a transdermal patch featuring 25 MNs is presented, fabricated using 3D printing through SLA with a biocompatible Class 1 resin and a 0° printing angle. The mechanical behavior of the MN array was evaluated using finite element analysis (FEA) with ANSYS software. The analysis revealed key results, including a Von Mises stress of 18.057 MPa, a maximum deformation of 2.179 × 10^−3^, and a safety factor of 4. Additionally, a flow simulation indicated that a pressure of 1.084 Pa and a fluid velocity of 4.800 m/s were required to achieve a volumetric flow rate of 4.447 × 10–5 cm^3^/s. The findings from this study provide essential insights that will guide the development and optimization of future TDDS.^51^ This study demonstrates how SLA and FEA provide a basis for virtually optimizing MN design in terms of mechanical strength and drug flow profiles.

#### 4.2.1 Nanotechnology in bioprinting‐assisted skin and transdermal systems

The integration of nanotechnology with 3D bioprinting has significantly improved the effectiveness of TDDS. This study explores the development of a smart biologically active polymeric HG transdermal nanomaterial, which combines gelatin and sodium alginate modified with humic acids. The results show that incorporating these novel HG materials enhances the skin's moisture‐lipid balance. The fabrication of these smart transdermal patches was achieved using a micro molding technique, with an FDM 3D printer employed to create the master mold. The patch designs were developed using Autodesk's Fusion 360 software. The resulting 3D model was processed by a slicer to generate the necessary instructions for the 3D printer. The 3D‐printed smart HG transdermal materials, based on gelatin, sodium alginate, and modified by humic acids, exhibit favorable properties for effective transdermal drug delivery.^52^ This study goes one step further by highlighting the intersection of nanotechnology and bioscaffolding (using FDM for micromolding). Modification with humic acids gave the bioactive scaffolds a smart function, optimizing the interaction with the skin, which embodies the emphasis on “bioactive” in the title. This is where materials science and biology come together.

Recent advancements have seen the incorporation of bioinks based on hydrogels (HGs) and polymers to improve skin engineering and drug delivery systems. This study combines 3D bioprinting and electrospinning techniques to develop a novel double‐layered transdermal drug delivery patch (TDDP) for the treatment of rheumatoid arthritis (RA). The first layer was created using 3D printing of a new HG containing hyaluronic acid (HA), which helps maintain joint integrity, alongside dexamethasone (DEX), an anti‐inflammatory drug. The second layer consisted of electrospun PCL nanofibers (NFs) loaded with naringin (NAR), a natural antimicrobial and anti‐inflammatory compound. The morphology of the NFs was examined using scanning electron microscopy (SEM), revealing a diameter range of 156.28 to 220.66 nm. The physicochemical properties of the developed TDDP were analyzed using Fourier‐transform infrared (FTIR) spectroscopy and differential scanning calorimetry (DSC). The DEX‐loaded HG exhibited a sustained release, delivering approximately 98% of the drug over 10 days. In vivo studies confirmed the effectiveness of the TDDP, showing a significant reduction in the levels of proinflammatory cytokines (IL‐6 and TNF‐α) in plasma samples of *Rattus norvegicus*, as measured by sandwich enzyme‐linked immunosorbent assay (ELISA). Additionally, histological analysis of the ankle joints revealed a decrease in cell infiltration and preservation of joint tissue structure, confirming the alleviation of RA symptoms.^53^

In this study, the effects of different proportions of two liquid monomers PEGDA and vinyl pyrrolidone (VP), on several key properties were examined, including mechanical strength, polymerization rate, swelling rate, 3D printing resolution, and the safety profile of the resulting polymer. The ideal resin formulation was identified as a 7:3 weight ratio of VP to PEGDA. The study showed that AHP‐3, the compound used, remained stable throughout the fabrication process and did not alter the final polymer's physical properties. A personalized MN patch was then fabricated using CAD software and a DLP 3D printer with the optimal resin. In vitro tests confirmed that the MN patch was capable of penetrating human cadaver skin and retained its structural integrity after compression. Additionally, the final polymer displayed minimal toxicity toward human dermal fibroblasts. These results indicate that the personalized MN patch, produced using this photopolymer, could be an effective approach for improving the transdermal delivery of AHP‐3, showing promise for wrinkle management.^54^ The personalized MN patch addresses a critical aspect for the clinical translation of the bioprinting revolution: personalization. Using CAD software and DLP printing, structures optimized for patient‐specific needs can be produced. This is at the heart of the concept of “innovative design” and represents a paradigm shift from one‐size‐fits‐all therapies to personalized and more effective therapies.

3D printing for transdermal biomimetics and natural materials, natural organisms have evolved a range of functional biomaterials and structures to adapt to the challenges of their environments. These structures exhibit exceptional properties such as superhydrophobicity, anisotropy, and improved mechanical strength, which have inspired the development of advanced multifunctional devices. However, the scarcity of suitable materials and the limitations of traditional manufacturing methods for producing complex, multiscale structures have slowed progress in bio‐inspired design and fabrication. AM, or 3D printing, has emerged as a groundbreaking technology, offering significant design flexibility and enabling the creation of intricate, multiscale, hierarchical, and multi‐material structures. This paper presents an extensive review of the current advancements in 3D printing for surface and interface structures, focusing on the materials, designs, and functional applications involved. The review also explores various bio‐inspired surface structures produced through 3D printing, classifying them according to their specific properties and applications. It is highlighted that some of these properties can be applied across multiple fields. The optimized designs of these bio‐inspired 3D‐printed surfaces present great potential for cost‐effective, efficient, and high‐performance applications. Finally, the paper discusses the challenges and opportunities in the development of functional surfaces and interfaces, emphasizing the need for more versatile materials, refined structural designs, and improved cost‐effectiveness.^55^

### 4.3 Clinical applications and future perspectives

**FIGURE 3:**
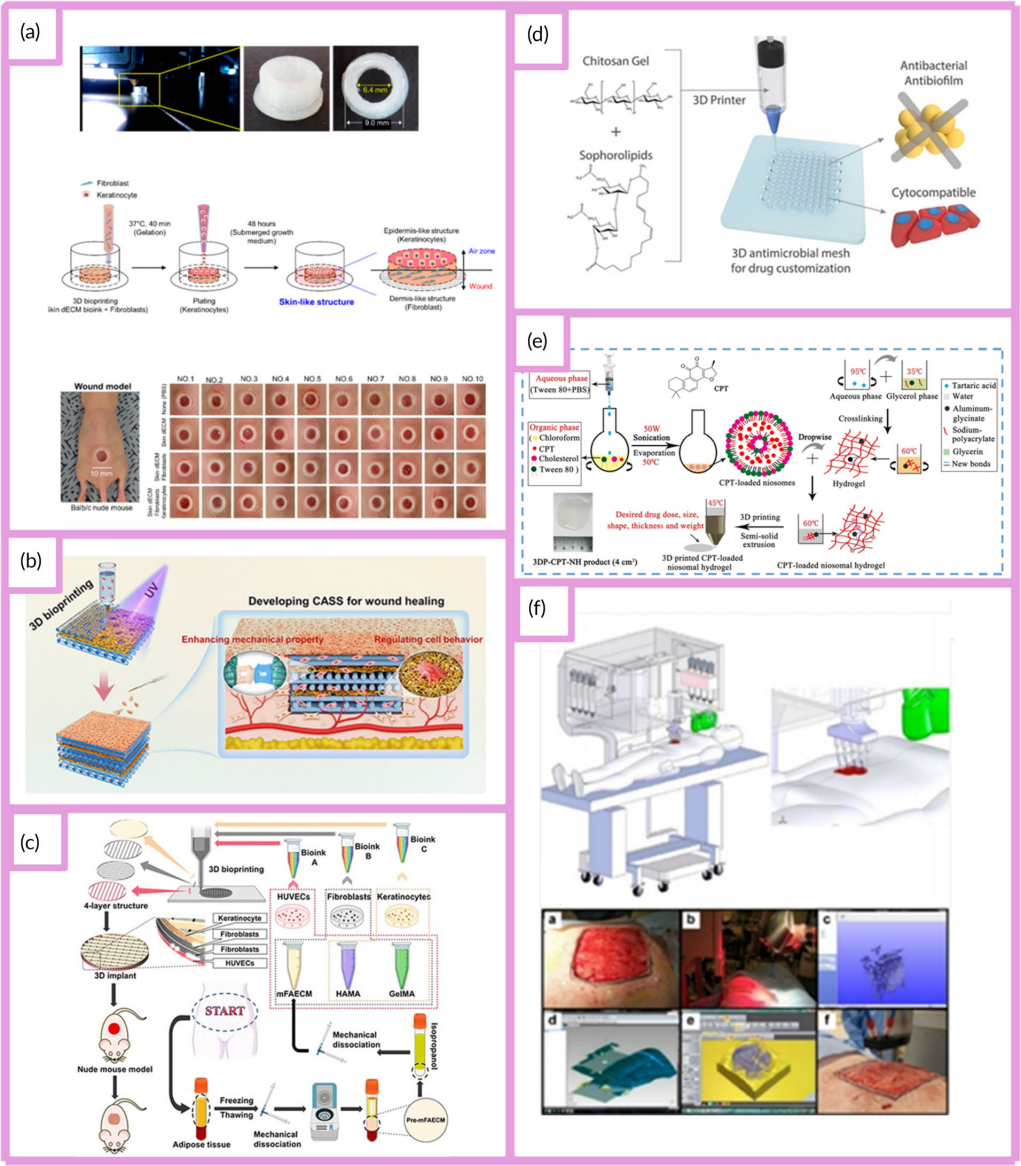
Schematic summaries of various 3D‐printed hydrogel and bioink based strategies for skin tissue engineering, wound healing, and drung delivery. (a) Schematic summary shows the fabrication of chimney structures and skin substitutes by three‐dimensional (3D) printing and the verification of a uniform chimney model across all experimental groups.^57^ (b) Schematic abstract demonstrates that the composite artificial skin substitute (CASS) composite hydrogel printing architecture integrated with patterned nanofibrous films inspired by collagen nanofibers can accelerate the regeneration of large‐area skin defects by creating mechanically durable and cell behavior‐guiding artificial dermis and epidermis.^58^ (c) Schematic abstract demonstrates that developing biocompatible, printable, and biomimetic multilayer 3D bioprinted skin implants using microfractured adipose extracellular matrix (mFAECM) can accelerate the repair of full‐thickness skin defects.^59^ (d) Schematic summary demonstrates that chitosan‐based 3D‐printed hydrogel coatings enriched with antimicrobial sophorolipids exhibited high antibacterial and antibiofilm activity against Staphylococcus aureus and were biocompatible toward human dermal fibroblasts, and could be used for customizable bioactive medical device coatings.^60^ (e) Schematic summary demonstrates that cryptotanshinone‐loaded 3D‐printed niosomal hydrogels (3DP‐CPT‐NH) are a drug delivery system for personalized topical acne treatments with high transdermal penetration, effective anti‐acne activity, and low skin irritation^61^. (f) The schematic summary shows the scanning, design, and printing steps of the skin bioprinter with an integrated wound scanning and material spraying system.^62^ dECM, decellularized extracellular matrix; GelMA, gelatin methacryloyl; HAMA, methacrylate hyaluronic acid.

Bioprinting technology presents significant potential for advancing personalized therapies through innovative approaches in skin regeneration and transdermal drug delivery. With applications ranging from cellular TE to MN systems, this technology shows promise in enhancing skin wound healing and enabling precise drug delivery. This review highlights the clinical applications, existing challenges, and prospects of bioprinting. For example, in this study, a skin‐like structure was created by combining skin‐derived decellularized extracellular matrix (dECM), bioink, keratinocytes, and fibroblasts^56^ using 3D printing technology. The therapeutic effectiveness of the fabricated skin substitutes was assessed using a chimney model that simulates the human wound‐healing process. The results showed that the 3D‐printed skin promoted faster re‐epithelialization and improved tissue regeneration compared to the control group. These findings hold promise for advancing technologies that enable the rapid and customizable production of skin replacement tissues, potentially benefiting patients requiring skin grafts (Figure 3a).^57^

In a similar study, a patterned nanofibrous film was integrated into a printed HG scaffold to produce a composite artificial skin substitute (CASS), aimed at mimicking the stress‐bearing and regulatory functions of collagen fibers. The artificial dermis was constructed using a hybrid gelatin‐hyaluronan HG containing human dermal fibroblasts, featuring gradient porosity, and incorporated with patterned nanofibrous films. The artificial epidermis was created by seeding human keratinocytes onto the dermal layer. The collagen‐like nanofibrous film significantly improved the tensile strength and fracture resistance of the CASS, making it suitable for stable implantation into skin defects. Additionally, the patterned film provided biological signals that guided cell behavior. As a result, CASS effectively promoted the regeneration of large skin defects in both mice and pig models by encouraging re‐epithelialization and collagen deposition. This method represents a promising strategy for developing composite bioprinted structures that enhance mechanical properties and regulate cellular responses, positioning CASS as a viable solution for treating extensive skin injuries (Figure 3b).^58^

This study introduces a novel method for processing adipose tissue into microfragmented adipose extracellular matrix (mFAECM), which serves as a key component of bioink for the fabrication of 3D‐bioprinted, biomimetic multilayer implants. The mFAECM retains vital components such as collagen and sulfated glycosaminoglycans, mimicking the properties of native tissue. In vitro, the mFAECM composite demonstrated strong biocompatibility, printability, and structural integrity, facilitating effective cell adhesion. In a FTSs defect model using nude mice, cells within the implant survived and actively participated in the wound healing process. The implant retained its structure throughout the healing phase, undergoing gradual metabolic changes. The application of mFAECM‐based bioinks in creating multilayer implants holds significant potential for accelerating wound healing by supporting tissue contraction, collagen production, remodeling, and neovascularization. This study presents an innovative approach for fabricating 3D‐bioprinted skin substitutes, offering a promising solution for treating FTSs injuries (Figure 3c).^59^

In this study, the intradermal (ID) space is investigated as a promising area for minimally invasive drug delivery and diagnostic applications. MNs, MNs patches, and microarray patches (MAPs) consist of tiny projections designed to painlessly penetrate the skin, providing access to the epidermal and dermal layers. While these devices show considerable potential, their development has been limited by outdated manufacturing techniques, such as lithography and molding, which are difficult to scale and restrict innovation in MAP designs. To overcome these challenges, the DeSimone Laboratory has introduced a high‐resolution continuous liquid interface production (CLIP) 3D printing technology. This technique uses light and oxygen to create a continuous polymerization dead zone at the build surface, enabling the rapid, precise creation of MAPs with customizable geometries. This advanced method has led to the creation of new types of lattice MAPs (L‐MAPs) and dynamic MAPs (D‐MAPs), which can deliver both solid and liquid cargos, as well as extract interstitial fluid. This study aims to explore how AM can transform the development of MAPs, opening up new opportunities for minimally invasive drug delivery and diagnostic systems.^63^

In this different study, a 3D‐printed antimicrobial chitosan‐biosurfactant HG mesh was created to coat polydimethylsiloxane‐based medical devices, aiming to prevent infections. The 3D structure featured porosity, which allowed for the incorporation of tailored bioactive components. Two biosurfactants, surfactin and sophorolipids, were biosynthesized and assessed for their antimicrobial effects. The process for printing surfactant‐chitosan‐based coatings was optimized, and the resulting 3D structures were analyzed for properties like wettability, antimicrobial activity, FTIR‐ATR spectra, and biocompatibility. Among the biosurfactants tested, sophorolipids exhibited superior antibacterial activity, especially against Gram‐positive bacteria, and were chosen for the production of the chitosan‐based 3D coatings. These SL‐impregnated coatings showed the most effective antibacterial action against *Staphylococcus aureus* planktonic bacteria (with 61% growth inhibition) and demonstrated strong antibiofilm activity (a reduction of 2 log units) compared to the control. Regarding biocompatibility, the coatings were found to be compatible with human dermal fibroblasts. Additionally, the mesh structure was found to be suitable for the incorporation of bioactive compounds, such as HA, indicating its potential for customized therapeutic applications (Figure 3d).^60^ This study points to another critical application area of bioprinting, namely the fight against infection. The production of personalized coatings equipped with antimicrobial bioactive components demonstrates the potential of bioprinting to transform medical devices from passive implants into active therapeutic systems.

In this study, we developed a 3D‐printed niosomal hydrogel (3DP‐NH) loaded with cryptotanshinone (CPT) for use as a targeted acne treatment. CPT‐loaded niosomes were created using the reverse phase evaporation method, and the formulation was optimized through response surface methodology. Analysis revealed that the optimized niosomes had a size below 150 nm and exhibited an entrapment efficiency between 67% and 71%. These niosomes were integrated into a HG by a dropwise addition technique, resulting in a CPT‐loaded niosomal hydrogel (CPT‐NH). The HG was subsequently fabricated into a 3D‐printed form using an extrusion‐based printer, ensuring precise control over drug concentration, shape, and size. In vitro drug release testing indicated that 3DP‐CPT‐NH adhered to the Korsmeyer‐Peppas release model. Experiments evaluating permeation and deposition demonstrated that 3DP‐CPT‐NH achieved significantly higher transdermal flux, increased Q24 values, and greater CPT deposition (*p* < 0.05) compared to 3D‐printed HGs without niosomes (3DP‐CPT‐CH). In vivo tests conducted on an acne rat model showed that 3DP‐CPT‐NH had superior anti‐acne effectiveness without causing skin irritation. The enhanced delivery and activity of CPT were attributed to improved skin hydration, larger inter‐corneocyte gaps in the stratum corneum, and disrupted lipid organization. These findings underscore the promise of 3DP‐CPT‐NH as a novel, customized topical drug delivery system for treating acne effectively (Figure 3e).^61^ The integration of niosomes is an excellent example of how nanotechnology can be combined with bioprinting to improve the efficiency of bioprinted drug delivery systems. This proves that “Bioactive Scaffolds” can also play an active role in pharmaceutical applications, providing not only structural support but also targeted, controlled therapies.

In this another study, a bilayer drug‐loaded skin scaffold was developed for the repair of FTSs defects. The outer layer, designed to replicate the epidermis, was fabricated by electrospinning PCL NFs loaded with amoxicillin (AMX), creating an antibacterial NF membrane (PCL‐AMX). The inner layer, mimicking the dermis, was constructed using 3D printing to embed human epidermal growth factor (rhEGF) within a sodium alginate‐gelatin hydrogel (SG‐rhEGF). This combination aimed to maintain wound hydration and facilitate healing. Successful incorporation of both AMX and rhEGF was achieved. The scaffold demonstrated excellent physicochemical properties, with an elongation at break of 102.09 ± 6.74% and a tensile modulus of 206.83 ± 32.10 kPa. The outer layer exhibited hydrophobicity (WCA = 112.09 ± 4.67°), while the inner layer was hydrophilic (WCA = 48.87 ± 5.52°). The scaffold also displayed effective drug release and antibacterial properties. In vitro and in vivo assessments confirmed that the scaffold promoted cell adhesion, proliferation, and skin wound healing, demonstrating excellent biocompatibility. These findings underscore the scaffold's promising potential for clinical applications in skin regeneration.^64^ The success of a double‐layered scaffold demonstrates the greatest power of bioprinting: combining multiple materials and bioactive factors with different functions in a single manufacturing process to create anatomically correct and functionally integrated structures.

In a similar study, the use of 3D printing technologies to fabricate flexible, personalized anti‐acne drug delivery devices was investigated. Two different 3D printing methods, FDM and SLA, were evaluated. To ensure a customized fit, 3D scanning technology was utilized to generate a precise anatomical model of a person's nose. For the FDM method, commercially available filaments, including Flex EcoPLA™ (FPLA) and PCL, were infused with salicylic acid using hot melt extrusion (HME), achieving a theoretical drug loading of 2% w/w. These filaments served as the feedstock material for the 3D printing process. Analysis revealed that the actual drug loading was 0.4% w/w for FPLA‐salicylic acid and 1.2% w/w for PCL‐salicylic acid, indicating thermal degradation of the drug during HME and printing. Drug release studies using Franz cells with a synthetic membrane showed that the FDM‐printed samples released less than 187 μg/cm^2^ within 3 h. While the FPLA‐salicylic acid filament was successfully printed into a nose‐shaped mask, the PCL‐salicylic acid filament failed to produce a functional print. In the SLA method, salicylic acid was dissolved in mixtures of PEGDA and PEG and cured using a laser beam. SLA‐produced devices demonstrated higher resolution and drug loading (1.9% w/w) compared to FDM, with no evidence of drug degradation. Drug release tests showed that the SLA‐printed devices released 229 and 291 μg/cm^2^ within 3 h, surpassing the diffusion rates of the FDM‐printed devices. In conclusion, SLA was identified as the more effective 3D printing technique for fabricating salicylic acid‐based anti‐acne devices. The integration of 3D scanning and printing technologies highlights the potential for creating personalized drug delivery systems tailored to individual anatomical features.^65^

In this study, an innovative microfabrication method is introduced for creating MNs using FDM 3D printing with PLA. PLA, a thermoplastic material, is recognized for its renewability, biodegradability, and FDA approval for medical use. The study illustrates how PLA's natural degradability can effectively overcome the low‐resolution limitation associated with FDM 3D printing. To improve precision, a post‐processing chemical etching technique was developed, which significantly enhanced the detail of the fabricated structures, achieving needle tip sizes as small as 1 μm. Utilizing advanced 3D modeling software, the researchers designed and rapidly produced various MN configurations, including those with tailored densities, lengths, and shapes. SEM confirmed that the MNs achieved tip sizes between 1 and 55 μm and were effective at penetrating and breaking off in porcine skin. The study further demonstrated that the mechanical strength of the fabricated MNs is on par with existing designs. Additionally, PLA's capacity to swell was utilized to load small‐molecule drugs, while its degradable properties in the skin enabled a controlled release of these drugs over time. This technique provides a cost‐efficient and adaptable solution for manufacturing MNs, making it highly promising for applications in drug delivery and other biomedical areas.^66^

This study introduces a novel mobile skin bioprinting system designed for the efficient, on‐site treatment of extensive wounds. This system utilizes integrated imaging technology to accurately deliver either autologous or allogeneic dermal fibroblasts and epidermal keratinocytes directly to the injured area, effectively reconstructing the skin's natural layered architecture. Bioprinting excisional wounds with a HG scaffold containing these cells resulted in rapid wound closure, reduced contraction, and accelerated re‐epithelialization. The regenerated tissue closely mimicked healthy skin, displaying a dermal structure with organized collagen fibers, extensive vascularization, and actively proliferating keratinocytes (Figure 3f).^62^

In summary, 3D printed skin substitutes accelerate wound healing while offering personalized treatment options. Bioprinting for micronide systems and transdermal drug delivery provides painless and controlled dosage. Advances in this area could shape the future of personalized, effective, and minimally invasive treatments in bioprinted skin and drug delivery systems.

## 5 BIOPRINTING IN REGENERATIVE MEDICINE: THE POTENTIAL FOR ORGAN AND TISSUE REGENERATION

Bioprinting stands at the forefront of regenerative medicine, revolutionizing the way functional tissues and organs are created for therapeutic applications.^67^ This advanced technology combines the precision of 3D printing with biological materials, enabling the fabrication of complex structures that closely mimic the architecture and function of natural tissues. By integrating patient‐specific designs, biomaterials, and innovative bioprinting techniques, it addresses the critical challenge of organ shortages and offers promising solutions for transplantation needs.^68^ Bioprinting's ability to recreate vascular networks, neural pathways, and specialized organs like the heart and liver highlights its transformative potential.^69^ Despite current challenges in biomaterials and printing methods, this field continues to evolve, setting the stage for groundbreaking advancements in TE and regenerative therapies.^70^

### 5.1 Organ regeneration: building organs with bioprinting

Bioprinting technology represents a groundbreaking advancement in regenerative medicine, enabling the fabrication of complex, functional tissues and organs. A critical component of this process is bioinks, which provide the structural and biochemical framework required for cellular adhesion, migration, proliferation, and differentiation. Bioinks are primarily classified as natural (e.g., collagen, gelatin, and dECM) or synthetic (e.g., PEG and PCL) polymers, each offering unique advantages and challenges. Additionally, the inclusion of appropriate cell types—such as primary cells, stem cells, or engineered cell lines—is essential for replicating the cellular complexity of native tissues. This topic's importance lies in addressing the global shortage of donor organs and advancing personalized medicine. The integration of advanced bioinks, cell engineering, and 3D bioprinting technologies is paving the way for scalable, patient‐specific tissue and organ fabrication. Further details about the types of bioinks, their properties, and the cellular approaches in bioprinting are summarized in Table 1. Regenerative medicine refers to the process of restoring or replacing human cells, tissues, or organs to reinstate or establish normal function. It plays a vital role in repairing damaged tissues and organs by either replacing dysfunctional tissues or facilitating the healing of previously irreparable ones. Techniques used in organ regeneration encompass a variety of approaches, including cell transplantation therapies, organ generation from adult stem cells, decellularization and recellularization of scaffolds, in vitro organ development through TE, organ printing, and xenotransplantation. While fully regenerating a complex organ remains a long‐term goal fraught with challenges, cell therapy has become a feasible and practical approach. Cell therapy involves the injection or transplantation of cellular material into a patient. This therapeutic method can be categorized into two types. The first involves the transplantation of human cells from a donor to a patient, a technique with considerable potential for future advancements. Examples include the use of neural stem cells, MSCs, and hematopoietic stem cells in treating conditions such as osteogenesis imperfecta, Hurler's syndrome, myeloid malignancies, and other blood‐related disorders. One notable milestone was the approval in 2009 by the FDA for the first clinical trial using human embryonic stem cells (HESCs) for treating acute spinal cord injuries. Although preclinical trials showed success in improving motor functions in animal models, financial limitations led to the discontinuation of the trial. More recently, there have been advancements in functional recovery for patients with spinal cord injuries following the transplantation of olfactory ensheathing cells with nerve bridging. The second category of cell therapy involves the use of animal‐derived cells for treatment. However, this approach lacks substantial medical evidence supporting its effectiveness and can pose severe health risks to patients. This approach focuses on the generation of an entire organ from a single purified stem cell derived from the tissue. Successful creation of functional secretory mammary glands was achieved by transplanting single stem cells isolated from adult mouse mammary glands into the fat pad of mice. Similarly, Leong et al. demonstrated that a single stem cell taken from adult mouse prostate epithelium had the capability to form a functional prostate, using a colony‐formation assay and in vivo renal capsule transplantation technique. The blastocyst complementation system, initially described by Chen et al., involves the creation of chimeric animals with organs from another species. This technique is based on the principle that missing organs can be produced from exogenous cells when normal pluripotent stem cells (PSCs) are introduced into a cloned dysorganogenetic embryo. It has been successfully applied to generate rat pancreas and kidney derived from PSCs. This system offers a promising avenue for producing human organs by introducing PSCs into xenogeneic embryos, potentially enhancing organ supply. Decellularization involves removing the cells from tissues and organs to create acellular scaffolds that retain the ECM. These scaffolds can then be reseeded with appropriate progenitor cells. The resulting structures preserve the necessary microarchitecture and extracellular factors required for cellular attachment, differentiation, vascularization, and functional integration.^79^

**TABLE 1:** Natural and synthetic polymer bioink materials' mechanical properties, biocompatibility, cellular adhesion, applications, and structural stability are summarized in the table.

Bioink material	Source	Mechanical properties	Biocompatibility	Cellular adhesion	Applications	Structural stability	Refs.
Collagen	Natural	Low mechanical strength	High, biocompatible	High promotes cell adhesion	Tissue engineering, wound healing, skin engineering, and neuroengineering	Low structural stability, rapid degradation	71
Gelatine	Natural	Low mechanical strength	High, biocompatible	The medium ensures cell adhesion	Skin engineering, wound healing, tissue engineering	Low structural stability, limited cellular adhesion	72
Chitosan	Natural	Low mechanical strength	High, biocompatible	High indicates the ability to adhere to cells	Antimicrobial applications, wound healing, tissue engineering	Moderate structural stability, antimicrobial properties	73
Hyaluronic acid	Natural	Low mechanical strength	High, biocompatible	Low, but provides adhesion to some cells	Skin engineering, joint therapy, wound healing	Low structural stability, limited mechanical properties	74
PLA (polylactic acid)	Synthetic	High mechanical strength	Medium, biocompatibility	Moderate, limited cellular adhesion	Bone engineering, implants, biotechnology	High structural stability, biocompatibility	75
PEG (polyethylene glycol)	Synthetic	High mechanical strength	High, biocompatible	Low, poor cellular adhesion	Tissue engineering, biotechnology, artificial organs	Low structural stability, water retention capacity	76
PCL (poly(ε‐caprolactone))	Synthetic	High mechanical strength	High, biocompatible	Medium, cells adhere in limited numbers	Bone engineering, implants, tissue engineering	High structural stability, slow biodegradation	77
PVA (polyvinyl alcohol)	Synthetic	High mechanical strength	High, biocompatible	Moderate, limited cellular adhesion	Leather engineering, tissue engineering, and biotechnology	Moderate structural stability, biodegradable property	78

The 3D bioprinting technology of organs represents an approach that enables the customized and industrial‐scale production of living tissues. This technique enables 3D placement of multiple cell types at high densities and supports the creation of complex tissues. It enables the production of functional tissues containing basic vascular and neural networks. However, the production of more complex and functional organ‐like structures is still a significant challenge. Therefore, three key components, such as biomimetic structural modeling, bioactive materials, and a supportive bio‐micro‐environment, play a critical role in 3D bioprinting. These components need to be carefully planned and integrated for the successful production of organs by bioprinting. The development of this technology could lead to personalized and functional living organs for clinical use.^1^ Organ printing is an emerging technology that offers an alternative to traditional biodegradable scaffold‐based approaches in TE. This process involves creating 3D functional living tissues and organ constructs by layer‐by‐layer additive biofabrication, using tissue spheroids as building blocks. Organ printing follows three primary steps: the first is the pre‐processing stage, where blueprints for organs are developed through digitized image reconstruction of natural organs or tissues. The second step, processing, involves the actual printing of the organ by placing cells or cell aggregates into a 3D structure. The final step, post‐processing, includes perfusion of the printed organ to accelerate maturation.^80^ This technology operates on the principle of cellular self‐assembly, where cells organize themselves into tissues. Organovo, a leading company in the field, was the first to commercialize 3D bioprinting using their NovoGen MMX Bioprinter, which can print heart tissue, blood vessels, and skin, among other tissues. Bioprinting is also being explored for producing soft tissues and artificial bones for use in reconstructive surgery. Several studies have shown the potential of 3D bioprinting in generating tissue structures for skin, bone, and cartilage regeneration. For example, a bioresorbable customized tracheal splint was created using laser‐based 3D printing to treat a life‐threatening condition in an infant. Additionally, research has demonstrated the feasibility of printing blood vessel‐like constructs for clinical applications, utilizing hyaluronan HGs cross‐linked with polyethylene glycol tetraacrylates. Heart valves have also been printed with multiple cell populations to create mechanically living tri‐leaflet valves. While scientists are currently working to develop functional kidneys, bladders, and hearts using this technology, fully functional organs are still not a reality. The organ models created so far, such as small versions of the heart and kidney, have limited lifespans of only a few days instead of years.^81^

#### 5.1.1 Nerve bioprinting

To promote nerve regeneration, HGs can be integrated with growth factors during bioprinting to enable controlled, gradual release that supports neural regeneration. For instance, in one study, neural stem cells, collagen HG, and fibrin gel that was preloaded with VEGF were bioprinted to create artificial neural tissue. When neural stem cells were positioned 1 mm from the edge of the VEGF‐releasing fibrin gel, they exhibited morphological changes induced by the VEGF and migrated toward the fibrin gel. These findings showed that bioprinting with VEGF‐containing fibrin gel facilitates the sustained release of growth factors within a collagen scaffold. This approach can be useful in developing 3D tissue models for neural tissue regeneration applications.^82^ In the design of tissue‐engineered nerve grafts, incorporating multi‐luminal channels may enhance axon regeneration. 3D bioprinting technology has been used to create nerve grafts with such multi‐lumen channels. Owens et al. developed a bioprinting technique to produce fully biological grafts consisting of cells and cell‐secreted materials. In their study, MSCs and Schwann cells were combined and used to create cellular cylinders (~500 μm in diameter). These cylindrical structures were stacked together to form multi‐lumen channels, which fused during the bioprinting process. After a 7‐day maturation period, the multichannel graft demonstrated sufficient mechanical integrity for implantation into laboratory rats. This research illustrates the potential of bioprinting in nerve graft fabrication, showing promising results for nerve regeneration.^83^

#### 5.1.2 Blood vessels bioprinting

Blood vessels play a critical role in maintaining tissue and organ functionality by transporting nutrients, oxygen, and metabolic waste. Damage to these structures, caused by trauma or diseases such as cardiovascular diseases (CVD), can severely impact body systems. The creation of functional blood vessels or tissue scaffolds for replacement has thus become a key focus in regenerative medicine. Early efforts in bioprinting vascular structures faced significant challenges, primarily due to the lack of mechanical stability and functional integration. However, recent advancements in materials and techniques have opened new avenues for engineering both large and small‐diameter blood vessels. Initial bioprinting strategies employed spheroids and agarose‐based scaffolds to construct vascular structures. While these supported cell attachment and proliferation, their inadequate mechanical strength limited their functionality. Researchers then introduced alginate‐based scaffolds, aiming to enhance cell‐matrix fusion. Although this improved mechanical properties slightly, it still fell short of the necessary stability for long‐term applications. To overcome the limitations of earlier materials, researchers explored embedding cells directly into HGs. Kreimendahl et al. used mixtures of agarose and collagen to promote endothelial cell and fibroblast growth, yielding promising results. Multilayered constructs made from gelatin‐fibrin further improved cell alignment but exhibited limited permeability—a crucial factor for functional blood vessels. Without adequate permeability, these structures risk inflammation from fluid buildup. Microfluidic bioprinting emerged as a promising approach to create vascularized tissues. For example, HGs composed of PEG/fibrinogen with embedded endothelial cells (ECs) demonstrated enhanced vascular networks when implanted in mice. Another technique, DLP, enabled the bioprinting of vascular networks within tissues using GelMA bioinks loaded with ECs. Innovations like catechol‐functionalized GelMA have also improved mechanical properties and vasoactivity, advancing the creation of realistic vascular microstructures. Engineering small‐diameter blood vessels (less than 6 mm in diameter) remains a significant challenge. These vessels consist of three primary layers:

1. The inner layer of vascular endothelial cells (VECs) regulates blood flow and vessel permeability.
2. The middle layer of vascular smooth muscle cells (VSMCs) provides structural support.
3. The outer layer of connective tissue stabilizes the vessel.

**FIGURE 4:**
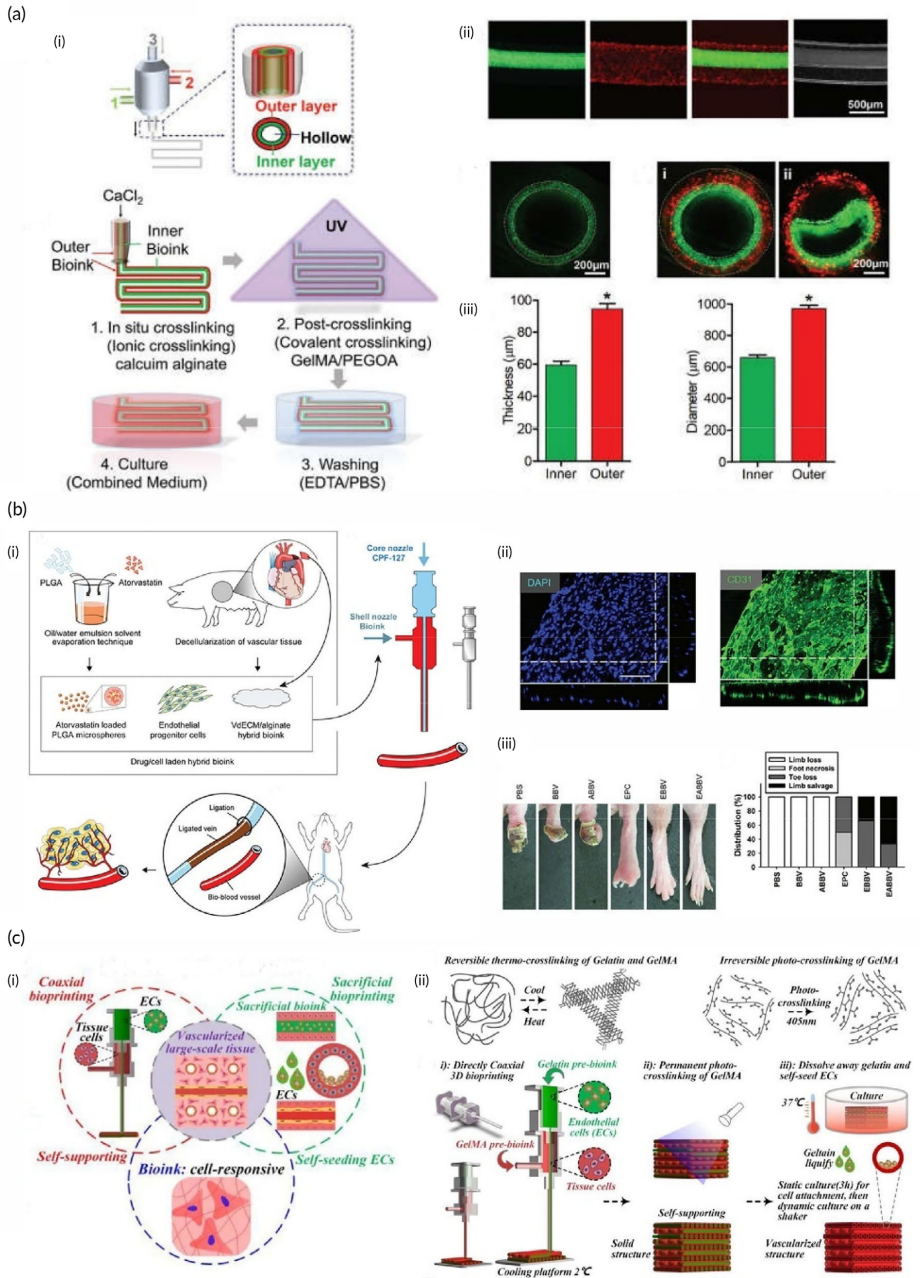
Schematic summaries of various 3D bioprinting approaches for creating vascularized and multilayered tubular tissue constructs. (a) Bioprinting of multilayered tubular tissues: Hollow multilayered tubular structures fabricated by coaxial bioprinting demonstrated clear separation of layers and precise control of tube dimensions, confirming the preservation of structural integrity.^87^ (b) Cell/drug‐loaded biomimetic blood vessels: Cell or drug‐loaded bioprinted vessels promoted vascularization by inducing endothelial progenitor cells to form a confluent endothelial layer and showed therapeutic effects in an ischemic limb model.^88^ (c) Development of vascularized constructs: Coaxial and sacrificial bioprinting strategies have enabled the formation of self‐supporting vascularized constructs, and mechanical stability and functional vascularization of the construct are ensured by the cross‐linking mechanism.^89^ 3D, three‐dimensional; ECs, endothelial cells; EDTA, ethylenediaminetetraacetic acid; GelMA, gelatin methacryloyl.

Successfully replicating these layers requires advanced techniques such as coaxial cell printing. Zhou et al. fabricated two‐layered engineered blood vessels using VSMC‐laden bioinks and seeded VECs into the tube wall. By degrading alginate in the bioink, they achieved better spreading and proliferation of VSMCs. Pi et al. further enhanced coaxial printing with multichannel extrusion systems, simplifying the construction of multilayer vascular tissues. Despite these advancements, more research is needed to improve angiogenesis potential. Coaxial bioprinting has emerged as a leading method for creating tubular structures. This technique allows for precise deposition of bioinks containing distinct cell types, such as endothelial and smooth muscle cells (SMCs), to replicate the layered architecture of blood vessels. Sacrificial bioprinting complements this by enabling the construction of complex vascular networks. For instance, Shao et al. used sacrificial gelatin‐based bioinks containing ECs to create vascularized constructs. Once the gelatin dissolved, ECs adhered to the channel walls, forming functional vascular networks.^79^ Microchannel bioprinting, involving the deposition of fugitive ink HGs, provides a platform for creating perfusable vascular structures. These microchannels allow ECs to form monolayers that replicate blood vessel geometry. Angiogenesis occurs when proteases degrade the HG, prompting ECs to sprout new vessels. The channel's curvature significantly influences angiogenesis, making this method highly adaptable for therapeutic applications such as treating ischemia or restricting angiogenesis in cancer. Enhancing angiogenesis is a central goal in bioprinting. Gao et al. developed endothelial progenitor cell (EPC)‐laden blood vessels using a hybrid bioink composed of vascular tissue‐derived extracellular matrix, alginate, and atorvastatin‐loaded microspheres. This bioink promoted EPC proliferation and differentiation, leading to effective angiogenesis in ischemic tissues. To integrate vascular constructs with surrounding tissues, researchers have employed co‐culturing strategies involving multiple cell types. For instance, pericytes and SMCs support the structural stability of vascular networks, while electrical stimulation has been used to enhance cell alignment and function. Innovations like spheroid‐based bioprinting also enable the creation of cell‐dense constructs that mimic the complexity of native tissues.^84^ Bioprinting vascularized tissues has wide‐ranging applications in regenerative medicine. In cardiac TE, combinations of cardiomyocytes (CMs), fibroblasts, and ECs have been used to recreate vascular networks with conductive and contractile properties. Similarly, liver and kidney bioprinting often involve co‐printing parenchymal cells with vascular cells to replicate organ‐specific functions like zonation and filtration. The success of bioprinting depends on the properties of the bioinks used.^85^ These materials must mimic the native ECM to support cell adhesion, migration, and differentiation. Bioinks derived from decellularized organs or engineered to include ECM components are widely used. Properties such as mechanical strength, biodegradability, and bioactivity are critical for ensuring tissue maturation post‐bioprinting. While significant progress has been made, several challenges remain in scaling up bioprinted constructs and achieving functional integration with host tissues. Addressing these issues requires advances in biomaterial engineering, vascularization techniques, and bioprinting technologies. As these hurdles are overcome, bioprinting holds immense potential for transforming regenerative medicine, from creating functional tissues and organs to developing in‐vitro models for disease research and drug testing.^86^ Pi et al. introduced a modified coaxial needle that enabled a multichannel extrusion approach, allowing multilayer vascular tissues to be fabricated in a single bioprinting step (Figure 4a).^87^ Gao et al. designed a 3D coaxial‐printed bio‐blood‐vessel using a hybrid bioink composed of vascular tissue‐derived dECM, alginate, and PLGA microspheres loaded with atorvastatin, encapsulating EPCs (Figure 4b).^88^ These constructs not only demonstrated potential for direct application in treating limb ischemia but also hold promise as regenerative implants to enhance vascularization. Furthermore, by integrating sacrificial printing with coaxial bioprinting, Shao et al. successfully generated large‐scale constructs containing vascularized channels (Figure 4c).^89^

#### 5.1.3 Cardiac tissue bioprinting

CVDs remain among the leading causes of mortality globally, particularly in developed nations. Each year, approximately 8 million myocardial infarction cases are reported worldwide, alongside conditions such as heart valve stenosis.^90^ These conditions are often associated with irreversible loss of CMs, which lack regenerative capacity. This deficit is typically compensated by the formation of non‐functional scar tissue, significantly increasing the risk of acute cardiomyopathy. Current treatment modalities include coronary artery bypass grafting, cell therapies, and left ventricular assist devices, with heart transplantation serving as the final option. However, the limited availability of donor hearts and the risk of immune rejection pose substantial barriers to transplantation success. TE offers a promising alternative for addressing damage to cardiac valves, blood vessels, and other cardiovascular structures. Traditional methods involve culturing cells on biocompatible scaffolds made from materials such as HGs or decellularized tissue matrices, which mimic the native ECM.^91^ Among emerging technologies, 3D bioprinting stands out as a method for precisely assembling biomaterials layer by layer, enabling the reconstruction of the complex architecture of cardiac tissue. This technology shows potential to address challenges such as the myocardium's auto‐rhythmic properties and the integration of diverse cell types, including CMs, fibroblasts, and ECs. The process of cardiac bioprinting involves three key phases: pre‐processing, printing, and post‐processing.

1. Pre‐processing: The initial step involves creating patient‐specific 3D models based on clinical imaging data, such as MRI, CT, or PET (Positron Emission Tomography) scans. These images are segmented using advanced techniques to isolate cardiac tissue structures, and the resulting digital models are converted into STL files, which are prepared for printing.
2. Printing: Bioprinters are used to fabricate constructs by layering biomaterials and cells to mimic the natural structure of cardiac tissue. Different cell types are strategically placed to replicate the functional properties of native tissue.
3. Post‐processing: After printing, the constructs undergo maturation in bioreactors, where their ability to perform key functions such as contraction and perfusion is assessed. This step is crucial to ensure the printed tissue can sustain the heart's natural contraction‐relaxation cycles, which occur approximately 70–80 times per minute at rest. Adequate vascularization and tissue perfusion remain critical challenges at this stage.

Recent studies have demonstrated the potential of 3D bioprinting to develop cardiac patches that enhance the function of infarcted hearts. While these patches have shown promising integration into living systems, further research is required to evaluate their mechanical stability, functional performance, and scalability for larger animal models. One of the primary challenges in advancing cardiac bioprinting is achieving sufficient vascularization, particularly in larger organisms where myocardial layers are thicker and require extensive blood supply. In addition to cardiac applications, 3D bioprinting has shown utility in developing disease models, toxicology studies, and personalized drug testing. The technology also holds promise for addressing heart valve diseases by enabling precise replication of native valve structures. Unlike traditional methods of seeding decellularized organs with cells, 3D bioprinting offers superior spatial control and the ability to accurately position various cell types, particularly when integrated with stem cell technologies.^92^

#### 5.1.4 Heart bioprinting

Noor et al. developed a bioink using induced PSCs derived from patients' adipocytes, combined with ECM components such as collagen, to create a miniature heart. This bioengineered heart included atria, ventricles, and major blood vessels. The use of patient‐derived cells as the main component of the bioink ensures that the resulting artificial organ closely matches the individual's immune, cellular, and biochemical properties. Additionally, significant advancements have been made in the functional modeling of 3D‐printed biological organs. For instance, Jallerat et al. devised a novel bioprinting technique to fabricate heart structures by printing collagen layers as the inner and outer walls with CMs embedded between them, resulting in left ventricle and heart valve models that demonstrated spontaneous beating after in vitro culture. Researchers from the University of Erlangen‐Nuremberg in Germany have also achieved notable progress by creating a miniature heart chamber capable of autonomous beating for over 3 months. Despite these advancements, replicating the intricate structure and functions of the myocardium remains a significant challenge. Moving forward, the potential of 3D‐printed artificial ventricles to aid patients with heart disease, particularly those awaiting transplants, looks promising. However, critical hurdles still exist, including ensuring compatibility with the human body, maintaining the longevity of printed tissues, and integrating the artificial ventricles effectively with other organ systems. Further research is required to address these challenges.^84^ The bioprinting of 3D heart‐like structures has been explored using a variety of biomaterials and cells (bioink). Materials such as alginate‐ColMa‐CNTs, PCL‐CNTs, and PLGA have been used to fabricate scaffolds and assess their biocompatibility in printing cardiac tissues or partial organs. In the future, scaffold‐based techniques are expected to enhance tissue printing by incorporating properties like electrical conduction and increased cell‐to‐cell interaction. In contrast, scaffold‐free 3D printing approaches are also being investigated, where cells or spheroids are directly printed onto a substrate. This method holds an advantage over scaffold‐based techniques by reducing risks of immune reactions and toxicity associated with scaffolds. Notably, scaffold‐free patches fabricated using spheroids begin contracting within 3 days, demonstrating uniform cell alignment, enhanced cell‐to‐cell interaction, and improved electrical conductivity. Consequently, this approach has shown promise in the development of functional tissue patches. Despite these advances, scaffold‐free and scaffold‐based methods still face challenges in mimicking the complex microvascular networks of the heart. The creation of such networks and replicating the intricate heart microenvironment remain critical objectives. For instance, a 3D cardio‐patch developed using CMs demonstrated encouraging results, including the expression of myocardial markers, strong electromechanical coupling, and rhythmic contractions. Future advancements in personalized therapy for myocardium regeneration will likely require more than scaling up printed tissues. It will necessitate selecting cells that can be expanded effectively to fabricate larger, viable tissues. PSCs are a promising option for generating cardiovascular patches. Additionally, factors such as proper perfusion, development of microvascularization, and minimizing immune responses are essential to ensure the survival and functionality of the tissue post‐implantation. Addressing these challenges, as well as currently unknown factors, will be necessary to achieve fully functional heart printing. Although there has been significant progress, substantial work remains for 3D bioprinting to be fully realized in cardiac tissue and organ therapy.^93^

**FIGURE 5:**
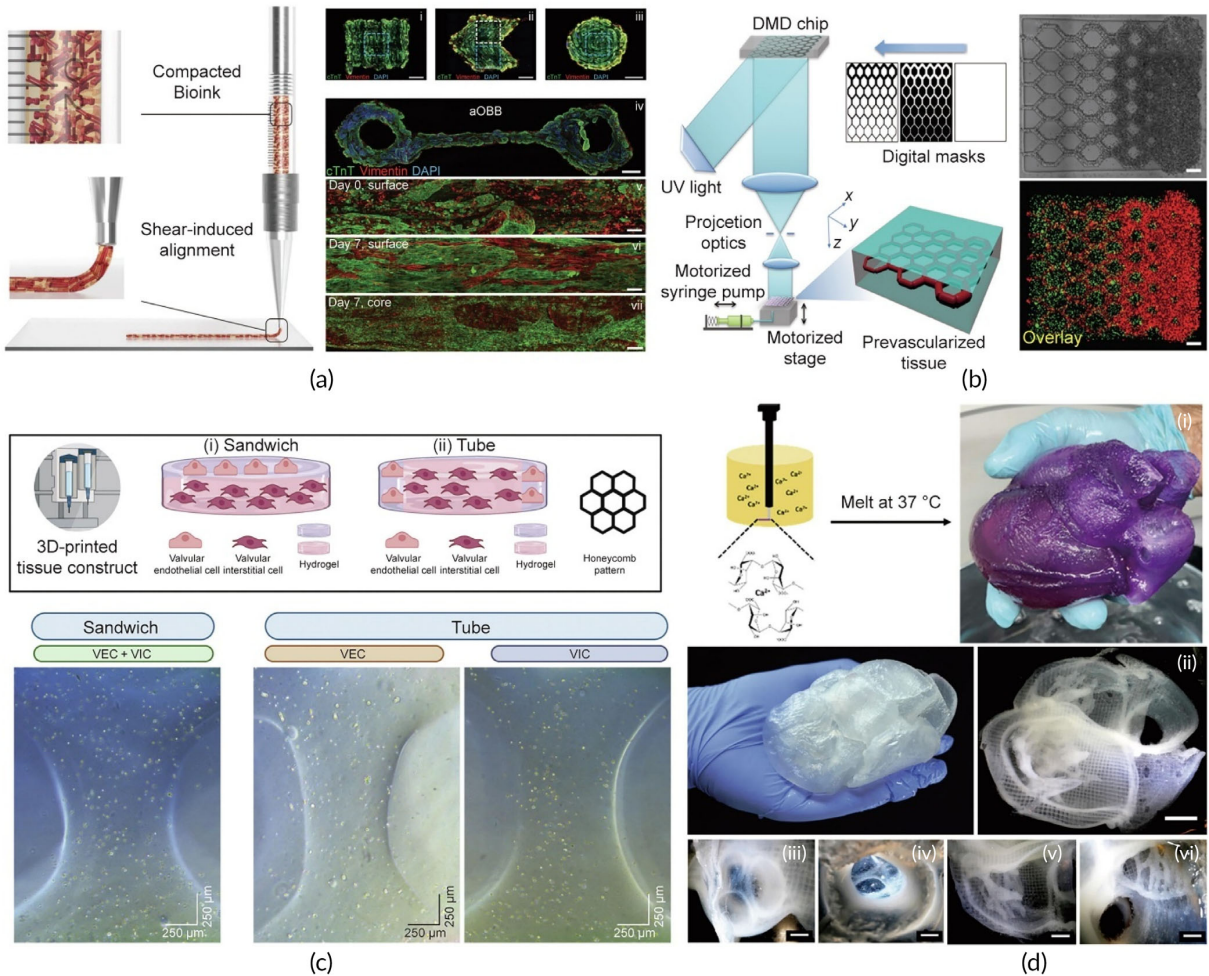
Schematic summaries of recent advances in 3D cardiac bioprinting and tissue engineering. Advances in cardiac bioprinting. (a) Engineered cardiac tissue with programmable alignment via bioprinted anisotropic organ building blocks (aOBBs) showed preferential cellular orientation after 7 days in culture, demonstrating controlled tissue organization.^97^ (b) Microscale continuous optical bioprinting enabled the creation of complex prevascularized tissue constructs, supporting organized microvascular networks.^98^ (c) Co‐cultured valvular interstitial cells and endothelial cells formed a functional multicellular construct, highlighting successful valvular tissue engineering.^99^ (d) Freeform Reversible Embedding of Suspended Hydrogels (FRESH) bioprinting produced a full‐size human heart model with anatomically accurate internal features, illustrating the feasibility of large‐scale cardiac tissue fabrication.^100^

Bioprinting offers the potential to create complex tissues and organs, revolutionizing regenerative medicine. This technology uses cells and bioinks to rebuild vital structures such as heart muscle, valves, and vascular networks. Developed with patient‐derived stem cells and ECM components such as collagen, bioinks enable personalized treatments by reducing the immune response. While scaffolded and scaffold‐free bioprinting techniques used in cardiac TE have shown promise in terms of cellular assembly and electrical conductivity, challenges remain, such as vascularization and precisely mimicking biomechanical properties. However, the development of heart chambers, bioactive patches, and even autonomously beating organoids has provided groundbreaking advances in this field. Future studies focusing on issues such as scalability, long‐term functionality, and integration of vascular networks will further enable clinical applications of bioprinting. Spheroid‐based bioprinting not only achieves cardiac‐like high cell densities but also facilitates the engineering of spatial heterogeneity in cardiac tissues. For example, Daly et al.^94^ developed a reductionist model of focal cardiac fibrosis using cell‐conditioned medium (CM)/FB (fibroblast) spheroids. By manipulating the ratio of iPSC‐derived CMs to FBs in the spheroids (4:1 for “healthy” and 1:4 for “scarred”), the researchers created microtissue rings exhibiting spatially controlled CM and FB ratios with distinct properties in healthy and scarred areas, including variations in contraction and electrophysiological characteristics. These microtissue rings can serve as a model for cardiac fibrosis, enabling the study of therapeutic interventions for cardiac repair. The native myocardium features anisotropically aligned muscle fibers that contribute to left ventricular torsion and are essential for normal ejection function. Several approaches based on bioprinting have been reported to induce oriented cell alignment in the myocardium. Zhang et al.^95^ introduced a scaffold‐based method in which the bioprinting of EC‐embedded microfibrous scaffolds guided the orientation of subsequently seeded CMs, enabling the fabrication of endothelialized myocardium. In addition to surface seeding, anisotropic myofibers have been created using microscale continuous optical printing, wherein cells were encapsulated inside patterned GelMA scaffolds. The aligned fibers, with ventricular CMs encapsulated in a 3D environment, generated nearly twice the force compared with two‐dimensional (2D)‐seeded controls. Patterned scaffolds with different alignments exhibited varied contractile forces, all surpassing the controls on flat surfaces. Tsukamoto et al.^96^ developed orientation‐controlled cardiac tissue using a layer‐by‐layer method combined with bioprinting. In this approach, cell direction was manipulated by linearly shaping the cardiac tissue with a 3D‐printed gel frame. A novel approach for programming cellular alignment was utilized to engineer anisotropic organ building blocks (aOBBs) made of iPSC‐CMs, which were incorporated into a compacted bioink for extrusion‐based bioprinting. The aOBBs consisted of elongated microtissues composed of aligned CMs. During extrusion, the shear and extensional forces resulted in the alignment of aOBBs along the print path, forming macro‐filaments containing oriented CMs (Figure 5).^101^

#### 5.1.5 Trachea bioprinting

**FIGURE 6:**
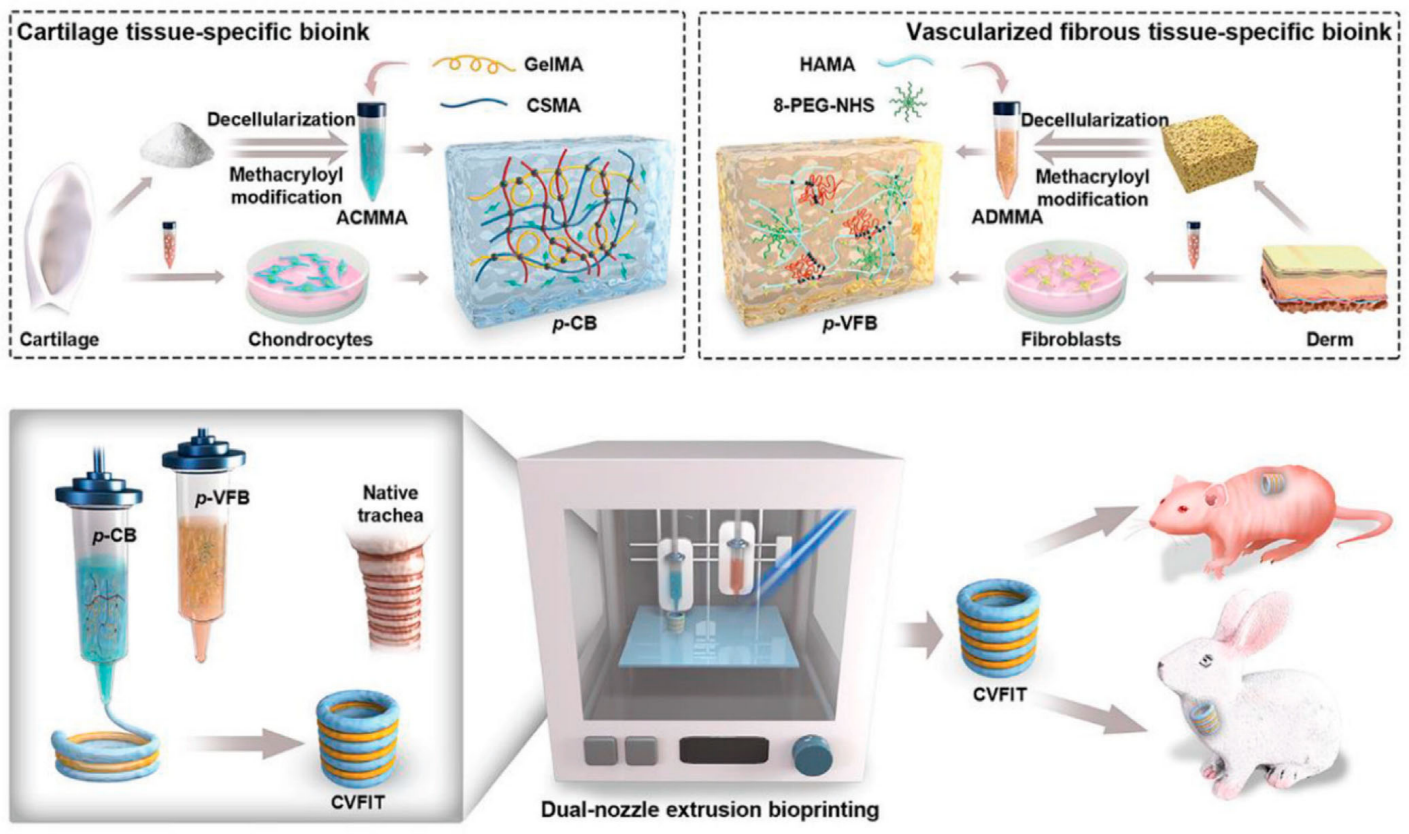
Schematic summary of a 3D bioprinted trachea integrating cartilage ans vascularized fibrous tissue using tissue‐specific bioinks. Schematic abstract, three‐dimensional bioprinted trachea were created using cartilage and vascularized fibrous tissue‐specific bioinks in mice and integrated by in situ reconstruction in rabbits; this approach demonstrates the successful integration of cartilage and vascularized fibrous tissues and the functional trachea structure. 8‐PEG‐NHS, 8‐arm polyethylene glycol‐succinic acid ester; ACMMA, methacryloyl‐modified acellular cartilage matrix; ADMMA, methacryloyl‐modified acellular derm matrix; CSMA, methacrylate‐modified chondroitin sulfate; CVFIT, cartilage‐vascularized fibrous tissue‐integrated tracheal; GelMA, gelatin methacryloyl; HAMA, methacrylate‐modified hyaluronic acid; p‐CB, photocrosslinkable cartilage‐specific bioink; p‐VFB, photocrosslinkable vascularized fibrous tissue‐specific bioink.^103^

The versatility of 3D bioprinting proves especially beneficial for creating tracheal scaffolds that match the precise anatomical geometry and shape required for effective reconstruction. In one study, a 3D‐printed PCL scaffold was used in an in vivo tracheal defect model for reconstruction. The PCL scaffold was coated with MSCs seeded in fibrin, and the graft was implanted to cover a 10 × 10 mm^2^ tracheal defect in rabbits. After 4 and 8 weeks, the reconstructed trachea exhibited successful integration, without collapse or obstruction. Histological analysis revealed that the bioprinted trachea underwent remodeling, developing a regenerated respiratory mucosa and fusing seamlessly with the surrounding tracheal tissues without the formation of granulation tissue. Furthermore, the newly formed neocartilage inside the graft maintained sufficient mechanical strength to support the trachea's structural integrity. Functionally, the implanted 3D‐printed PCL trachea effectively integrated with the adjacent tissue, enabling basic respiratory functions. The combination of 3D bioprinting with medical imaging offers the opportunity to create customized, patient‐specific tissues for implantation. In a recent study, a bioresorbable tracheal splint made of PCL was fabricated using laser‐based 3D printing, with a design based on the patient's airway as obtained from a CT scan. One year following the surgery, imaging and endoscopic evaluations showed that the left mainstem bronchus had normal structure and function. This case highlights the potential of using 3D bioprinting alongside imaging technologies to develop anatomically precise TE solutions tailored to individual patients. In June 2011, Macchiarini and colleagues successfully performed a tracheal transplant using an artificial trachea in a 36‐year‐old patient suffering from advanced tracheal cancer. The procedure involved extracting stem cells from the patient's hip, which were then treated with growth factors and cultured on a plastic mold resembling the patient's natural trachea. This approach has the benefit of not requiring a donor, allowing for the trachea to be replaced within a few days. Torsello et al. studied a bioprinted PCL scaffold embedded with autologous MSCs for laryngotracheal reconstruction in an ovine model.^102^ The findings showed varied results, with two animals achieving full scaffold integration accompanied by the development of respiratory epithelium on the scaffold surface, while the remaining two experienced post‐operative inflammation and respiratory distress, attributed to the scaffold's mechanical rigidity. The scaffold, used as a “patch” to repair segmental tracheal defects, lacked spatial similarity to the native trachea. Conversely, Huo et al. introduced an innovative approach involving 3D bioprinting to fabricate a cartilage‐vascularized fibrous tissue‐integrated tracheal (CVFIT) structure. This design utilized photocrosslinkable bioinks engineered to replicate the trachea's natural heterogeneity. In vivo results demonstrated effective tracheal regeneration, showing favorable mechanical characteristics and essential physiological functions, including vascularization and epithelialization (Figure 6).^103^

#### 5.1.6 Alveoli bioprinting

Alveoli, the smallest and terminal structures of the respiratory system, are essential for gas exchange between the lungs and capillaries. Their small, balloon‐like shape provides a high surface area and thin walls, optimizing the diffusion of gases. These structures often form clusters distributed throughout the lungs. Due to their intricate architecture and microscopic size, replicating alveoli through bioprinting poses a significant challenge. Current extrusion‐based bioprinters, limited by a resolution of approximately 100 μm, face difficulties in achieving the detailed structure of alveoli due to the mechanical stress applied to cells during the printing process. Advanced techniques such as SLA, laser‐induced bioprinting, and other droplet‐based approaches have also struggled to produce the high‐resolution, single‐ or multi‐cell precision required to replicate the alveolar barrier. Consequently, achieving a full recreation of functional alveoli at the 3D resolution level has proven to be highly complex and has seen limited success. Notable progress in modeling alveoli began with Huh et al.,^104^ who developed a dynamic alveolar model using poly(dimethylsiloxane) to simulate breathing movements. Building on this, Horvath et al. utilized Matrigel® to position epithelial and ECs onto a porous membrane, demonstrating an alternating bioprinting strategy. More recently, Grigoryan et al.^105^ advanced the field by creating a sophisticated lung alveolar model that included interconnected vascular structures and airway spaces, representing a step toward replicating the functionality of natural alveoli.^106^

#### 5.1.7 Lung bioprinting

Rebuilding blood vessels and tracheal structures remains a significant hurdle in the 3D printing of lungs. These components are essential for replicating the physiological functions of the respiratory system. Grigoryan et al. addressed this challenge by developing 3D‐printed lung models that mimic vascular and tracheal structures. Using projection SLA, they employed food‐grade dye additives as biocompatible light absorbers, enabling the rapid fabrication of intricate vascular systems within a transparent HG. This innovation allowed the creation of functional lung models capable of simulating oxygen transport and breathing. Another application of 3D printing in lung research involved Axial3D and the Health and Social Insurance Foundation in Belfast. Together, they created scale models of lungs affected by COVID‐19, based on CT scans from infected patients. These models provided valuable insights into lung damage caused by the virus, offering a revolutionary tool for visualizing the respiratory system and understanding the long‐term impacts of infection. The lungs are designed for efficient gas exchange, comprising distinct zones that perform specialized roles. The conducting zone, which includes the trachea, bronchi, and bronchioles, is lined with pseudostratified ciliated epithelium that facilitates airflow and mucociliary clearance. In contrast, the respiratory zone contains respiratory bronchioles and alveoli, where gas exchange occurs. Alveoli are lined by two types of pneumocytes: Type I pneumocytes, responsible for diffusion, and Type II pneumocytes, which secrete surfactant to reduce surface tension. Together, these structures enable the critical function of respiration. Bioprinting lung tissues requires a comprehensive understanding of these anatomical and physiological features. Dividing the lungs into smaller functional units with unique cavities, similar to strategies used for other hollow organs, has proven effective. This approach allows the construction of customized, physiologically accurate lung tissues tailored to individual patients' needs. Several innovative approaches have emerged in lung bioprinting. Kim et al. introduced a promising technique for tracheal reconstruction to address segmental tracheal defects. Kang et al. used inkjet bioprinting to create a 3D alveolar barrier model featuring four distinct alveolar cell types. Similarly, da Rosa et al. differentiated MSCs from Wharton's jelly into various lung cell types and bioprinted them using alginate/gelatin bioink. Ng et al. developed multilayered alveolar lung models comprising epithelial, endothelial, and fibroblast cells through drop‐on‐demand bioprinting, demonstrating cell viability and proliferation comparable to non‐bioprinted structures. Recreating vascular networks is critical for supporting cell survival and growth in bioprinted lung tissues. Grigoryan et al. demonstrated success in engineering 3D transport systems using photopolymerizable HGs. These systems formed complex vascular networks and functional valves, essential for effective gas exchange. Horváth et al. advanced the field by developing a 3D alveolar model that replicates the human air‐blood barrier, suitable for high‐throughput applications despite limited cell survival periods. Optimizing the structural and functional parameters of lung units is vital for advancing bioprinting techniques. Improvements in these areas will enable the reconstruction of intricate microenvironments and functional units, essential for realizing fully bioprinted lung tissues. By integrating cutting‐edge materials, biocompatible technologies, and a deeper understanding of lung physiology, researchers aim to overcome current limitations and unlock the potential of lung bioprinting for regenerative medicine. While significant progress has been made in bioprinting human lung models, challenges remain in replicating the complex structures and functions of the organ. Advances in vascular network engineering, cell differentiation, and 3D bioprinting technologies continue to pave the way for breakthroughs in this transformative field.^84^

#### 5.1.8 Liver bioprinting

**FIGURE 7:**
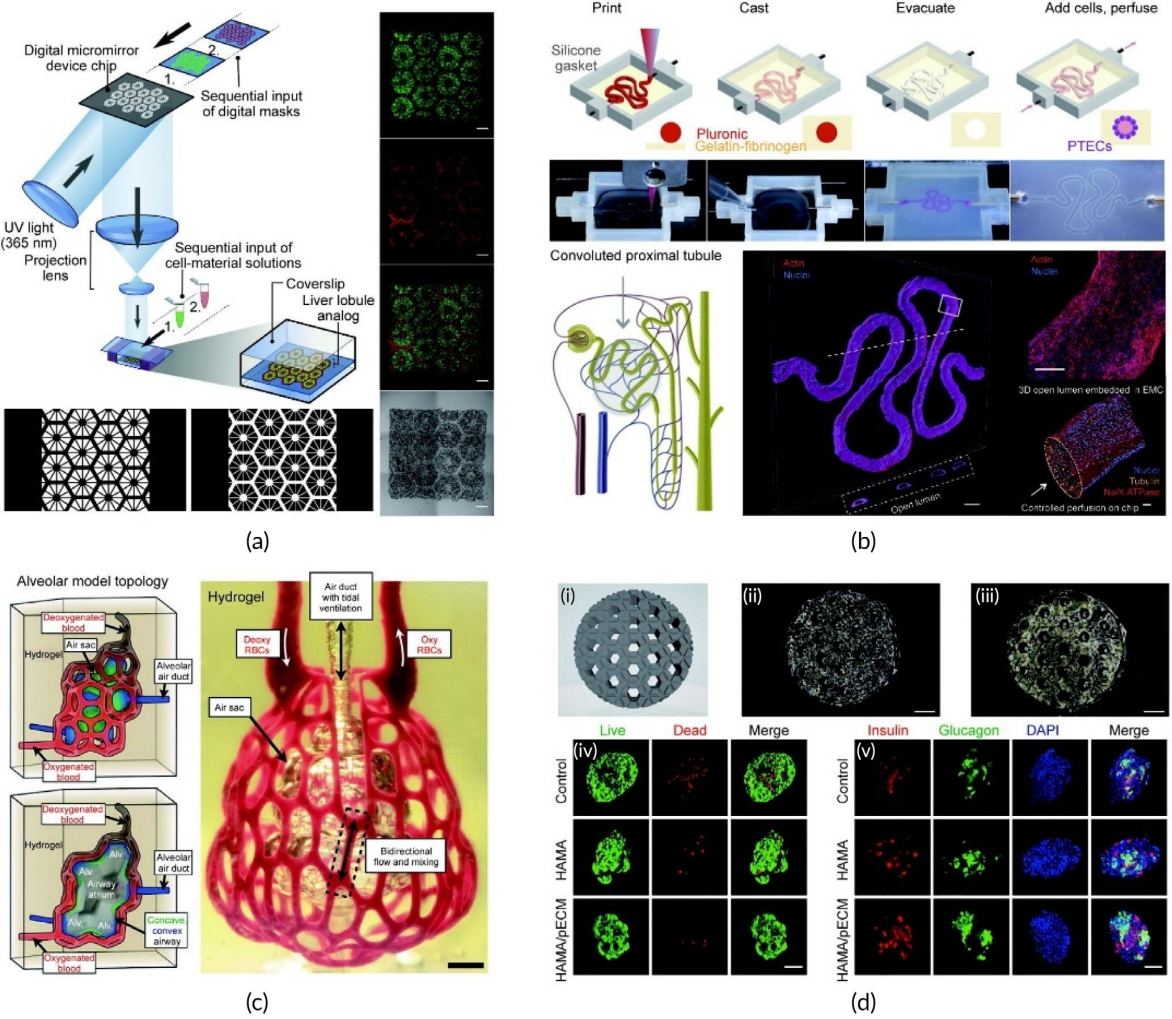
Schematic summaries of recent advances in 3D bioprinting of vital organs including the liver, kidney, lung, and pancreas. Advances in three‐dimensional (3D) bioprinting of vital organs such as the liver, kidney, lung and pancreas have been demonstrated. (a) Liver lobule model created by digital light processing‐based bioprinting shows organized and vibrant distribution of hepatocytes (green) and supporting cells (HUVEC (Human Umbilical Vein Endothelial Cells) and adipose‐derived stem cells, red) (scale bars: 500 μm).^112^ (b) Kidney units were created within tortuous 3D tubules using sacrificial bioprinting, and renal epithelial and endothelial cells were successfully grafted onto the inner wall of the tubules (scale bars: 50–500 μm)^113^; (c) Bio‐inspired in vitro vascularized alveolar models designed by 3D printing support cellular organization and vascularization (scale bar: 1 mm)^105^; (d) 3D‐printed methacrylate hyaluronic acid (HAMA)/pECM (pancreatic Extracellular Matrix) islet organoids demonstrate that scalable and organized structures can be generated in vitro (scale bars: 50 μm–2 mm).^114^

The liver, a vital organ in the human body, plays a central role in metabolism, detoxification, and bile production. Its complex structure and multifaceted roles make it particularly challenging to replicate using TE techniques. Recent advancements in 3D bioprinting have opened new avenues for creating liver models and constructs that can address various medical challenges, including drug testing, disease modeling, and organ transplantation. This document synthesizes insights from multiple studies to present an overview of liver bioprinting and its potential in regenerative medicine. The liver's functional unit, the hepatic lobule, is a hexagonally structured unit approximately 1 mm in length and 2 mm in thickness. Each lobule comprises a central vein surrounded by radiating cell cords that extend to portal triads at the periphery. The triads consist of the hepatic artery, bile duct, and portal vein. Between these cords, fenestrated endothelial cells (SECs) form sinusoids, enabling substance exchange and zonation of hepatocyte activity. Supporting cells, including Kupffer cells, hepatic stellate cells (HSCs), and cholangiocytes, contribute to essential liver functions such as pathogen removal, fibrosis regulation, and bile management. The liver's remarkable regenerative ability allows rapid recovery from damage through hepatocyte proliferation. However, replicating this capability using bioprinting is challenging due to the organ's intricate structure and diverse cellular composition. To address the complexity of liver reconstruction, researchers have developed liver‐on‐a‐chip systems for in vitro drug testing. For example, Lee et al. created a 3D liver‐on‐a‐chip model incorporating multiple liver cell types, ECM microenvironments, and vascular and biliary fluidic systems.^107^ This model utilized decellularized ECM bioink to mimic the native liver environment and integrated biliary fluidic tubes to enhance bile duct development and hepatic functionality. The system demonstrated a robust response to acetaminophen exposure, confirming its potential for drug testing. Faulkner‐Jones et al. utilized inkjet bioprinting to differentiate hepatocyte‐like cells (HLCs) from human‐induced pluripotent stem cells (HiPSCs) and HESCs.^108^ The cells expressed nuclear factor 4α (NF4α) and secreted albumin, indicating hepatocyte functionality. Their study demonstrated that HiPSCs retained high viability and pluripotency post‐printing, offering a pathway for creating miniature liver constructs for drug screening. Lei and Wang employed a multi‐nozzle extrusion‐based 3D bioprinter to fabricate liver constructs with branched vascular systems. Their approach combined adipose‐derived stem cells (ADSCs), primary hepatocytes, and a four‐nozzle cryodeposition technique to generate vascularized liver‐like structures.^109^ These constructs exhibited improved functionality and structural integrity, showcasing the potential of combinatorial methods in liver bioprinting. Despite these advancements, replicating the liver's intricate architecture and vascular systems remains challenging. Traditional methods have struggled to achieve accurate emulation of the liver's structure and biomechanical properties. Efforts to create liver sinusoid models with integrated vascular networks are ongoing, with researchers exploring techniques such as sacrificial bioprinting, extrusion‐based bioprinting, and coaxial bioprinting. Chang et al. developed a micro‐organ device tailored for dynamic drug screening, while Bhise et al. introduced a liver‐on‐a‐chip platform capable of supporting long‐term hepatocyte cultures. These systems allow for rapid prediction of drug‐induced toxicity, reducing reliance on traditional in vivo studies. Sun et al. designed a 3D‐printed liver model that revealed variations in tumor‐related gene expression, advancing antitumor drug screening. Similarly, Norona et al. presented a model for studying hepatic fibrosis and the functional roles of Kupffer cells.^110^ Maji et al. introduced a single‐step bioprinting approach to develop perfusable vascularized liver sinusoid in vitro models (LSOC‐P).^111^ These constructs demonstrated enhanced hepatocyte viability, proliferation, and liver‐specific gene expression compared to traditional models. Sacrificial bioprinting has been employed to construct vascularized liver tissue models. However, significant challenges remain in scaling up these technologies for widespread use. Future research should focus on refining vascularization techniques, improving bioink formulations, and integrating bioreactors to enhance construct maturation (Figure 7).^115^

#### 5.1.9 Kidney bioprinting

Ali et al. developed a photocross‐linked bioink composed of gelatin, HA, glycerol, and ECM derived from decellularized renal tissues. This bioink facilitated the creation of an in vitro kidney model containing primary kidney cells. The printed structures demonstrated high cellular activity and replicated the structural and functional features of native kidney tissue, providing a promising platform for studying kidney physiology and disease. The intricate vascular system of the kidney is critical for its function, as it supports filtration and nutrient exchange. Chae et al. employed vascular corrosion casting to develop a biomimetic renal vascular scaffold. This scaffold effectively supported blood vessel formation, a crucial step toward creating functional kidney constructs. Additionally, Lin et al. advanced the field by utilizing a gelatin‐fibronectin bioink to print vascularized proximal tubules. By co‐culturing proximal tubular cells with ECs, their model achieved active glucose and albumin reabsorption, demonstrating potential for disease modeling and pharmacological studies. Proximal tubules are central to kidney function, reabsorbing 65%–80% of nutrients from the glomerular filtrate. Dysfunction in these tubules can lead to conditions such as renal Fanconi syndrome. Kimberly A. Homan and colleagues developed 3D‐printed artificial proximal tubules composed of epithelial cells encasing open luminal spaces. These structures exhibited normal physiological functions, enabling drug metabolism studies and complementing extracorporeal dialysis. Similarly, Addario et al. created biomimetic proximal tubular structures using alginate‐based bioinks. Their constructs, embedded with primary mouse renal tubules, ECs, and fibroblasts, maintained function and metabolic activity for nearly a month. The glomerulus and tubular structures are essential for kidney function. The glomerulus, comprising podocytes, ECs, and mesangial cells, is responsible for blood filtration, while the tubules manage reabsorption and secretion. Homan et al. and Zhang et al. have made significant strides in replicating the glomerular filtration barrier's intricate architecture. Using advanced bioprinting techniques, they optimized the biomechanical properties of these constructs to ensure effective filtration. Efforts to bioprint tubular structures have also focused on ensuring functional and anatomical accuracy. The use of patient‐derived cells, as demonstrated by Yu et al., enhances biocompatibility and reduces the risk of immune rejection. Their vascularized renal tissues highlight the feasibility of engineering complex renal structures for transplantation and regenerative medicine.^116^ The nephron, the kidney's functional unit, integrates glomerular and tubular components to perform filtration and reabsorption. Researchers like Jia et al. have achieved significant progress in bioprinting functional nephrons, replicating their intricate architecture and filtration capabilities. However, challenges remain in achieving the precise cellular composition and ensuring long‐term functionality and maturation in vivo. Bioprinting technology has evolved from static models to dynamic, stimuli‐responsive systems. These advanced designs mimic the kidney's response to physiological changes, offering platforms for disease modeling, drug testing, and personalized medicine. Kidney‐on‐chip devices, such as those developed by Homan et al., provide perfusable 3D models of the proximal tubule. These devices enable studies on nephrotoxicity, epithelial–interstitial interactions, and renal diseases.^115^ By incorporating fugitive inks and ECM layers, researchers created tubular channels seeded with proximal tubular epithelial cells, achieving polarization and functionality over time. Despite remarkable advancements, several challenges persist in kidney bioprinting. Achieving the nephron's intricate architecture and ensuring the long‐term functionality of bioprinted kidneys are formidable tasks. Immunocompatibility and the risk of rejection remain concerns, necessitating further innovation in materials science and cell biology. Collaboration among multidisciplinary teams is essential to overcome these obstacles. Integrating stem cell differentiation techniques, optimizing vascularization strategies, and leveraging computational modeling will accelerate progress toward fully bioengineered kidneys. With continued efforts, bioprinting holds the potential to revolutionize organ transplantation, offering life‐saving solutions for patients with end‐stage renal disease and other kidney‐related conditions.^117^

#### 5.1.10 Pancreas bioprinting

The pancreas is a complex and multifunctional organ, essential for both digestion and glucose regulation. Structurally, it is composed of two main functional regions: the exocrine portion, responsible for producing digestive enzymes, and the endocrine region, which houses the islets of Langerhans. These islets are clusters of hormone‐secreting cells that play a critical role in maintaining glucose homeostasis. Given the complexity and importance of the pancreas, its regeneration and repair through bioprinting technologies have become an exciting frontier in regenerative medicine. Bioprinting the pancreas is still in its nascent stages, primarily focusing on islet encapsulation for treating type 1 diabetes (T1D) and creating pancreatic cancer models. T1D is a chronic condition marked by the autoimmune destruction of insulin‐producing β cells in the pancreas, leading to disrupted glucose regulation. One promising approach to managing T1D is through islet transplantation. However, this method faces numerous challenges, including a limited supply of donor islets, immune rejection, islet loss post‐transplantation, and the risk of hypoxia due to inadequate oxygen supply. Recent advances in bioprinting technologies aim to address these issues by creating engineered environments that enhance islet viability and function. For example, Farina et al. developed a vascularized and porous encapsulation device to shield transplanted islets from hypoxia. Such innovations improve oxygen diffusion to the islets, reducing graft failure rates. To overcome the shortage of donor islets, researchers are exploring the generation of insulin‐producing β cells from HiPSCs. This approach holds great promise as it circumvents^118^ the reliance on donors while enabling the production of patient‐specific cells. Song et al. demonstrated^119^ the potential of bioprinting macroporous devices to house iPSC‐derived β cells, emphasizing the critical role of the microenvironment in cell differentiation and maturation.^120^ Similarly, Kim et al. developed a bioink formulation using pancreatic decellularized extracellular matrix (pdECM), which mimics the native pancreatic environment and enhances insulin secretion in iPSC‐derived cells. By encapsulating these engineered islet‐like aggregates in a porous PCL shell, researchers created a hybrid macro‐encapsulation system suitable for subcutaneous transplantation. Klak et al. further advanced this approach by bioprinting functional pancreatic tissues using pdECM‐based bioink. These bioprinted tissues demonstrated biocompatibility and the formation of islet‐like structures in vivo, marking a significant step toward creating functional pancreatic replacements. Despite these advancements, bioprinting functional pancreatic tissues poses unique challenges. The high shear forces during the printing process can compromise cell viability and functionality. Alternative bioprinting techniques, such as SLA, have been explored to address these issues. Wang et al., for instance, used SLA to construct pancreatic tissues with high resolution, demonstrating its potential for creating precise and functional structures. In addition to addressing T1D, bioprinting technologies are making significant contributions to pancreatic cancer research. Pancreatic ductal adenocarcinoma (PDAC), a highly aggressive and lethal form of cancer, requires innovative models for studying tumor biology and testing potential treatments. Hakobyan et al. utilized LAB to create exocrine spheroids composed of acinar and ductal cells, providing a 3D model for investigating PDAC^121^ development. Similarly, Huang et al. designed models to simulate tumor–stroma interactions, enabling researchers to study the complex microenvironment of pancreatic tumors and identify effective therapeutic strategies. The ultimate goal of pancreatic bioprinting is to replicate the organ's native anatomy and functionality, encompassing both its endocrine and exocrine regions. While significant progress has been made in islet replacement strategies for T1D, other aspects of pancreatic TE remain underexplored. Developing bioprinted pancreatic tissues for in vitro disease modeling and as a source of transplantable tissues could revolutionize the treatment of pancreatic diseases. Additionally, integrating advanced vascularization techniques into bioprinted tissues could address oxygenation challenges, enhancing the viability and longevity of engineered pancreatic tissues. Innovations in bioink formulations, such as those based on pdECM, and the development of alternative bioprinting methods will further advance the field.^79^

#### 5.1.11 Bladder bioprinting

Total bladder substitution is necessary for patients who have undergone cystectomy due to bladder cancer, trauma, infection, inflammation, iatrogenic injury, or interstitial cystitis, all of which can lead to significant bladder damage. However, challenges associated with artificial bladders include the deposition of calcareous materials, urine leakage leading to peritonitis, encrustation, and infections. Therefore, an ideal artificial bladder should be made from an inert material that is non‐irritating and non‐allergic. One promising approach for bladder reconstruction is the use of prosthetic bladders, with bioprinting offering significant potential in this regard. Despite the limited number of studies focusing on bladder bioprinting, the technology has shown considerable promise for bladder regeneration. Xu and colleagues developed a cell‐encapsulating droplet generation system for printing building blocks within a controlled sterile environment.^122^ These building blocks assemble into a SMC patch, which was cultured for 51 days to form a 3D tissue construct resembling the native rat bladder. More recently, Zhang et al. employed integrated bioprinting technology to fabricate a urethra using a blend of PCL and poly(lactide‐co‐caprolactone) (PLCL) thermoplastic polymers.^123^ These polymers were combined with a HG bioink composed of fibrin, gelatin, and HA, and seeded with urothelial cells (UCs) and SMCs to mimic the natural urethra of rabbits. By printing a tubular scaffold and simultaneously delivering bioink, they successfully formed the urethral structure. Imamura and colleagues took a different approach, using a 3D bioprinting robot system to reconstruct urinary bladders. This biofabricated bladder included bone marrow‐derived cells, which differentiated into SMCs to contribute to the bladder's structure. While these developments are promising, further work is necessary to meet the ideal requirements for the fabrication of functional artificial bladders. The use of inert biomaterials in bioprinting approaches may offer solutions to challenges like calcareous deposits.

#### 5.1.12 Small intestine bioprinting

Tissue‐engineered small intestine (TESI) represents a potential autologous therapy that addresses the challenges of donor graft shortages and the need for long‐term immunosuppression in intestinal transplantation. First introduced by Vacanti et al., TESI is created by implanting organoid units—multicellular clusters of epithelium and mesenchyme derived from the native intestine—into the host. These organoids are placed on a scaffold and implanted into the omentum, leading to the formation of TESI. The resulting tissue closely mimics the native intestine's histology, containing all four epithelial cell types along with lamina propria, nerve components, muscularis mucosa, and enteric neuronal plexuses. However, it lacks the proper alignment of circular and longitudinal smooth muscles, which are essential for generating the force and motility needed for nutrient absorption.^124^

#### 5.1.13 Ovary bioprinting

A precursory human ovary has been developed with self‐assembled microtissues created using novel 3D petri dish technology with the intention of studying in vitro maturation of immature oocytes and the development of a system to study the effect of environmental toxins on folliculogenesis. Researchers in the United States have bioengineered an artificial ovary that makes sex hormones in the same proportions as a healthy one. The bioengineered ovary shows sustained release of sex hormones, estrogen, and progesterone in vitro. Such bioengineered ovaries may provide a more natural option for women than hormone replacement therapy.^125^

#### 5.1.14 Thymus bioprinting

Researchers have successfully rejuvenated an aged thymus that had undergone complete involution. The rejuvenated thymus displayed structural and gene expression characteristics similar to those of a young thymus. This study demonstrates that activating a single transcription factor can significantly reverse the age‐related shrinkage of the thymus, offering potential implications for regenerative medicine.^126^

#### 5.1.15 Dentistry bioprinting

Recent advancements in regenerative medicine have made significant strides, and dentistry is not lagging behind. Regenerative Dentistry offers numerous clinical benefits, such as techniques for repairing and restoring teeth damaged by caries, addressing periodontal defects, and advanced grafting procedures for the maxilla and mandible. Much of the research focuses on dentin regeneration, pulp regeneration, periodontal regeneration, restoring resorbed roots, and repairing root perforations. Additionally, TE methods are being explored to promote healing of oral wounds and ulcers, while gene‐transfer techniques aim to alter salivary proteins and oral microbial colonization patterns. Wei et al. have successfully regenerated a functional bio‐root structure using allogenic dental stem cells for artificial crown restoration.^127^ Studies have also demonstrated the successful transplantation of bioengineered tooth germs into the alveolar bone in mice, leading to a functioning tooth. Further research is exploring the potential of dental stem cells from American alligators to regenerate teeth in humans. Nondental stem cells, such as those derived from urine, have also been studied for their potential in dental applications. Whole tooth regeneration, which could replace traditional dental implants, is another area under investigation. In endodontics, regenerative approaches such as root canal revascularization, stem cell therapy, scaffold implantation, injectable scaffold delivery, pulp implantation, 3D cell printing, and gene therapy are being explored. Of these, root canal revascularization is currently the only clinically viable method, while others remain within the research phase. As new discoveries and innovative techniques continue to emerge, regenerative therapies hold great promise in revolutionizing the field of dentistry.^128^

#### 5.1.16 Retina bioprinting

The retina, which is responsible for sensory information perception, is a complex nervous tissue containing rod and cone photoreceptors. Human retina exhibits immense structural complexity and is highly vascularized, consisting of various cell types, including horizontal cells, amacrine cells, retinal pigment epithelial (RPE) cells, retinal ganglion cells (RGCs), bipolar cells, glial cells, and Müller cells. There are 55 different cell types and 105 photoreceptor cells per square millimeter in the retina. For proper functioning, these retinal cells must work in harmony to transmit visual information to the brain. The RPE is the outermost layer of the retina and consists of a single layer of hexagonal, pigmented, polarized epithelial cells. These RPE cells play a critical role in the visual process, and their malfunction can lead to retinal diseases. The choroid layer provides the vascular structure needed to supply nutrients and oxygen to other retinal layers. Between the RPE and choroid is the Bruch's membrane. The RGCs reside in the innermost portion of the retina, while photoreceptors are located in the outermost region. When light strikes the retina, it activates the photoreceptors. The photons are absorbed by the visual pigments in the photoreceptors, which are then translated into electrical signals. These electrical signals stimulate the neurons of the retina, and the signals are transmitted to the brain through the spike discharge patterns of the ganglion cells. To ensure proper vascularization, blood vessels pass through the retina. In certain retinal diseases, specific cells need to be replaced, such as RGCs in glaucoma. In other cases, larger areas of the retina need to be restored. The retina deteriorates with age or due to conditions like diabetes or vascular occlusion and fails to regenerate, increasing the need for cell transplantation and 3D bioprinting technologies. While cell transplantation has limitations, such as axon orientation in vivo, bioprinting a complex retina with multiple cell types remains a significant challenge. The scaffold used for retinal TE must be porous, biocompatible, not induce a foreign body response, mechanically stable, and thin. Advances in stem cell biology have enabled the development of retinal equivalent constructs through bioprinting, using stem cells that play essential roles in retinal TE, such as embryonic stem cells (ESC) and iPSCs. The process of retinal reconstruction can be achieved by leveraging the differentiation potential of iPSCs, which can be directed to develop into various retinal cell types, including RPE cells and RGCs. Retinal TE includes the fabrication of key components such as Bruch's membrane, RPE cells, retinal glial cells, and RGCs. In this context, bioprinted scaffolds act as essential substrates to support the attachment and growth of different retinal cell types. Among these retinal cells, RGCs are situated in the ganglion cell layer and are responsible for transmitting visual information from photoreceptors to the brain. However, RGCs are unable to regenerate naturally. When developing RGCs, critical considerations include maintaining proper axon orientation, accurate cell positioning, and ensuring cell survival. These challenges can be overcome by using scaffolds that provide the necessary physical support. The scaffold must be capable of guiding axon growth, promoting cell adhesion, differentiation, proliferation, and migration. Kador et al. demonstrated the use of thermal inkjet 3D bioprinting to place RGCs precisely on electrospun scaffold surfaces, effectively guiding the outgrowth of RGC neurites.^129^ In another study, Lorber et al. evaluated the impact of inkjet printing on RGCs and glial cells and found that although there was a decrease in cell population, the survival rate of the cells remained unaffected during the printing process.^130^ Bruch's membrane, a specialized and thin (2–4 μm) barrier between the choroid and retina, plays a crucial role in retinal TE as it serves as a scaffold for cellular growth. Its essential features include being ultrathin, porous (to control diffusion rates), and permeable (to support nutrient exchange). To address limitations related to pore‐size distribution in Bruch's membrane, Tan et al. developed an ultrathin PCL membrane with regular, interconnected pores, which replaced the thicker, less regular pores reported in previous studies.^131^ This low‐cytotoxic membrane showed improved arrangement of ARPE‐19 cells, forming a functional barrier that supports homeostasis. Further extending the use of these ultrathin membranes, Shi et al. used a microvalve‐based bioprinting technique to create a retinal model with ARPE‐19 cells seeded on the membrane. This model included a monolayer of RPE and Bruch's membrane, onto which Y79 cells were printed within an alginate/pluronic bioink.^116^

#### 5.1.17 Cornea bioprinting

**FIGURE 8:**
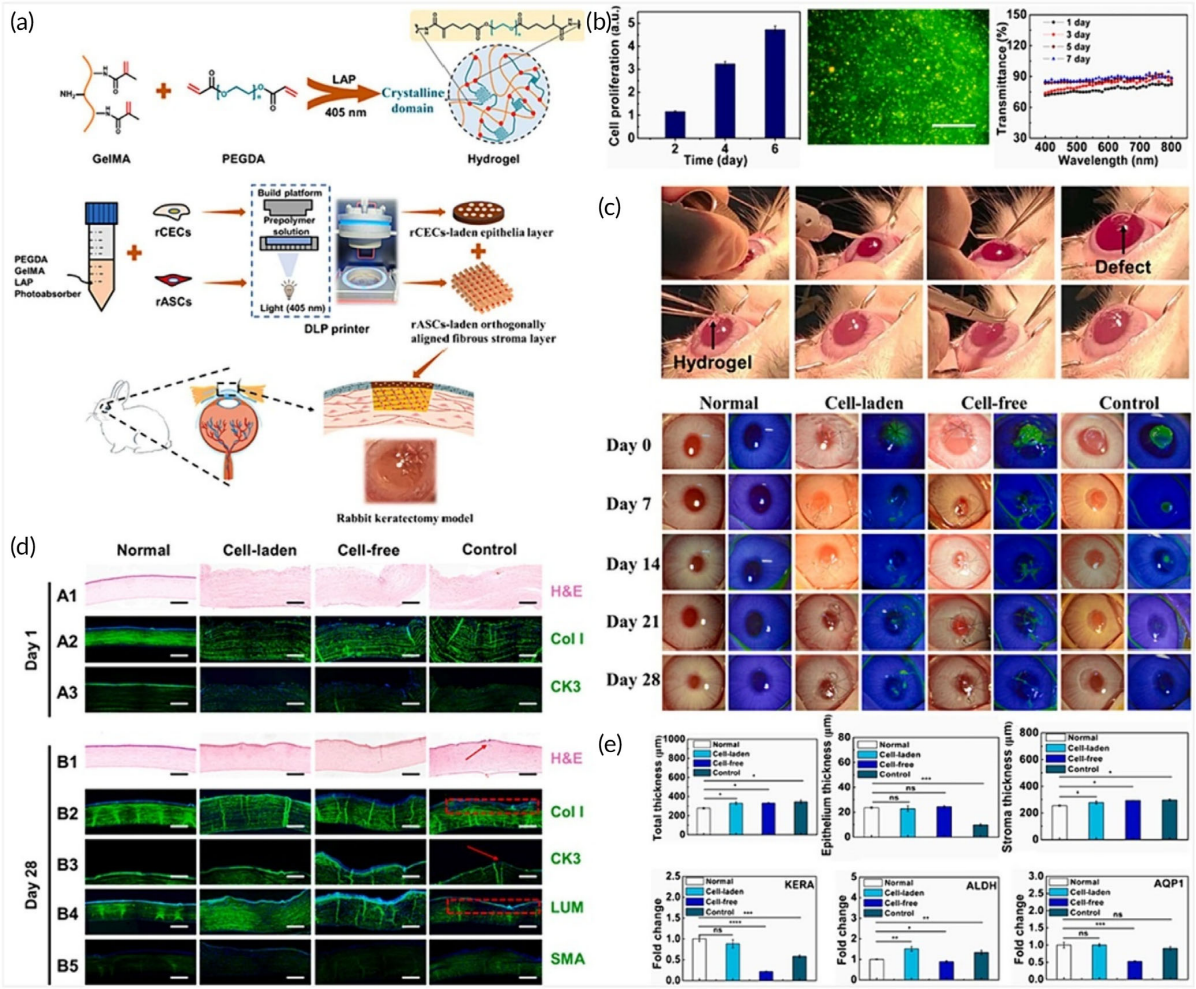
Schematic summary of 3D bioprinted PEGA‐GeIMA (Poly(ethylene glycol) acrylate ‐ Gelatin methacryloyl) hydrogel scaffolds for corneal tissue engineering and regeneration. (a) Schematic of PEGA‐gelatin methacryloyl (GelMA) hydrogel formation and bioprinting procedure, illustrating fabrication of corneal scaffolds. (b) rCEC‐laden (rabbit Corneal Epithelial Cells) hydrogels show high cell proliferation, live/dead staining confirms cell viability, and gradual transparency change indicates scaffold maturation. (c) In vivo rabbit model demonstrates gradual closure of corneal epithelial defects, indicating effective epithelial migration on the hydrogel scaffold. (d) Histological and gene expression analyses reveal restoration of epithelium and stroma; unfinished regions are highlighted by red arrows/rectangles. (e) Quantification shows thickness recovery of entire cornea, epithelium, and stroma, with increased expression of corneal markers KERA, ALDH, and AQP1 relative to normal corneas, confirming functional regeneration reproduced with from Mirshafiei et al.^132^ DLP, digital light processing; PEGDA, polyethylene glycol diacrylate. A value of *p* < 0.05 was considered statistically significant, and ns, *, **, ***, **** represent *p* > 0.05, *p* < 0.05, *p* < 0.01, *p* < 0.001, *p* < 0.0001, respectively.

The cornea, being transparent, avascular, and highly organized, plays a critical role in protecting the eye and refracting light to the retina. Its specialized structure consists of 200–250 layers of collagen fibers, arranged in a specific pattern that provides strength and contributes to light transmission. Corneal diseases are a leading cause of blindness worldwide, and although corneal transplantation is the most common treatment, there remains a severe shortage of donor corneas. To address this issue, research into 3D bioprinting for corneal replacement has gained momentum, promising precise control over corneal curvature and thickness for individual patients. A major challenge in developing bioprinted corneal materials is ensuring rapid and stable epithelialization. Research has shown that the curvature of corneal implants can influence cell adhesion and organization. The use of finite element modeling (FEM) confirmed that the curved implants promoted greater cell‐substrate adhesion and quicker epithelialization, leading to improved corneal regeneration in vivo. Kim et al. studied shear forces during the 3D printing process and found that the shear stress influenced the orientation of collagen fibrils in the cornea, which is essential for maintaining the natural lattice pattern of the tissue and promoting collagen assembly. This research has paved the way for designing bioprinted corneal grafts with structures resembling native human corneas, both in optical properties and collagen arrangement. Furthermore, bioprinted corneal models have shown promise in mimicking natural corneal tissue. Duarte Campos et al. demonstrated the fabrication of 3D corneal models using a drop‐on‐demand technique, which replicated the optical characteristics and cellular behavior of native corneas. Additionally, studies using DLP for corneal curvature modeling have achieved significant success in printing bilayer structures for corneal regeneration. For example, He et al. created a bilayer implant composed of an epithelial layer with corneal epithelial cells and a fibrous stromal layer with MSCs. This bilayered approach, when tested in a rabbit model, resulted in successful corneal healing, demonstrating the effectiveness of precise cellular arrangement in promoting regeneration (Figure 8).^117^

#### 5.1.18 Ear bioprinting

**FIGURE 9:**
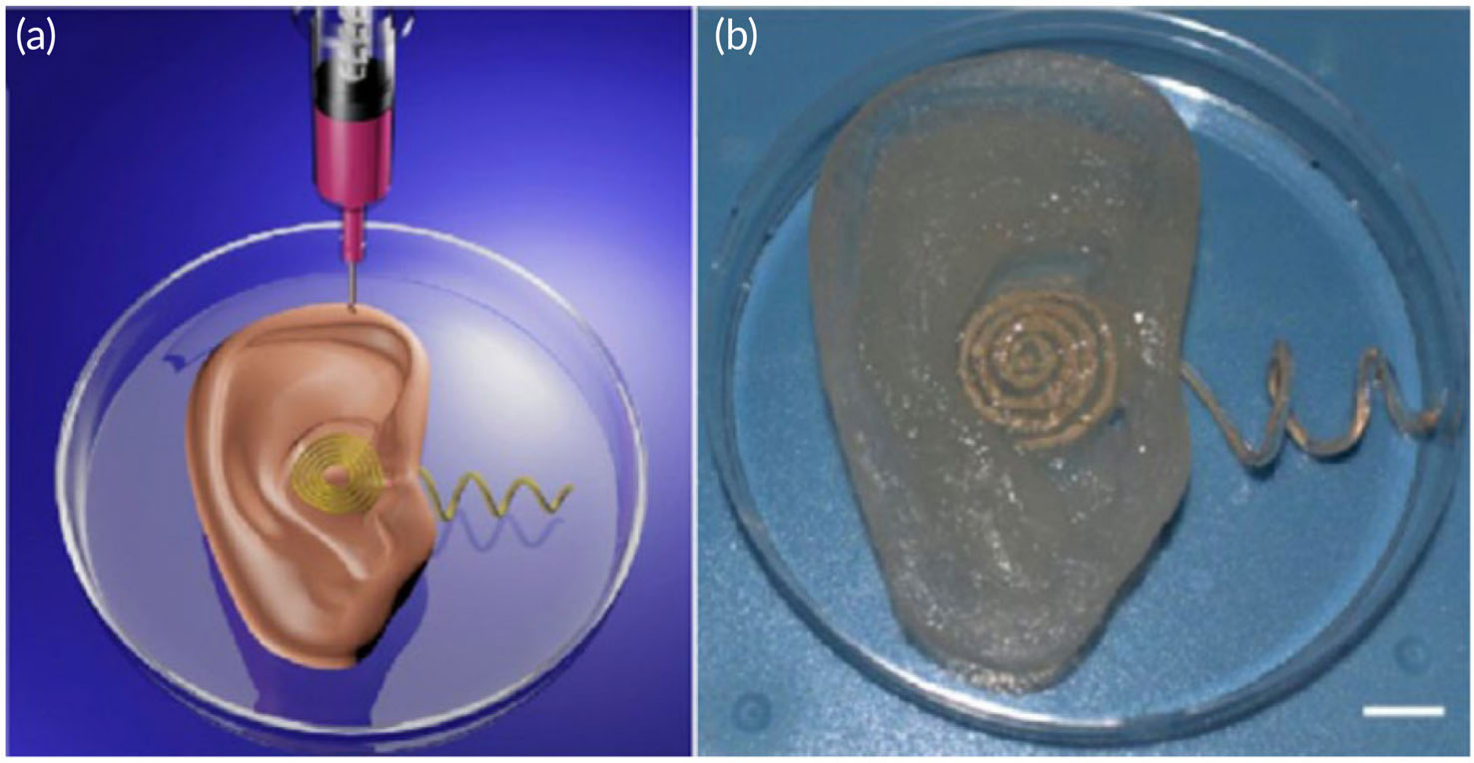
Schematic summary of a 3D‐printed bionic ear integrating cartilage tissue engineering with functional electronics. (a) an anatomical ear structure was three‐dimensional printed using a conductive polymer composed of cell‐laden hydrogel and silver nanoparticles, allowing for the culturing of cartilage tissue. (b) The printed ear successfully demonstrated auditory functions such as radio frequency reception and stereophonic music listening by sensing inductively coupled signals with integrated electronics.^133^

The New York Times highlighted a significant achievement by the U.S.‐based regenerative medicine company, 3DBio Therapeutics. The company successfully conducted the first implantation of “AuriNovo,” a 3D‐printed ear implant developed from human cells to reconstruct a patient's missing outer ear. In 2018, a collaboration involving the Ninth People's Hospital Affiliated with Shanghai Jiao Tong University School of Medicine, the Shanghai Key Laboratory of Tissue Research, the China National Center for Tissue Engineering, Weifang Medical University, and Xinhua Hospital of Dalian University yielded remarkable results. They used chondrocyte culture and 3D printing techniques to create “ear implants,” which were successfully implanted in five children, aged 6–9 years, who suffered from unilateral microtia. While tissue‐engineered ear cartilage technologies have seen significant advancements, clinical applications still face notable challenges. A key issue is enhancing the mechanical strength and wear resistance of ear cartilage to ensure its long‐term shape retention. Moreover, it is vital to balance the regeneration and degradation rates of chondrocytes within the body to prevent excessive breakdown that could result in structural instability. 3D bioprinting stands out for its capability to create biological tissues with functional characteristics by simultaneously integrating cells and functional materials. For example, Mannoor et al. developed bioengineered ears embedded with functional electronics. Using alginate HGs seeded with chondrocytes, they printed structures in the shape of human ears. Additionally, a conductive silicone infused with silver nanoparticles was incorporated into a coil antenna, which was connected to cochlea‐like electrodes and supported by silicone. The engineered ears demonstrated exceptional stability during in vitro cell culture, with cartilage forming around the antenna, resulting in highly viable tissue structures with excellent morphology. Beyond this, these printed ears exhibited remarkable functionality—they were capable of receiving electromagnetic signals and even playing stereo audio music (Figure 9).^100^

### 5.2 Bone, muscle, and skin regeneration: innovative treatments with bioprinting

#### 5.2.1 Skin and wound healing

3D bioprinting has emerged as a transformative technology in regenerative medicine, particularly in skin regeneration, providing solutions for wound healing, burn treatment, and reconstructive surgeries. By enabling the creation of highly accurate, patient‐specific skin grafts and models, this technology addresses critical limitations in traditional treatments. Interdisciplinary collaborations between researchers, clinicians, and industry stakeholders have significantly accelerated advancements in this field.

1. Wound healing and burn treatment
  3D bioprinting is revolutionizing the treatment of severe burn injuries and chronic wounds by enabling the production of customized skin grafts that reduce immune rejection risks and donor dependency. For instance, Michael et al. demonstrated the effectiveness of bioprinted skin constructs containing keratinocytes and fibroblasts in enhancing wound closure rates and promoting collagen deposition in preclinical models. Similarly, Singh et al. introduced a nanocomposite bioink integrating graphene oxide, which significantly improved the antibacterial properties of bioprinted constructs for diabetic wound healing.
2. FTSs constructs
  The development of FTSs equivalents comprising the epidermis, dermis, and vascular networks represents a critical advancement. Liu et al. successfully printed vascularized constructs integrating human dermal fibroblasts and ECs, showing promising results in achieving functional integration with host tissues. These models are invaluable not only for clinical grafting but also as pharmaceutical testing platforms. For example, Zhao et al. utilized bioprinted skin models for evaluating the safety and efficacy of topical formulations, reducing the reliance on animal testing.
3. Skin‐on‐a‐chip models
  The integration of bioprinting and microfluidic technologies has facilitated the development of skin‐on‐a‐chip systems for drug and cosmetic testing. Zhang et al. engineered a bioprinted skin‐on‐a‐chip platform embedded with immune cells to study inflammatory responses, providing a robust tool for high‐throughput screening. These systems are particularly valued by pharmaceutical companies aiming to accelerate product development cycles.
4. Reconstructive surgery and esthetic medicine
  In reconstructive surgeries, 3D bioprinting enables the creation of patient‐specific implants with precise pigmentation and texture. Kang et al. developed personalized bioprinted grafts for facial reconstruction using imaging‐guided design, achieving both esthetic and functional restoration. Furthermore, interdisciplinary teams are exploring bioinks enriched with melanocytes to match patients' natural skin tones, as demonstrated by Gupta et al. in their work on pigmentation‐controlled constructs for burn recovery.

Additionally, in situ bioprinting, where skin layers are directly printed onto injured areas, has gained traction. He et al.^134^ successfully utilized this technique to treat large‐area burns in preclinical studies, demonstrating significant improvements in healing rates and reduced infection risks. The advancements in skin bioprinting are made possible by robust interdisciplinary collaborations:

1. Academic and industry collaborations: The Wyss Institute at Harvard University partnered with Organovo to optimize scalable bioprinting platforms for skin regeneration.
2. European Union Initiatives: Projects like “SkinPrint” have brought together researchers from multiple disciplines to overcome challenges in vascularization and scaling.
3. Clinician‐led innovations: Surgeons collaborating with bioprinting teams, such as those in Min et al.,^135^ have integrated imaging technologies like 3D scanning to design patient‐specific constructs tailored for reconstructive procedures.^135^

Despite its potential, 3D bioprinting for skin regeneration faces challenges in scaling, regulatory approvals, and vascularization. Collaborative efforts continue to address these barriers. For instance, biofabrication teams are exploring nanomaterial‐functionalized bioinks, while artificial intelligence (AI)‐driven bioprinting systems are being tested to improve precision and reproducibility. The field of 3D bioprinting for skin regeneration has reached a pivotal stage, supported by an ecosystem of researchers, clinicians, and industry experts. Its applications in wound healing, drug testing, and reconstructive surgery underscore its transformative potential, paving the way for broader adoption in clinical and commercial settings. Bioprinting technology has made remarkable advancements in the creation of skin tissues. In one study, a jet‐based bioprinter was employed to fabricate multilayered, biomimetic skin tissue using human keratinocytes and fibroblasts. The method involved separately printing collagen HG and cells, followed by cross‐linking collagen embedded with the cells. This approach successfully produced distinct dermal and epidermal‐like layers by simultaneously printing the two cell types. Furthermore, an innovative in situ skin printer was developed to apply cells directly onto the body for treating severe burn injuries. This device was utilized to repair full‐thickness wounds in pigs by delivering keratinocytes and fibroblasts directly to the wound site. The treatment resulted in rapid epithelialization and significantly improved wound healing, demonstrating the printer's potential in regenerative medicine.^136^

#### 5.2.2 Sweat glands bioprinting

**FIGURE 10:**
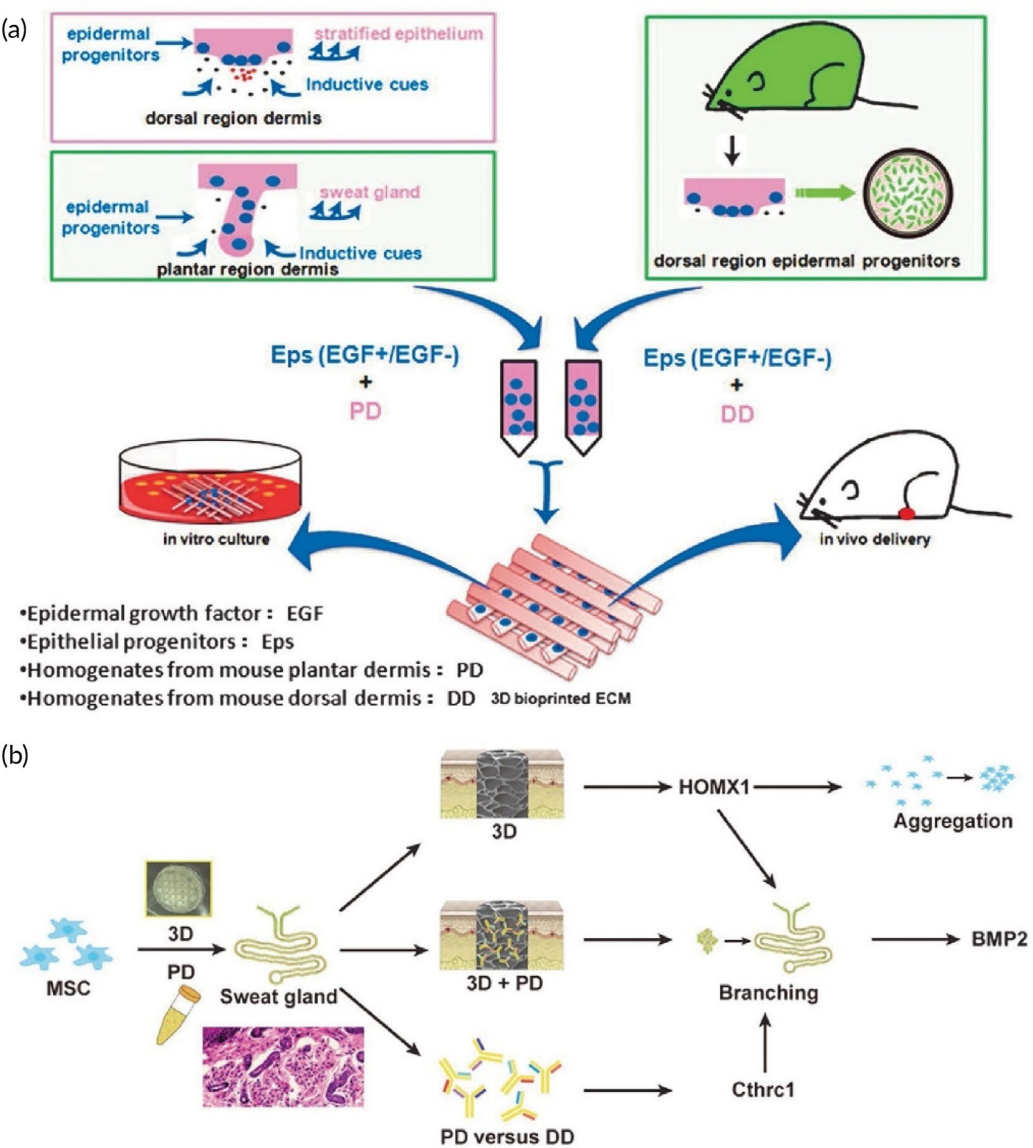
Schematic summary of a 3D‐bioprinted in vitro microenvironment for sweat gland regeneration. In vitro bioprinted microenvironment for sweat gland regeneration is shown (a) three‐dimensional (3D) bioprinting of composite hydrogels containing epidermal progenitor cells and extracellular matrix (ECM) successfully mimics the in vitro microenvironment for sweat gland regeneration, and.^137^ (b) 3D‐bioprinted matrix initiates sweat gland branching morphogenesis by directing mesenchymal stem cells (MSC) differentiation through CTHRC1 biochemical signals and structural cues.^138^

Skin appendages, including sweat glands, hair follicles, and sebaceous glands, are essential for maintaining skin barrier function and regulating body temperature. Studies have shown that the microenvironment plays a crucial role in the regulation and differentiation of these appendages. Various bioengineering techniques involving stem cells or spheroids have demonstrated potential in promoting skin tissue regeneration for wound healing, with 3D bioprinting playing a significant role in these advancements. Sweat glands, which include eccrine and apocrine types, play a crucial role in thermoregulation, with eccrine glands being responsible for sweat production. Damage to these glands caused by trauma, such as deep burns, often leads to a loss of sweat function, preventing the body from properly dissipating heat. Based on these findings, the study suggested that the bioprinted scaffolds could be used for regenerating sebaceous glands, particularly in the context of tarsal plate regeneration.^117^ Sweat glands are classified into eccrine and apocrine glands, with eccrine glands playing a primary role in thermoregulation and perspiration. Individuals with extensive trauma or deep burns often experience impaired sweat gland regeneration, resulting in difficulties with sweating and heat dissipation in warm conditions. In efforts to reconstruct sweat glands in vitro, Huang et al. pioneered the use of 3D bioprinting with ECM containing mouse plantar dermis (PD) homogenate. This approach created a conducive environment for the differentiation of mouse epidermal progenitor cells into sweat glands. Transplanting these bioprinted constructs into burned mouse paws restored sweat gland functionality (Figure 10a).^137^ Similarly, Yao et al. utilized extrusion‐based bioprinting to develop a sweat gland‐like microenvironment in vitro, promoting the differentiation of MSCs into functional sweat glands (Figure 10b).^138^ Their findings highlighted the significance of both biochemical and structural cues provided by the 3D‐printed matrix in guiding MSCs toward a glandular lineage. Additionally, the study identified Collagen Triple Helix Repeat Containing 1 (CTHRC1) as a key biochemical factor for sweat gland differentiation and heme oxygenase 1 (Hmox1) as a crucial gene involved in MSC differentiation and biomechanical activation, both contributing to the expression of sweat gland‐specific genes. Song et al. further reviewed advancements in bioprinting‐assisted sweat gland regeneration, emphasizing the importance of biomimetic microenvironments in supporting sweat gland reconstruction.^139^

#### 5.2.3 Hair follicles bioprinting

Hair follicles, which exist within the dermal layer of the skin, are composed of key components such as the hair papilla, hair matrix, root sheath, and hair bulges. The papilla is particularly important due to its influence on hair length, strength, and growth cycles. Wu et al. conducted an experiment in which hair follicle epithelial cells, dermal papilla cells (DPCs), and dermal sheath cells were combined with collagen and transplanted into the dorsal skin of nude mice, resulting in the formation of hair fibers.^140^ However, culturing human hair follicle cells (HPCs) in vitro often leads to a loss of their induction ability. To address this, Higgins et al. used a 3D spheroid culture to partially maintain the transcriptional signature of DPCs, which, when transplanted in situ, were able to induce hair follicles in human skin. Similarly, Miao et al. prepared 3D HPC spheres based on a Matrigel scaffold, which helped preserve essential markers of hair papilla cells, such as NCAM and α‐SMA, and maintained the cells' inductive properties in a 3D culture environment.^139^

#### 5.2.4 Muscle bioprinting

**FIGURE 11:**
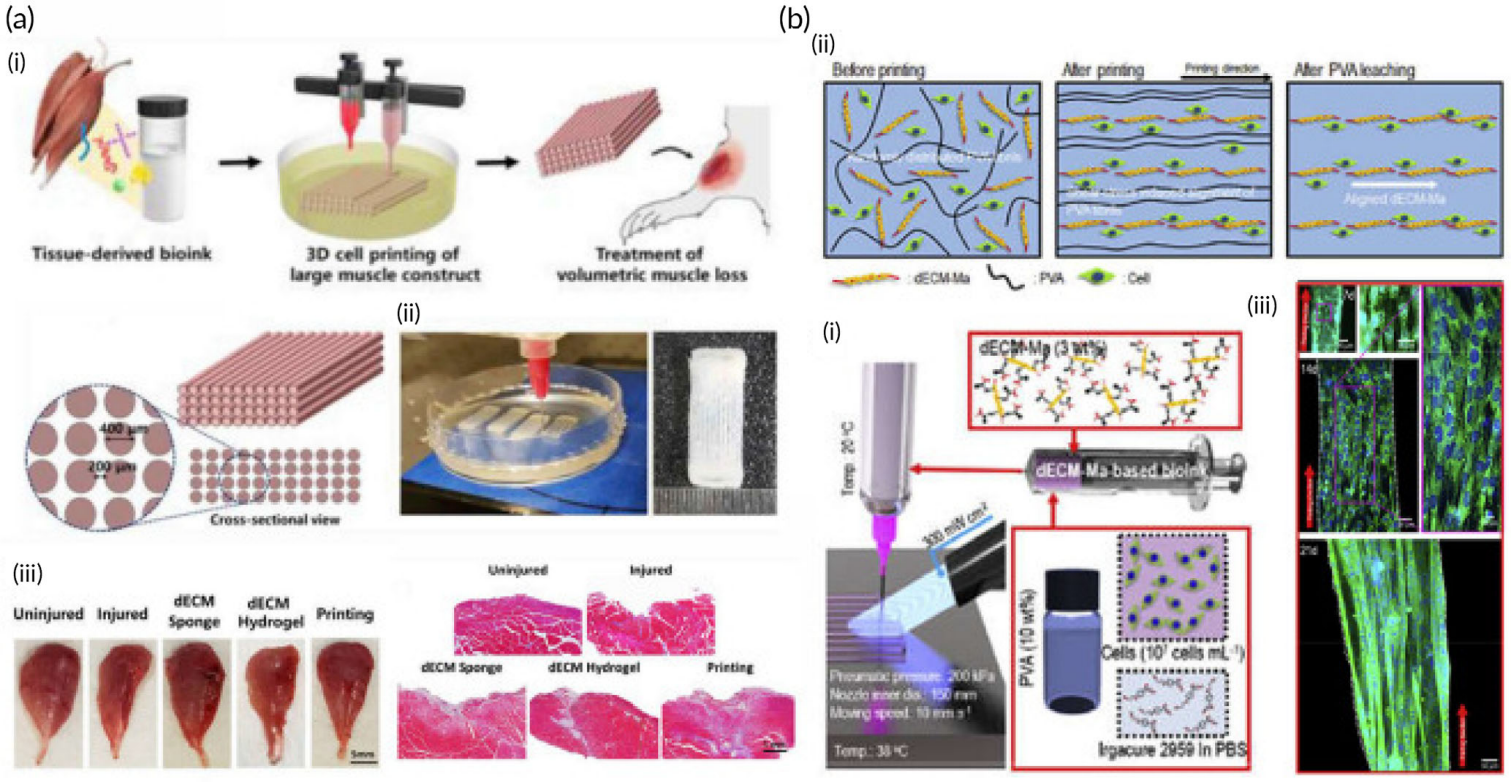
Schematic summary of 3D‐bioprinting strategies for promoting muscle regenerations. (a) Cell‐laden three‐dimensional (3D) bioprinting with granule‐bath bioink structurally and histologically promotes muscle regeneration in a muscle‐wasting model.^143^ (b) 3D bioprinted aligned constructs using dECMMMA‐based bioink and ultraviolet (UV) cross‐linking enable organized myofiber formation and functional muscle regeneration in C2C12 cells.^144^ dECM, decellularized extracellular matrix; PVA, polyvinyl alcohol.

Biological microelectromechanical systems (Bio‐MEMS) that integrate biological components hold great promise for advancing bioengineering applications, including the development of motors, actuators, heart pumps, and biosensors. Muscle cells, in particular, are frequently utilized in these systems due to their ability to generate force through actin‐myosin motors, which are regulated by excitation‐contraction coupling. These muscle‐powered devices, which harness energy from biochemical reactions, offer notable advantages such as high energy efficiency, resource conservation, and compact design. C_2_C_12_ skeletal muscle cells, known for their capacity to proliferate and differentiate into multinucleated myotubes, are commonly used in these applications. This well‐characterized cell line closely mimics in vivo skeletal muscle tissue when cultured and differentiated in vitro. Although C_2_C_12_ cells have proven useful for Bio‐MEMS, their precise integration into microdevices is essential to ensure reliable and consistent performance.^141^ Conventional Bio‐MEMS fabrication methods often involve manually seeding cells onto the devices, a process that can lead to uneven cell distribution, affecting both cell growth and differentiation. Therefore, a more precise technique for seeding cells is critical to improving the consistency and reliability of these systems. Recent developments in bioprinting have enabled the accurate placement and alignment of C_2_C_12_ cells on microdevice components, such as cantilevers, with a resolution of 300 dpi (approximately 85 μm). In this process, an equal number of cells were printed onto each cantilever to ensure uniform coverage, which allowed for controlled cell proliferation and differentiation with minimal variation. The printed C_2_C_12_ cells exhibited a viability of 91.2 ± 2.6%, and they successfully aligned to form confluent myotubes on nearly all cantilevers. These myotube‐cantilever constructs responded synchronously to electric pulses of 2 V for 40 ms at frequencies of up to 5 Hz, demonstrating that bioprinted microdevices can replicate, and even surpass, the physiological properties of traditionally fabricated devices while significantly reducing culture time. Additionally, the bioprinted myotubes are suitable for muscle exercise studies with varying electric stimulation frequencies, further showcasing the versatility of this bioprinting approach. Severe muscle injuries, such as those involving more than 20% muscle loss, are often irreparable and necessitate effective regenerative therapies. The current standard treatment, autologous tissue transfer, is limited by damage to the donor site and often fails to achieve complete functional regeneration. As a result, the development of engineered implants that can enhance muscle regeneration has become a promising strategy. A key focus in this field is creating a biomimetic microenvironment that mimics the biochemical and topographical features necessary for muscle repair. dECM‐based scaffolds are widely used in muscle regeneration because they provide an ideal biochemical environment, containing essential structural proteins like collagen, glycosaminoglycans, and growth factors that support cell growth and differentiation.^142^ However, it has been shown that random cell alignment within these scaffolds can lead to disorganized muscle fibers and scar tissue formation. To overcome this, scaffolds must include topographical cues that guide the alignment of myotubes, which is critical for functional muscle regeneration. At the macroscopic level, 3D extrusion printing enables the fabrication of aligned, cell‐laden filaments with a resolution of a few hundred micrometers. For example, Choi et al. developed muscle constructs with aligned outer shapes by using a gelatin/PVA granule‐based supporting structure. This setup facilitated the direct bioprinting of human skeletal muscle cells embedded in skeletal muscle dECM bioinks (Figure 11a).^143^ At the microscopic level, however, the filament resolution is typically too large to provide the necessary topographical cues for fine‐scale cell alignment. To address this, Kim et al. incorporated fibrillated PVA into a bioink containing photocrosslinkable ECM derivatives from porcine skeletal muscles. This bioink served as a sacrificial material (Figure 11b) and enabled the creation of muscle repair constructs with uniaxially aligned microstructures, improving cell organization and promoting muscle regeneration.^85^

#### 5.2.5 Cartilage bioprinting

**FIGURE 12:**
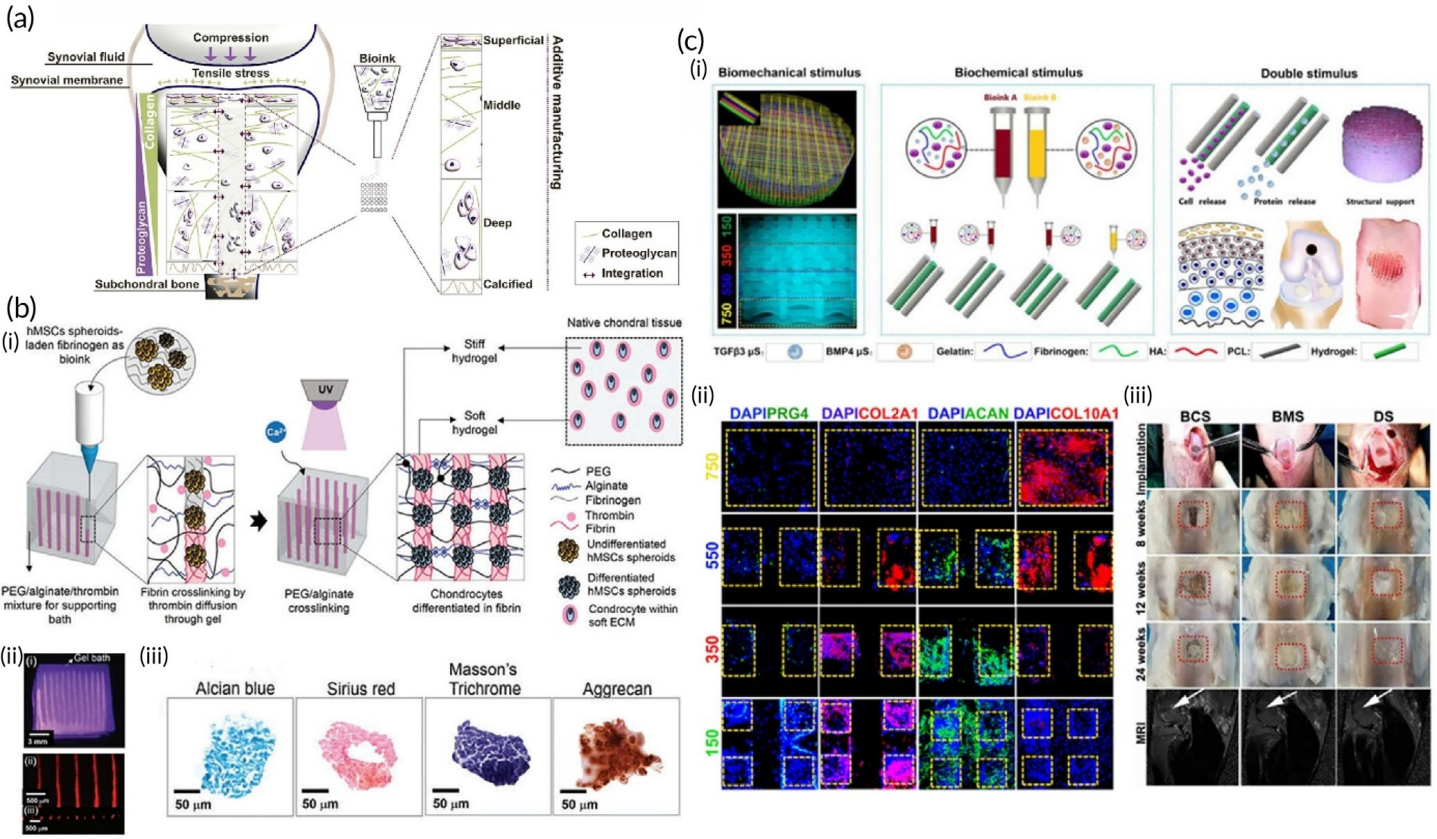
Schematic summary of 3D bioprinting strategies for region specific cartilage tissue engineering. (a) Three‐dimensional (3D) bioprinting concept based on regional extracellular matrix (ECM) composition and microstructure directs cell morphology and organization.^148^ (b) 3D bioprinted constructs with spatially controlled mechanical properties support cartilage‐like tissue formation in a chondrogenic environment.^149^ (c) Site‐specific microenvironment created by multichannel bioprinting enhances regional protein expression and implant function in articular cartilage regeneration.^150^ HA, hyaluronic acid; hMSCs, human mesenchymal stem cells; PCL, poly(ε‐caprolactone); PEG, polyethylene glycol.

Cartilage, a type of connective tissue, has limited capacity for self‐repair. It is categorized into three main types: hyaline cartilage, fibrocartilage, and elastic cartilage. The ECM of these cartilage types mainly consists of proteoglycans (GAG) and collagen, though the content and proportion of each component vary across cartilage types. Hyaline cartilage, found in the trachea, bronchial walls, and articular cartilage (on the surface of bones), primarily contains type II collagen. Fibrous cartilage, present in intervertebral discs, the meniscus, glenoid pelvis, and certain tendons, is mainly composed of type I collagen fibers and supports movement flexibility and tissue protection. Elastic cartilage, located in the auricle, eustachian tube, epiglottis, and larynx, contains both collagen and elastic fibers. 3D bioprinting has shown considerable promise in reconstructing cartilage shapes tailored to patient‐specific needs. For example, Martínez Ávila et al.^145^ developed hybrid bioinks combining nanofibrillated cellulose with alginate, which demonstrated excellent shear‐thinning properties and fast cross‐linking abilities. Using medical imaging data, such as magnetic resonance imaging and CT, 3D‐printed cartilage models with customized shapes (e.g., ear and meniscus) were successfully created. The chondrocytes in these bioinks exhibited high viability after 7 days of culture. Recent studies have emphasized the critical role of bioink composition in regenerating specific forms of cartilage by creating different biochemical microenvironments. Daly et al.^146^ compared common biomaterials for cartilage regeneration, demonstrating that alginate and agarose HGs supported the development of hyaline‐like cartilage with type II collagen ECM, while GelMA and methacrylamide polyethylene glycol (PEGMA)‐based HGs promoted the formation of fibrocartilage with both type I and type II collagen ECM. Mouser et al.^147^ studied the effect of varying concentrations of HAMA in bioinks, finding that glycosaminoglycan and type II collagen production increased at HAMA concentrations of 0.25%–0.5%, while 1% HAMA led to fibrocartilage formation. Additionally, tissue‐derived dECM‐based HGs are considered ideal for cartilage bioprinting, as they contain essential ECM components that aid in cell adhesion, growth, and differentiation. Cartilage in the knee joint, including articular cartilage and the meniscus, is subjected to continuous mechanical forces (Figure 12a).^148^ As a result, there is a need for engineered cartilage tissues with strong mechanical properties. However, functional chondrogenesis of cells requires a softer microenvironment. To address this, de Melo et al.^149^ developed a 3D‐bioprinted cartilage construct with controlled mechanical properties using a novel supporting bath system (Figure 12b). hMSC spheroids were loaded into fibrinogen bioink, which provided a soft microenvironment mimicking cartilage matrix formation, and bioprinted into a PEG/alginate/thrombin HG bath. The printed construct exhibited mechanical properties similar to native cartilage, while maintaining high cell viability and chondrogenic behavior without compromising the engineered ECM's mechanical properties. To further enhance mechanical strength and functionality, researchers have also explored the alternating use of cell‐laden bioinks and structural support bioinks. For example, Pati et al. created articular cartilage constructs by alternating dECM bioink (derived from cartilage) with PCL support bioink. This approach provided a favorable microenvironment for cartilage regeneration and long‐term function. Similarly, Sun et al.^150^ achieved anisotropic cartilage regeneration by bioprinting MSC‐laden constructs with gradient structures and zone‐specific chemical microenvironments. The printed scaffolds had varying filament spacing from 150 to 750 μm, creating a gradient structure that promoted articular chondrocyte differentiation in the superficial zone and enhanced nutrient diffusion and oxygen stress tolerance in the deep zone. The precise delivery of growth factors, such as TGF‐β3 and BMP‐4, to different layers of the construct further supported tissue regeneration and chondrogenesis (Figure 12c).

**FIGURE 13:**
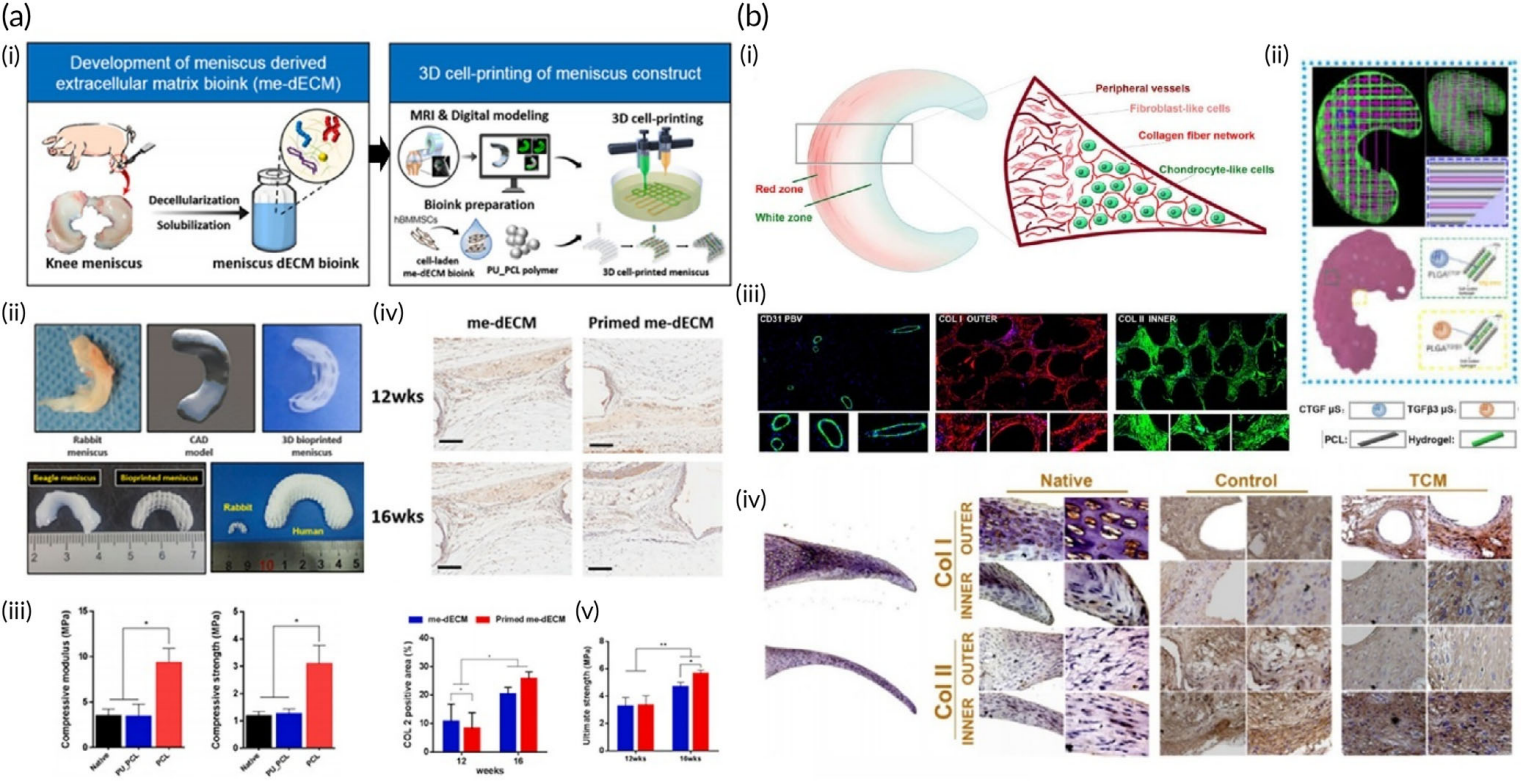
Schematic summary of 3D bioprinting strategies for meniscus tissue engineering. (a) Schematic summary: (a) Meniscus constructs prepared using multichannel bioprinting enhance type II collagen expression while maintaining mechanical integrity through the printing process and bioink design, and exhibit durability after 12–16 weeks of subcutaneous implantation.^151^ (b) Anisotropic bioprinted constructs mimic the regional cell types and collagen organization of the meniscus, supporting the formation of fibrocartilage, articular cartilage, and vascularization, and confirming functional tissue development after 24 weeks of implantation.^144^ 3D, three‐dimensional; CAD, computer‐aided design; dECM, decellularized extracellular matrix; PCL, poly(ε‐caprolactone).

The meniscus consists of semicircular cartilage with an anisotropic microstructure, which is essential for its mechanical strength and function. To engineer a tissue‐based meniscus with both robust mechanical properties and high bioactivity, researchers have developed bioprinting techniques. For example, Chae et al.^151^ used a two‐channel bioprinting method. One channel extruded a polymer mixture of polyurethane and PCL to provide structural integrity, while the other extruded stem cell‐laden decellularized meniscal ECM bioink to support cellular growth and chondrogenic differentiation (Figure 13a). This approach mimicked the biochemical environment of the meniscus, supporting the growth of stem cells. The meniscus has different cellular compositions depending on its region: fibroblast‐like chondrocytes in the outer areas and articular chondrocyte‐like cells in the inner region. Disruption of this structure and the ECM is linked to conditions like osteoarthritis. Sun et al.^144^ employed a multichannel bioprinting approach to recreate the meniscus's anisotropic structure. In their design, different bioinks containing MSCs and growth factors were printed in specific regions to promote cartilage regeneration in the inner region and fibrocartilage formation, as well as blood vessel growth in the outer region. This strategy successfully supported the development of meniscus‐like tissue both in vitro and in vivo, showing promise for regenerative treatments (Figure 13b).

**FIGURE 14:**
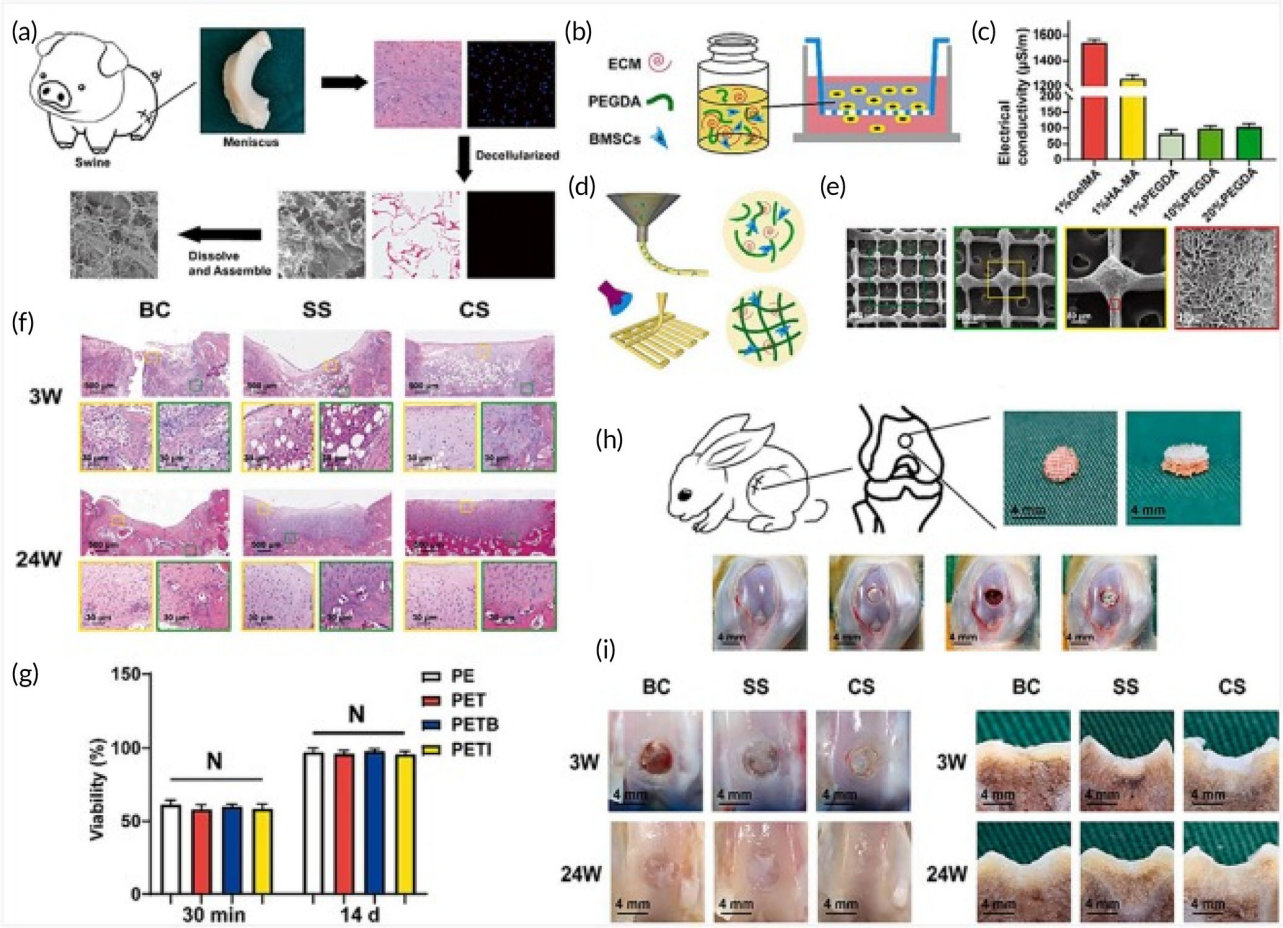
Schematic summary of porcine meniscus tissue engineering using ECM based and hydrogel scaffolds: Porcine meniscus extracellular matrix (ECM) production, cell invasion, biomaterial design, hydrogel properties, and implantation results were demonstrated by various methods: (a) ECM production and meniscus morphology were examined by macro‐observation, microscopy, scanning electron microscopy, and HE/DAPI (Hematoxylin and Eosin/4′,6‐Diamidino‐2‐Phenylindole) staining; (b) Cell invasion assay was performed with the Transwell system; (c–e) methacrylated hyaluronic acid (HAMA) and polyethylene glycol diacrylate (PEGDA) hydrogel were electro‐mesh and photocross‐linking processes, and the PEGDA/ECM scaffold structure was characterized; (f, g) histology and bone marrow‐derived stem cells (BMSC) viability were evaluated in the blank control, simple, and composite scaffold groups; (h, i) The integrity and textural response of the scaffold were observed after 3 and 24 weeks using the animal model and implantation procedure.^152^ CS, composite scaffold; SS, simple scaffold.

Cartilage is a smooth, elastic connective tissue found in various parts of the body, including the spine, ribs, joints, nose, ears, and trachea. It is composed primarily of collagen fibers, proteoglycans, glycosaminoglycans, and has a high water content. Cartilage serves essential supporting functions and, during embryonic development, acts as a template for bone formation. In the adult body, it exists in three primary forms: hyaline cartilage (or articular cartilage), fibrocartilage, and elastic cartilage. Each type has varying levels of collagen II and proteoglycans, performing specific functions within different tissues. Cartilage is an aneural and avascular tissue, meaning it lacks nerves, blood vessels, and lymphatics, and contains low‐density chondrocytes, which are responsible for nutrient diffusion. However, cartilage has a limited ability to regenerate due to the non‐proliferative nature of chondrocytes and insufficient nutrient supply. As a result, cartilage defects, whether caused by congenital issues, trauma, aging, or surgery, can lead to impaired joint function and potentially result in disability. With advancements in 3D bioprinting, it is now possible to create scaffolds with precise porosity and mechanical properties that mimic the native cartilage structure. This technology allows for the creation of cell‐laden constructs that replicate the complex structure and composition of natural cartilage. For example, Han et al. developed a hybrid bioink made from PEGDA and ECM components, incorporating bone marrow‐derived stem cells (BMSCs) and growth factors. This bioink was used in an electro‐writing (EW) bioprinting technique to produce a highly porous scaffold designed to mimic the layered structure of native cartilage, offering a promising approach for cartilage repair. In a rabbit model of femoral intercondylar cartilage injury, various scaffolds were evaluated for their ability to repair cartilage defects. The scaffolds were successful in filling the annular cartilage defect between the femoral condyles. In the control group, where no treatment was applied, the cartilage defect remained unhealed even after 24 weeks, highlighting the inherent challenge of repairing cartilage damage. In contrast, both the simple scaffold (SS) and composite scaffold (CS) groups showed cartilage formation at the injury site after 12 weeks. Notably, the CS group exhibited significant repair at 24 weeks, with the injured site fully covered by cartilage, which integrated seamlessly with the surrounding tissue. Histological examination revealed successful cartilage regeneration in the CS group (Figure 14).^152^ Wei et al. developed 3D‐plotted cartilage scaffolds by layering a combination of sodium alginate and PVA precursors using a layer‐by‐layer approach. This process resulted in a porous 3D HG scaffold with an optimal pore structure that supported chondrocyte proliferation. Similarly, Beketov et al.^153^ employed extrusion bioprinting to fabricate de novo cartilage using a bioink composed of 4% collagen and primary chondrocytes. In vivo studies demonstrated that this bioink successfully formed cartilage within 5–6 weeks after implantation in subcutaneous sites.

#### 5.2.6 Musculoskeletal bioprinting

Both bone and cartilage have shown promising potential for regeneration using bioprinting techniques. Similar to skin, both in vitro and in situ approaches have been explored to successfully regenerate bone using bioprinting. For example, Qi et al. developed bioactive glass scaffolds integrated with calcium sulfate hydrate for bone regeneration. Their study demonstrated that these scaffolds supported the adhesion and proliferation of human MSCs, promoting the formation of natural bone tissue when implanted into rats.^154^ The newly formed bone tissue developed more rapidly compared to the control group. In addition to in vitro studies, in situ methods for bone regeneration have also been investigated. Keriquel et al.^155^ used LAB to fabricate a bone construct consisting of mesenchymal stromal cells, collagen, and hydroxyapatite (HA), aiming to address bone defects in a mouse model. Their research showed that the printed construct was highly functional and viable, exhibiting osteoblast formation and successful proliferation, resembling the natural bone tissue it was designed to replace. Bioprinting has also gained significant attention in cartilage regeneration due to cartilage's limited ability to naturally regenerate. This technique offers a promising solution to the challenges posed by cartilage degeneration. For instance, Cui et al. employed inkjet bioprinting to create a bioink containing chondrocytes and polyethylene glycol dimethacrylate (PEGDMA) into a 3D biopaper plug, which was cultured in a bioreactor for 6 weeks. The results showed that the bioprinted cartilage exhibited a higher collagen II to collagen I ratio compared to natural cartilage, suggesting that the chondrocytes underwent proper maturation and growth throughout the incubation period.^156^

#### 5.2.7 Tendon bioprinting

**FIGURE 15:**
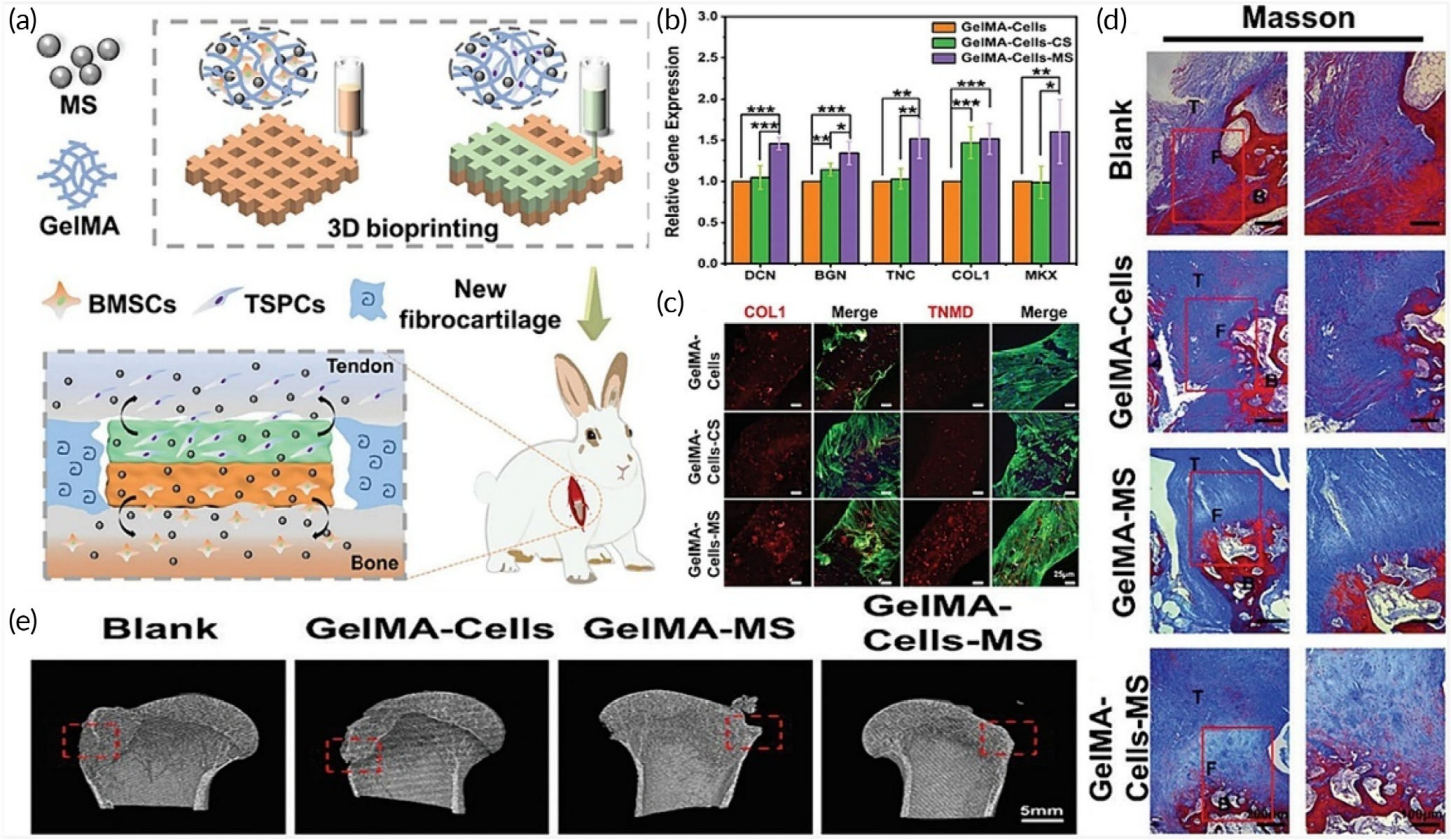
Schematic summary of biomimetic multicellular scaffolds for tendon bone interface repair: The use of biomimetic multicellular scaffolds in tendon‐bone interface repair is schematically shown (a); tenogenic differentiation gene expressions such as MKX, COL1, BGN (biglycan), TNC (Tenascin‐C), and DCN (Decorin) are examined (b); and tenomodulin (TNMD) and COL1 proteins are confirmed by immunofluorescence after 14 days of culture. *p < 0.05, **p < 0.01, ***p < 0.001. (c); a diagram of the tendon‐fibrocartilage‐bone interface after 12 weeks is presented (d), and micro‐computed tomography images show in vivo rabbit rotator cuff tear repair (e).^162^ GelMA, gelatin methacryloyl; TSPCs, tendon stem/progenitor cells.

Tendons and ligaments, composed primarily of fibrous collagenous connective tissue, are essential for connecting muscles to bones and maintaining joint stability while facilitating efficient load transfer between musculoskeletal tissues. Tendon injuries, which compromise tissue integrity and load‐bearing capacity, are particularly challenging to treat due to the tendons' limited ability to heal naturally. This is largely because tendons are avascular (lack blood vessels) and have low cellularity, meaning they have a limited capacity for regeneration and repair.^156^ Although significant advancements have been made in tendon TE, restoring tendon function after injury remains difficult, primarily due to the complex and heterogeneous structure of tendon and ligament tissue.^157^ 3D bioprinting presents a promising approach for overcoming these challenges, enabling the creation of scaffolds with precise pore sizes and geometries that more closely mimic the mechanical and biological properties of natural tendons and ligaments. For instance, Gottardi et al. engineered constructs with distinct tendon and cartilage components that replicate the tendon‐bone interface. These constructs, made using a highly biocompatible and superelastic PLGA scaffold, featured a microporous structure with aligned fibers on one side to mimic tendon tissue and a heterogeneous fiber arrangement on the other side to simulate cartilage. By using a biphasic bioreactor system, the scaffolds were cultured in a differentiation medium that simultaneously exposed both sides, supporting cell survival, differentiation, and proliferation. This scaffold showed promise for tendon repair, particularly after mechanical stimulation to assess its ability to withstand tensile stress, an important consideration before testing in vivo.^158^ In another study, Jiang et al. developed a novel approach for rotator cuff tendon repair using multilayered PLGA scaffolds combined with collagen‐fibrin HGs and adipose‐derived mesenchymal stem cells (hASCs). The scaffold promoted the growth, proliferation, and differentiation of hASCs into tendon‐like cells, with in vivo biocompatibility confirmed through subcutaneous implantation of the multilayered scaffolds. 3D bioprinting was essential in creating anatomically accurate cellular scaffolds and delivering cells and biomaterials to injured tendons and ligaments effectively.^159^ Beyond tissue repair, 3D bioprinting is advancing the development of platforms for drug screening and the fabrication of 3D tissue models in vitro. Laternser et al. combined 3D bioprinting with novel microplates to automate the creation of 3D musculoskeletal tendon‐like tissue models for drug screening.^160^ Another innovative approach in tendon regeneration involves projection‐based 3D bioprinting, which can rapidly generate HG microparticles of various sizes in a single layer. This technique helps protect encapsulated growth factors by eliminating the need for repeated elution or the use of organic solvents. For example, GelMA microparticles containing platelet‐rich plasma (PRP) and tendon‐derived stem cells (TDSCs) were created using this method and injected into a rat model of tendinopathy. The study found that PRP‐GM facilitated the proliferation and differentiation of TDSCs, showing favorable biocompatibility for tendon repair under chronic inflammatory conditions.^161^ To address the reconstruction of interfaces between soft and hard tissues, biomimetic multicellular scaffolds with bifunctional properties are being developed. These scaffolds can induce the regeneration of multiple tissue types, promoting the differentiation of tendon‐ and bone‐related cells into osteogenic and tenogenic lineages in vitro and supporting integrated regeneration of tendon‐to‐bone interfaces in vivo. Du et al. used a bilayered scaffold with Mo‐silicate bioceramics and stem/progenitor cells from tendons and bones to simulate tendon‐to‐bone interfaces. After 14 days of culture, tendon stem/progenitor cells (TSPCs) showed tenogenic differentiation, as evidenced by the expression of genes associated with tenogenesis. In vivo, these scaffolds demonstrated significant potential for enhancing tendon‐to‐bone regeneration, further supporting the potential of biomimetic strategies in complex tissue repair (Figure 15).^163^

#### 5.2.8 Bone bioprinting

**FIGURE 16:**
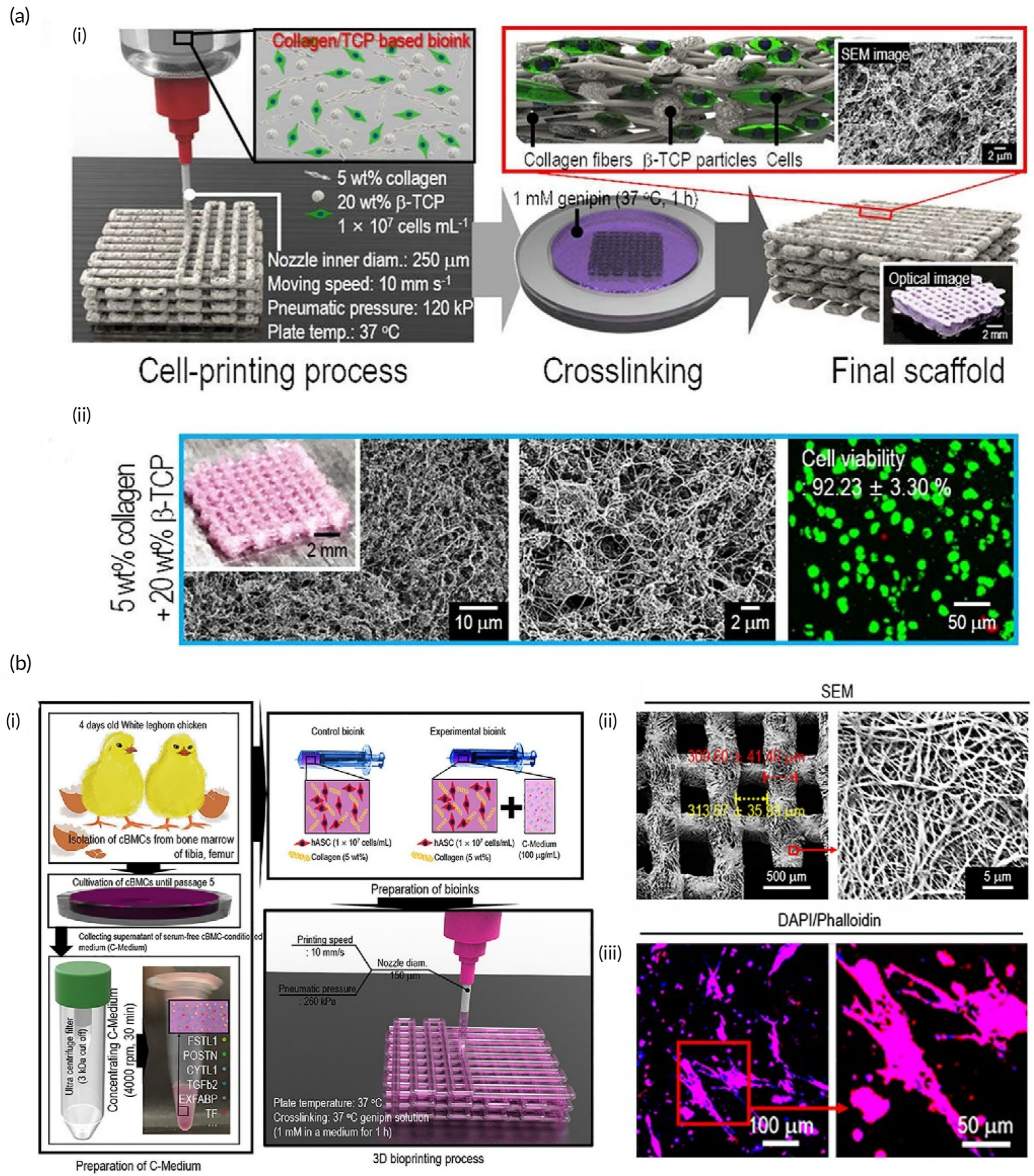
Schematic summary of 3D bioprinted cell‐laden scaffolds for bone tissue engineerin: (a) collagen/β‐calcium phosphate (TCP) based cell‐laden scaffolds were prepared after three‐dimensional (3D) bioprinting and cross‐linking to present a biomimetic microenvironment. Digital and scanning electron microscopy (SEM) images showed the shape and microstructure of the scaffold, while live/dead staining confirmed high cell viability.^166^ (b) Cell‐laden constructs containing bioactive components derived from chicken bone marrow cells were developed by the isolation of cBMC (chicken bone marrow cells)‐derived conditioned medium and human adipose stem cells (hASC)‐laden bioprinting process; microstructure and cell morphology were characterized by SEM and confocal microscopy.^167^

Bone has been named the greatest intelligent material historically and most precisely due to its limited regenerative flexibility.^164^ Although bone is known for its self‐healing capabilities, it cannot fully heal large defects beyond a critical size without medical intervention. Major causes of such bone defects are tumor resections and high‐impact trauma, which often necessitate bone repair and implantation in clinical settings. Autografts and allografts, although useful, have limitations in their availability and functionality, and inert implants may fail over time under repetitive stress. Tissue‐engineered bone, which can remodel into new bone to restore and improve its functions, has therefore become an increasingly important area of research. Thermal inkjet bioprinting has emerged as a promising technique that enables the deposition of cells, growth factors, and biomaterial scaffolds at precise 2D and 3D locations. The ejected droplets of ink, typically smaller than 0.03 mm, provide excellent resolution. Many inkjet‐printed scaffolds are composed of natural HGs to enhance cell compatibility. However, these natural HGs often lack the mechanical strength needed for bone tissue applications, limiting their use to softer tissues. Bone grafts have been developed using HGs like fibrin or alginate, but the compressive strength of these scaffolds, even after 4 weeks of culture, is insufficient for bone regeneration, with values lower than 5 kPa. In response to these limitations, a 3D bioprinting platform that uses a synthetic polymeric HG with simultaneous photopolymerization has been developed. This platform utilizes PEGDMA HGs that provide a compressive modulus of more than 500 kPa, significantly improving upon the 5 kPa provided by natural HGs. This modulus is comparable to that of human musculoskeletal tissues. Additionally, PEG HGs maintain cell viability and encourage ECM production. hMSCs, which are known to have osteogenic potential, can migrate to skeletal injury sites and differentiate into bone tissue when combined with suitable scaffolds. These cells are commonly used in orthopedic TE. hMSCs from bone marrow or adipose tissue can be induced to undergo osteogenic differentiation and form bone tissue when cultured with ceramic scaffolds, with bioactive glasses (BG) and HA enhancing this process. In the current study, these approaches were combined in a new bioprinting process, where hMSCs and PEGDMA were printed alongside BG or HA nanoparticles, creating bone constructs layer by layer. Biochemical analyses indicated significantly higher collagen production and enhanced alkaline phosphatase (ALP) activity in hMSCs printed with PEG‐HA scaffolds, promoting osteogenesis. Histological studies confirmed these findings, demonstrating that HA improves cell ALP activity and stimulates osteogenesis. The combination of PEG and HA scaffolds not only maintained hMSC viability but also promoted osteogenic differentiation and ECM production, demonstrating the feasibility of bioprinting bone tissue. This work highlights the potential to fabricate neobone tissue using hMSCs and osteogenic factors such as HA and BG nanoparticles within a durable PEG scaffold. The use of layer‐by‐layer assembly and simultaneous photopolymerization ensures that cells remain precisely positioned while minimizing phototoxicity. The findings pave the way for future advances in constructing osteochondral interfaces, an important yet challenging aspect of bone TE.^165^ Recently, Yang et al. introduced type I collagen into an osteocyte‐laden bioink that contained methacrylate hyaluronic acid (HAMA) to create 3D printed constructs with a biomimetic ECM. They analyzed the impact of this construct on the mineralization matrix production of osteocytes. The 3D microenvironment with type I collagen effectively enhanced osteocyte morphology markers, such as connexin43 and E11/podoplanin, as well as mineralization markers like dentin matrix acidic phosphoprotein 1, while also improving the cellular response to parathyroid hormone. As a result, type I collagen has emerged as a promising component in 3D printing bioinks for promoting osteogenesis and mineralization. Moreover, the introduction of bioceramic particles similar to the inorganic components of natural bone, such as HA and β‐tricalcium phosphate, has been found to promote the proliferation and osteogenic differentiation of encapsulated human stem cells in bioprinted constructs (Figure 16a).^166^ Cells in these composite microenvironments showed significantly higher osteogenic gene expression levels compared to those in purely organic bioinks. In addition to the biochemical signals from ECM, conditioned medium derived from bone marrow cells has been found to contain growth factors such as periostin and TGF‐β family members that support the expression of osteochondrogenesis‐related genes, including Col I, aggrecan, Sox9, osterix, Runx2, and osteopontin in human stromal cells. In another study, Yang et al.^168^ introduced chicken marrow cell‐derived bioactive components into human adipose‐derived stem cell (hASC)‐laden collagen bioink to construct 3D‐printed scaffolds. This approach demonstrated an effective promotion of bone repair by providing osteogenesis‐supporting biomaterial properties. Synthetic biomaterials containing functional ions or structures have also been developed for bone repair bioink preparation. Synthetic peptides with programmable amino acids were designed to self‐assemble into nanofibrous structures resembling the native ECM, regulating cell fate and behavior. Additionally, the inclusion of metal ions such as magnesium (Mg), strontium (Sr), and silicon (Si) in bioinks has been shown to facilitate rapid angiogenesis and bone regeneration. The incorporation of synthetic inorganic biomaterials like amorphous Mg phosphates and laponite nanoclay has significantly enhanced mineralization bioactivity and osteogenic gene expression in loaded stem cells within bioprinted constructs, providing a favorable microenvironment enriched with bioactive ions (Figure 16b).^167^

Bone bioprinting typically involves three key stages: (I) pre‐processing, (II) processing, and (III) post‐processing. The first phase, pre‐processing, focuses on planning all aspects related to the formation of the bioprinted tissue. This includes analyzing the anatomical structure of the targeted tissue through the use of technologies such as CT or MRI scans. Special software programs like AutoCAD, SOLIDWORKS, and CATIA are employed to create a 3D model of the tissue in layers based on the scanned data. In the processing phase, bioink is used for constructing the tissue. This bioink can include a combination of growth factors, cellular materials (such as ESCs, MSCs, or iPSCs), and both synthetic and cellular scaffolds to provide structure. Finally, the post‐processing phase involves incubating the tissue in a bioreactor to facilitate its maturation, preparing it for potential in vivo use. Another method for bone bioprinting follows a two‐step process. The first step involves synthesizing a methacrylated ethanolamide derivative of gelatin (GE‐MA), which is then mixed with HAMA to create a gel‐like fluid. This mixture is biocompatible and supports the adhesion and proliferation of cells, making it suitable for TE applications. The selection of bioink is a critical factor in bone bioprinting, as it must be tailored to the specific cell types used while ensuring cytocompatibility and biofunctionality. Various natural and synthetic materials have been explored for bioink preparation in this context. For instance, gelatin‐based bioinks cross‐linked with HA have been shown to mimic the natural composition of bone, with HA significantly increasing the viscosity of the bioink. However, synthetic HA typically has lower osteogenic activity compared to living tissue. The osteointegrative properties of HA can be enhanced by incorporating ions such as Mg and Sr. Additionally, HA can be combined with biodegradable polyesters like PCL to improve its mechanical properties or with alginate to achieve better biological performance. Another approach for bioink formulation involves using sodium alginate and poloxamer to enhance both mechanical strength and rheological properties. Another innovative method involves blending PCL microfibers and NFs with collagen and alginate, aiming to create a functional 3D construct that supports the proliferation and differentiation of MSCs. A summary of recent bone bioprinting studies focuses on the bioinks used and their applications in bone tissue printing.^90^ In a different approach, Koch et al. used laser‐induced forward transfer (LIFT) bioprinting to deposit NIH_3_T_3_ fibroblasts, HaCaT keratinocytes, and hMSCs. Their studies suggested that the stem cells may reduce cell viability when incorporated. To further explore native tissue formation, Koch and colleagues printed cells within a collagen matrix using LAB, showing that cells maintained vitality and proliferated within the printed structures while preserving the intended pattern. In a recent study, Albanna et al. demonstrated a proof‐of‐concept for a clinical skin bioprinter that was capable of printing autologous skin cells directly onto full‐thickness wounds to facilitate healing. Their approach involved combining dermal fibroblasts and epidermal keratinocytes with a fibrin/collagen HG carrier that supported cell viability and precise deposition, enabling the formation of a multilayered, multicellular skin structure. The results were promising, as they observed that 3–6 weeks after printing, human fibroblasts, keratinocytes, and endogenous cells remained viable. Notably, they discovered that re‐epithelialization had occurred in the bioprinted wounds, and the wound was fully healed after 6 weeks.^62^

### 5.3 The role of bioprinting in cancer research

Cancer remains one of the leading causes of death worldwide, with significant mortality rates and treatment‐associated challenges. 3D printing has emerged as a transformative technology in healthcare, demonstrating its ability to produce tissues, organs, and cellular constructs. In cancer treatment, this innovative approach has proven valuable by enhancing surgical precision and enabling personalized care. Surgeons can utilize 3D‐printed models of cancerous tissues to analyze and plan procedures more effectively, improving safety and accuracy during invasive surgeries. Patient‐specific models are considered the gold standard for complex cancer surgeries, allowing for tailored interventions. The application of 3D printing extends beyond surgery, aiding in predicting recovery outcomes and reducing surgical risks. By using individualized tumor models, clinicians can make more informed treatment decisions. These models provide detailed insights into a patient's anatomy and play a crucial role in the development of personalized anti‐cancer drugs. Despite its promise, a significant challenge remains: integrating 3D printing into the creation of effective therapeutic strategies. Researchers have employed 3D‐printed tumor constructs to improve diagnostic accuracy and evaluate cancer progression, enabling better‐targeted treatments and improved patient outcomes. Furthermore, 3D‐printed surgical guides have facilitated chemotherapy procedures, while customized medical devices have enhanced the precision of tumor excisions. The future of 3D printing in oncology holds immense potential, including its ability to fabricate intricate 3D cellular models for personalized treatments (Table 2). One promising development involves 3D‐printed biodegradable implants activated by laser therapy, offering a combination of chemotherapy and thermal ablation as a novel cancer treatment method.^187^ Another advancement is the creation of 3D porous scaffolds for addressing locally recurrent breast cancer. These scaffolds, constructed from biocompatible materials, support tissue repair after surgical removal of tumors, underscoring their therapeutic value. Cancer‐associated cachexia (CAC) is a debilitating condition characterized by systemic inflammation, weight loss, and adipose tissue degradation, significantly affecting patients' quality of life and contributing to 20%–30% of cancer‐related deaths. The severity of fat loss correlates with poorer survival outcomes, necessitating an in‐depth understanding of CAC mechanisms. Using 3D bioprinting, researchers have developed an engineered human white adipose tissue (eWAT) model induced with pancreatic cancer CM to mimic CAC conditions. CM induction increased lipolysis and ECM buildup within the model, while vascularization of eWAT (veWAT) enhanced angiogenesis and reduced glycerol release. The vascularized model showed elevated UCP1 expression, suggesting metabolic and inflammatory changes linked to CAC progression. Inflammatory cytokines such as IL‐8, CXCL‐1, and GM‐CSF in CM were identified as contributors to lipolysis and ECM remodeling, while eWAT secreted adipokines like NGAL and CD54, associated with cancer metastasis and muscle atrophy. These findings highlight the utility of the eWAT model in studying CAC and identifying potential therapeutic interventions. Overall, 3D bioprinting represents a groundbreaking tool for improving healthcare outcomes, offering personalized and efficient solutions tailored to individual patients. Its integration into cancer treatment enables the development of patient‐specific implants, prosthetics, and therapeutic strategies, advancing regenerative medicine and cell‐based therapies. Additionally, bioprinted tumor models derived from patients can significantly reduce the cost and time of drug discovery while minimizing treatment errors and side effects, particularly for patients with comorbidities.^188^

**TABLE 2:** Summary of other applications of bioprinting in cancer tissue engineering.

Cancer type	Bioink composition	Bioprinting method	Observations	Refs.
Ovarian cancer	Ovarian cancer cells + Matrigel	Pneumatic cell droplet	Micropatterning ovarian cancer cells (OVCAR‐5) and fibroblasts (MRC‐5) with spatial control, characterization of acini growth kinetics	169
Liver cancer	Hepatic carcinoma cells + Matrigel	Pneumatic extrusion	Radiation shielding capabilities of the prodrug amifostine, benefits in a dual‐cell model	170
Brain cancer	Endothelial cells + glioma stem cells + collagen/laminin	Extrusion	Tumor microenvironment of glioma/vascular system with dynamic flow to model cell–cell interaction of neoplastic glioma cells and ECs	171
Brain cancer	U118 glioma + pluripotent stem cell‐derived neural organoid	Extrusion	Invasion of human tumor cells using different neural progenitor cell lines, cell‐tracking dyes, and 3D laser scanning confocal microscopy	172, 173
Cervical cancer	HeLa/10 T1/2 + polyethylene glycol diacrylate	Projection stereolithography	Comparison between cancerous and non‐cancerous cell lines (HeLa vs. 10 T1/2)	174
Oral cancer	β‐Tricalciumphosphate	Extrusion	Incorporating oral squamous cell carcinoma (OSCC) cell line spheroids into a 3Dbioprinted model to depict the stages of oral cancer	175
Cervical cancer	HeLa + gelatin/alginate/fibrinogen	Extrusion	Viability, proliferation, matrix metalloproteinase (MMP) expression, and chemoresistance	176
Breast cancer	Breast adenocarcinoma + mouse macrophage + sodium alginate	Coaxial extrusion	Tumor microenvironment to explore migration of segregated tumor cells and macrophages (>90% viability)	177
Breast cancer	Breast cancer cells + (fetal osteroblasts/mesenchymal stem cells) + gelatin methacrylate	Stereolithography	Observation of interactions between BrCa and MSC/osteoblasts, and VEGF secretion in an artificial bone microenvironment	178
Breast cancer	Breast cancer cells + poly(ethylene glycol)	Continuous 3D projection	Breast cancer spheroids showed hypoxic cores and signs of necrosis, key features of the tumor environment.	179
Breast cancer	MCF‐7 + poly(ethylene glycol)	Inkjet	In situ cell seeding for the formation of breast cancer cellular spheroids and analysis as a potential microenvironment (>90% viability)	180
Brain cancer	Human glioma stem cells + gelatin/alginate/fibrinogen	Extrusion	Tumor microenvironment with over 87% cell viability; potential for vascularization, tumor angiogenesis, and VEGF secretion	181
Brain cancer	Glioblastoma‐associated macrophages + glioblastoma multiforme + gelatin methacryloyl/gelatin	Extrusion	In the printed mini‐brains, glioblastoma cells actively recruited macrophages and polarized them into a GAM (Glioma‐Associated Macrophages)‐specific phenotype. Also, macrophages induced glioblastoma cell progression and invasiveness	182
Brain cancer	GSC23 + U118 + sodium alginate	Coaxial extrusion	Glioma microenvironment evaluation for invasion and drug screening	183
Brain cancer	GSC23 + hMSCs + sodium alginate/gelatin	Coaxial extrusion	Tumor–stroma cells interaction, transcription of red fluorescent protein (RFP)	184
Breast cancer	Breast cancer cells + gelatin	Laser‐assisted	Laser direct writing on rat mesentery tissues, quantitative study of cancer cell activity, angiogenesis, and lymphangiogenesis	185
Cervical cancer	HeLa + collagen (printed on a nanofibrous membrane in co‐culture with fibroblasts)	Inkjet	Matrix metalloproteinase 2 (MMP2) and matrix metalloproteinase 9 (MMP9), drug screening	186

Abbreviations: 3D, three‐dimensional; ECs, endothelial cells; hMSCs, human mesenchymal stem cells; MSCs, mesenchymal stem cells; VEGF, vascular endothelial growth factor.

Although bioprinting has demonstrated remarkable potential in modeling cancer biology and advancing personalized therapeutics, several challenges remain before these approaches can be translated into routine clinical practice. A major limitation lies in the incomplete recapitulation of the tumor microenvironment, as current bioinks and printing methods cannot fully mimic the dynamic interplay between cancer cells, stromal components, immune cells, and ECM remodeling. Moreover, while many bioprinted constructs achieve high cell viability in vitro, their long‐term stability, reproducibility, and scalability for high‐throughput drug screening remain limited. Patient‐derived bioprinted tumor models offer significant promise for precision medicine; however, variability in patient samples, ethical considerations, and standardization issues hinder their widespread adoption. In addition, regulatory and manufacturing challenges must be addressed before customized 3D‐printed implants or scaffolds can be safely and consistently applied in clinical oncology. Without addressing these translational barriers, the application of 3D bioprinting may remain confined to experimental and preclinical studies rather than achieving its envisioned clinical impact.

## 6 AI AND BIOPRINTING: PERSONALIZED HEALTHCARE SOLUTIONS AND FUTURE PERSPECTIVES

### 6.1 The development of bioprinting with AI and machine learning

The foundation of 3D bioprinting lies in the creation of precise 3D models, which start with advanced modeling applications. These applications save time and resources by enabling virtual prototyping before physical printing. The development of CAD technology played a pivotal role in this evolution. Sketchpad, the first CAD software, laid the groundwork for modern CAD systems, which have become indispensable in personalized medicine. Through CAD, 3D‐printed organs can be tailored to fit individual patient needs, enhancing the efficacy of treatments. The early 1990s marked a breakthrough when CAD technology was successfully employed to create 3D scaffolds for TE. Modern bioprinting software, such as CELLINK's HeartOS, DNA Cloud, and DNA Studio, continues to refine this process, ensuring precision in bioprinted constructs. For simpler designs, tools like TinkerCAD are popular, while advanced modeling and post‐processing may require specialized software like Meshmixer. These advancements demonstrate how AI and machine learning are seamlessly integrated into bioprinting workflows, enabling the creation of increasingly complex and functional tissues and organs. By leveraging AI, bioprinting systems can predict outcomes, optimize material usage, and enhance accuracy, ensuring better alignment with patient‐specific medical needs. This technological convergence of AI, CAD, and 3D printing has revolutionized the field, offering unprecedented opportunities for advancements in personalized healthcare and regenerative medicine.^189^

Rafieyan et al. applied AI and machine learning techniques using a comprehensive data set encompassing 1171 scaffolds comprising 49 cell lines, various biomaterials, and different printing conditions. Cell response, printability, and scaffold quality were predicted using K‐Means clustering and algorithms such as XGBoost, Gradient Boosting, and Random Forest. Furthermore, a fully connected neural network was developed, and its hyperparameters were optimized. The results demonstrate that AI and ML are powerful tools for optimizing 3D bioprinting processes and improving the design of bioprinted scaffolds.^190^

Lee et al., a machine learning‐based method to design 3D printable bioinks using naturally derived biomaterials. Atelocollagen was used to create the ink, which provides high shape fidelity thanks to its mechanical properties and low yield stress. Machine learning determined the relationship between the ink's mechanical properties and printability, and multiple regression analysis generated optimized bioink formulations. Finally, 3D constructs demonstrating high viability and proliferation were successfully fabricated using cell‐laden HGs.^191^

Nadernezdah et al., an interpretable machine learning approach to examine the printability of HG ink formulations for 3D bioprinting. By analyzing rheological data and printability scores for 180 different formulations, 13 critical rheological measurements were identified. The analyses enabled printability prediction without any bias regarding HG composition and type of additives, demonstrating that the collaborative nature of rheological measurements provides qualitative and physically interpretable guidance for designing new biomaterials.^192^

Oh et al., a rheology‐informed hierarchical machine learning (RIHML) model was developed to predict print resolution in extrusion‐based 3D bioprinting. The RIHML model was trained using a small data set of bioink properties and printing parameters; it predicted print resolution with lower error compared to conventional models. The model made accurate predictions for different printing parameters and concentrations of bioink components and demonstrated high generalizability even when new materials were added. These results demonstrate that the RIHML model is a powerful and flexible tool for optimizing print resolution in extrusion‐based bioprinting.^193^

In summary, machine learning and AI accelerate the development of bioink formulations, enabling systematic study of their properties and increased printing accuracy in 3D bioprinting. Combined with experimental validation, this approach offers more precise and functional bioprinting solutions for TE and regenerative medicine.

### 6.2 Personalized medicine: custom health solutions with bioprinting

The integration of 3D bioprinting with regenerative medicine is revolutionizing the field of personalized medicine. Many bioprinting techniques and stem cell therapies depend on biological molecules, such as growth factors, to promote cell proliferation and differentiation. However, the high costs and potential toxicity of these molecules have encouraged researchers to seek alternative solutions. Medicinal plant extracts, which have been utilized in traditional medicine for centuries, are emerging as promising candidates to support stem cell therapies and TE. Recent research has demonstrated the potential of plant‐derived compounds, such as resveratrol, in enhancing the viability of chondrocytes for cartilage bioprinting. Extracts from plants like *Hypericum perforatum* and *Angelica* have shown encouraging results in promoting stem cell differentiation and tissue repair. For instance, flavonoids found in *Herba epimedii* have been proven effective in aiding the osteogenic differentiation of bone marrow‐derived MSCs, while compounds from *Salvia miltiorrhiza* have been associated with neurogenic differentiation, highlighting their potential in neural TE. Although medicinal plant extracts offer benefits like affordability and biocompatibility, challenges such as variability in composition, lack of standardization, and unclear mechanisms of action must be addressed. Ongoing research is focused on purifying and standardizing these extracts to maximize their clinical potential. Their cost‐effectiveness and safety profile make them particularly appealing for application in resource‐limited settings. By integrating medicinal plant extracts into 3D bioprinting processes, researchers aim to develop more affordable and non‐toxic solutions for regenerative therapies. This innovative approach combines traditional medicinal knowledge with modern technological advancements, paving the way for new solutions to complex medical problems. As this field evolves, it promises to bridge the gap between natural remedies and advanced scientific methods, expanding the horizons of personalized medicine.^194^

Sokmen et al. improved segmentation accuracy in vascular structures using transfer learning, resulting in bioprintable 3D models. Bifurcated coronary artery structures were produced with high accuracy using alginate‐glucomannan HG printing. While challenges such as material extrusion and shape accuracy were observed during the printing process, optimization techniques and multi‐axis print heads are expected to address these issues. The study demonstrates the strong potential of AI‐assisted 3D bioprinting for patient‐specific vascular implant production.^195^

Sarode et al.'s machine learning models were used to fabricate 3D bioprinted bone scaffolds and predict their compressive strength. The properties of the composite biomaterials, porosity, pore size, filament diameter, and scaffold printing architecture were used as inputs, while compressive strength was considered as the output parameter. The Hist Gradient Boosting Regressor model successfully predicted the outputs with the highest accuracy (95.33%) and lowest error (MAPE 7.69). This approach reduces human intervention, ensuring process optimization and reliability in the production of patient‐specific bone scaffolds.^196^

### 6.3 Data analytics and AI‐supported bioprinting

Advances in data analytics and AI are revolutionizing bioprinting, enabling groundbreaking enhancements in the development of 3D bioactive scaffolds. Data‐driven methodologies are pivotal in predicting cellular interactions, simulating biomaterial properties, and managing extensive data sets related to TE. AI algorithms assist bioprinters by simplifying complex biological processes, offering tailored design solutions for critical tasks such as biomaterial selection and cell arrangement. For example, deep learning models help refine the mechanical and biological characteristics of biomaterials, ensuring structural stability during printing and promoting long‐term compatibility. Additionally, predictive models powered by AI and data analytics facilitate real‐time process monitoring and error detection in bioprinting workflows. These systems analyze data continuously, pinpointing problems and dynamically enhancing print quality. AI‐based simulation tools are employed to assess the anatomical precision and functional suitability of tissue or organ replicas before they are fabricated, ensuring optimal results. In personalized medicine, AI integrated with patient‐specific biological data supports the creation of custom‐designed tissues and organs, reducing transplantation‐related compatibility issues and improving clinical outcomes. The incorporation of data analytics and AI into bioprinting not only accelerates the manufacturing process but also enables the production of intricate, functional, and patient‐specific biological structures. This integration broadens the potential applications of bioprinting in both research and clinical settings, laying the groundwork for transformative advancements in healthcare.^197^

Despite the remarkable synergy between AI and bioprinting, several critical limitations must be addressed to ensure their reliable integration into clinical practice. Current AI models, while powerful in optimizing design and predicting outcomes, remain dependent on the quality and diversity of input data. Biased or incomplete data sets may result in inaccurate predictions, compromising patient safety and treatment efficacy. Furthermore, the complexity of biological systems often exceeds the capabilities of current machine learning algorithms, raising concerns about oversimplification in modeling tumor microenvironments or organ‐level functions. Another challenge lies in the regulatory and ethical implications of AI‐driven decision‐making in healthcare: questions remain about liability if an AI‐assisted bioprinted construct fails. From a technical perspective, issues such as software interoperability, data privacy, and standardization across platforms hinder the seamless adoption of AI in bioprinting workflows. Without addressing these shortcomings, the translation of AI‐supported bioprinting from experimental settings to routine clinical use may face significant barriers. A balanced perspective is therefore essential; while AI undoubtedly enhances precision and personalization in bioprinting, its limitations highlight the need for cautious and evidence‐based implementation.

## 7 ETHICAL AND LEGAL CHALLENGES: RESPONSIBILITIES FOR THE FUTURE OF BIOPRINTING

The rapid evolution of 3D bioprinting technology holds the promise of revolutionizing medicine and bioengineering by enabling the creation of tissues and organs. However, these advancements also present significant ethical and legal challenges that demand careful consideration. Ensuring that bioprinting aligns with ethical principles and establishing a robust legal framework will play a critical role in its societal acceptance and long‐term success. The FDA has published a guidance document titled “Technical Considerations for Additive Manufactured Devices”, which outlines standards for the production of medical devices using AM techniques, including 3D printing. As printing technologies evolve and become more efficient and cost‐effective, it is increasingly important to establish stringent quality control standards. These standards should address every stage of the process, from model design and bioink selection to printing validation, post‐printing maturation, and final quality assessment.^198^ One significant challenge in 3D bioprinting is the complexity of the printing process, which involves numerous components. The lack of advanced software capable of accurately defining the virtual placement of cells, biomaterials, and biological molecules and translating these designs into effective downstream manufacturing processes poses a barrier to the field's progress.^199^ Another critical issue is ensuring that the bioprinted constructs are sufficiently stable and mechanically robust for transplantation. For applications like hard tissue repair, the structural design and porosity of the printed construct must maintain a high elastic modulus to support natural cell growth during implantation. Without proper mechanical support and structural integrity, scaffolds may deform, potentially causing the failure of newly formed tissues. Additionally, effective vascularization within bioprinted constructs is crucial for TE. Vascularization ensures that cells receive adequate oxygen, nutrients, and growth factors while facilitating the removal of waste. In vivo, capillaries located within 100 micrometers of cells allow sufficient diffusion, which is essential for cell survival. These challenges, along with scalability and widespread adoption hurdles in bioprinting techniques.^90^

### 7.1 Ethical challenges

Bioprinting technology raises a variety of ethical questions, including:

1. Source of cells and tissues: The use of human and animal cells in bioprinting necessitates ethical sourcing. Ensuring informed consent and transparency in obtaining biological materials is essential to maintaining public trust.
2. Creation of chimeric or hybrid organisms: Combining human and animal cells to produce hybrid tissues or chimeric organisms poses profound ethical concerns about the moral and social implications of such innovations.
3. Equitable access: Bioprinting has the potential to widen the gap between those who can afford cutting‐edge medical solutions and those who cannot. Addressing inequities in access will be vital to ensuring fairness.
4. Implications for human longevity: The possibility of extending human life through bioprinted organs raises philosophical questions about the limits of life extension and its impact on societal structures.

### 7.2 Legal challenges

The unprecedented nature of bioprinting demands a legal framework capable of addressing its complexities. Current international legal systems are often ill‐equipped to tackle these challenges, including:

1. Intellectual property rights: The patenting of bioprinting technologies and bioprinted organs raises questions about ownership and access. Determining the extent of rights over bioprinted organs is a contentious issue.
2. Regulatory standards for safety and approval: Clear guidelines are needed to assess the safety and efficacy of bioprinted tissues and organs for clinical use. Regulatory bodies must also establish liability protocols in case of failures.
3. Accountability and liability: When bioprinted organs malfunction or fail, defining who is responsible—manufacturers, healthcare providers, or patients—remains a complex legal issue.

### 7.3 Responsibilities for the future

To address these ethical and legal challenges, several responsibilities must be undertaken by the scientific community, policymakers, and society at large:

1. Developing global standards: International guidelines must be established to regulate the development and application of bioprinting technologies ethically and legally.
2. Engaging the public: Transparent discussions with the public about the societal and ethical implications of bioprinting will foster informed decision‐making and trust.
3. Encouraging interdisciplinary collaboration: Collaboration among scientists, ethicists, legal experts, and policymakers is crucial to crafting comprehensive solutions that balance innovation with accountability.

While 3D bioprinting offers groundbreaking opportunities, its ethical and legal dimensions cannot be ignored. Addressing these challenges proactively will be critical to ensuring that this transformative technology progresses responsibly. By anticipating its societal impacts and creating frameworks to mitigate potential risks, we can maximize the benefits of bioprinting while minimizing its downsides.^117^

### 7.4 Possible short‐term benefits of 3D bioprinting

#### 7.4.1 Short‐term benefits: reducing animal testing

The development of 3D bioprinting offers several short‐term applications, including reducing the reliance on animal testing. Innovations like “organ‐on‐a‐chip” technologies allow drug testing to be conducted on bioprinted human tissues rather than animals. Reprogramming an individual's cells into iPSCs enables the creation of organoids for testing drug efficacy and potency. This approach is not only more ethical than animal testing but is also expected to be more reliable since the testing is conducted on human tissues. Additionally, it opens the door to personalized medicine, allowing for tailored drug reactions and dosage adjustments based on individual responses, potentially minimizing adverse reactions.^189^

#### 7.4.2 Short‐term benefits: advances in tissue engineering

3D bioprinting has shown promise in TE, with advancements in creating artificial skin, cartilage, and trachea. Researchers are also working on bioprinting more complex structures like bones, ears, and heart valves. While relatively simple structures have been successfully engineered and even implanted, more complex solid organs remain a challenge. These applications, while limited to simpler constructs for now, contribute significantly to medical advancements.^156^

#### 7.4.3 Short‐term benefits: disease modeling

Another application is the bioprinting of diseased tissues for modeling purposes. Researchers can create cancerous or diseased tissue models using patient‐derived cells to test new treatments more accurately. This method holds the potential for developing highly effective therapies without directly involving patients, offering a safer and more precise approach to understanding disease mechanisms.^84^

### 7.5 Possible long‐term benefits of 3D bioprinting

#### 7.5.1 Avoiding ethical issues of animal use

In the long term, the fabrication of solid organs through bioprinting could eliminate the need for xenotransplantation and genetically modified animals like chimeric pigs. This development addresses ethical concerns surrounding the use of animals in organ generation. Public attitudes toward using animal organs for transplantation often reflect discomfort, emphasizing the need for alternatives that bypass these moral dilemmas.^200^

#### 7.5.2 Reducing financial and ethical costs of organ transplantation

The need for human organ transplants has created a demand that current donation systems cannot meet, leading to ethical and financial challenges. Bioprinting could provide a sustainable solution, reducing reliance on human donors and the illegal organ trade. It also offers the potential to significantly lower medical costs by eliminating the need for life‐long immunosuppressant therapy and associated complications.^201^

#### 7.5.3 Benefits for pediatric and young adult patients

Bioprinting offers particular promise for young patients, who often face difficulties obtaining organs due to the limited availability of suitable donors. The ability to create personalized organs could address these challenges, especially for conditions like heart valve disease, where current solutions have significant limitations for younger recipients.^202^

### 7.6 Ethical challenges in 3D bioprinting

#### 7.6.1 Social stratification and accessibility

One key ethical concern is the potential for unequal access to bioprinted organs. As technology evolves, it may only be accessible to wealthy individuals or nations, creating a tiered healthcare system where only the affluent benefit from advanced treatments. This raises critical questions about equity and the global distribution of healthcare resources.^203^

#### 7.6.2 Managing expectations

While simpler bioprinting applications, such as printing tissues or hollow organs, may be achievable in the near future, complex solid organs require significant advances in technology. Scientists face challenges in replicating the intricate structures of organs, including vascularization and tissue maturation. Unrealistic expectations could lead to public disillusionment or ethical concerns over resource allocation.^204^

#### 7.6.3 Risks of living cell implants

Bioprinting introduces the use of living cells, which carry risks like teratoma formation, cancer, or migration of implanted cells. Long‐term studies are necessary to fully understand and mitigate these risks. Furthermore, the use of embryonic stem cells (ESCs) for bioprinting raises ethical debates that need to be addressed.^205^

#### 7.6.4 Ownership and regulation of bioprinted products

The legal and ethical implications of bioprinted organs are complex. Questions about ownership, patentability, and the classification of bioprinted materials—whether as medical devices or living entities—must be resolved. Clear governance frameworks are essential to avoid exploitation and ensure equitable access.^206^

### 7.7 Critical perspective

While short‐ and long‐term benefits of 3D bioprinting highlight its transformative potential, it is crucial to acknowledge that many of these applications remain speculative and dependent on overcoming significant technical, biological, and regulatory barriers. For instance, disease modeling and drug screening on bioprinted tissues are promising but often fail to fully replicate the complexity of in vivo physiology, which limits predictive accuracy. Similarly, the vision of organ replacement through bioprinting faces hurdles such as vascularization, immune compatibility, and long‐term functional integration—challenges that remain unsolved despite optimistic projections. From an ethical standpoint, the rapid commercialization of bioprinted constructs without sufficient validation risks creating disparities in access and may amplify rather than reduce inequities in global healthcare. Furthermore, unresolved questions about intellectual property and ownership could concentrate control in the hands of a few private entities, raising concerns of monopolization. These limitations emphasize that while 3D bioprinting has immense promise, its path toward clinical reality requires a more cautious, evidence‐driven, and ethically informed approach than current narratives often suggest.

## 8 CONCLUSION

Bioprinting technologies, particularly those based on thermal inkjet printing, demonstrate great potential for creating living tissues with minimal damage, offering a range of applications from gene transfection to targeted drug delivery. These techniques are capable of fabricating both 2D and 3D tissue structures, including complex avascular and vascular tissues. One of the most promising clinical applications is the development of hand‐held, digital‐controlled printers for direct tissue repair, which could revolutionize how lesions are treated by precisely delivering cells, growth factors, and scaffolds tailored to the lesion's shape and thickness. Bioprinting's ability to produce complex, functional microvasculature is essential for the engineering of thick tissues, potentially paving the way for functional organ creation. Despite these advancements, significant challenges remain. One of the primary hurdles is the mechanical strength and integrity of the bioprinted constructs, especially those using HGs, which struggle to maintain structural integrity under external stress. The bioprinting of complex tissues, such as solid organs, is still in its early stages, as issues such as low resolution and insufficient vascularization impede progress. The fabrication of 3D tissue structures with high resolution, necessary for mimicking complex organ architectures, remains a technical challenge. Furthermore, the time required for printing large‐scale tissue constructs, especially when considering the speed limitations of extrusion‐based methods and the shear stress effects on cells, needs to be addressed for clinical use.^206^ To overcome these obstacles, future bioprinting research is focusing on the development of new materials with improved mechanical properties and biocompatibility. Advances in hybrid bioprinting techniques that combine multiple bioprinting methods are also a key research direction to improve the resolution and complexity of printed tissues. Additionally, strategies for promoting vascularization and innervation within bioprinted constructs are critical to ensuring the survival of thicker tissue constructs. Approaches such as volumetric bioprinting show promise for faster printing speeds, but further innovations are required to address compositional complexity. Other promising research areas include the integration of AI with bioprinting to enhance the quality control and precision of the manufacturing process, as well as the use of patient‐specific cells for regenerative medicine. Furthermore, advances in four‐dimensional (4D) bioprinting, which allows for the creation of dynamic structures that respond to external stimuli, hold potential for developing adaptive tissues and organs. The growing interest in organ‐on‐a‐chip technologies and personalized bioprinting also highlights the potential of these methods to create more accurate disease models and customized treatments for patients. While significant progress has been made in bioprinting, translating these technologies from the laboratory to clinical settings will require overcoming technical, biological, and regulatory challenges. Research on improving print resolution, vascularization, and cell differentiation is essential to advancing the potential of bioprinting in regenerative medicine. As these issues are addressed, bioprinting is expected to become an integral tool in the field of medicine, potentially leading to the fabrication of functional organs for transplantation and personalized medical treatments in the near future.^165^

## AUTHOR CONTRIBUTIONS

**SY**, **BS**, and **FC** contributed equally to this manuscript. **SY**, **BS**, and **FC** wrote the manuscript equally. **SY** and **BS** designed the structure of the manuscript. **SY**, **BS**, and **FC** reviewed and edited the manuscript. All authors have read and approved the manuscript.

## FUNDING INFORMATION

None.

## CONFLICT OF INTEREST STATEMENT

The authors declare no competing financial interests or personal relationships in this paper.

## Data Availability

No data sets were generated or analyzed during the current study.
